# Altered Fecal Metabolites and Colonic Glycerophospholipids Were Associated With Abnormal Composition of Gut Microbiota in a Depression Model of Mice

**DOI:** 10.3389/fnins.2021.701355

**Published:** 2021-07-19

**Authors:** Xue Gong, Cheng Huang, Xun Yang, Jianjun Chen, Juncai Pu, Yong He, Peng Xie

**Affiliations:** ^1^NHC Key Laboratory of Diagnosis and Treatment on Brain Functional Diseases, The First Affiliated Hospital of Chongqing Medical University, Chongqing, China; ^2^Department of Neurology, The First Affiliated Hospital of Chongqing Medical University, Chongqing, China; ^3^Chongqing Key Laboratory of Neurobiology, Chongqing, China; ^4^Department of Neurology and Stroke Center, The First Affiliated Hospital of Jinan University, Clinical Neuroscience Institute of Jinan University, Guangzhou, China; ^5^Institute of Life Sciences, Chongqing Medical University, Chongqing, China

**Keywords:** microbiota–gut–brain axis, chronic social defeated stress, gut microbiota, fecal metabolome, colonic lipids, glycerophospholipids

## Abstract

The microbiota–gut–brain axis has been considered to play an important role in the development of depression, but the underlying mechanism remains unclear. The gastrointestinal tract is home to trillions of microbiota and the colon is considered an important site for the interaction between microbiota and host, but few studies have been conducted to evaluate the alterations in the colon. Accordingly, in this study, we established a chronic social defeated stress (CSDS) mice model of depression. We applied 16S rRNA gene sequencing to assess the gut microbial composition and gas and liquid chromatography–mass spectroscopy to identify fecal metabolites and colonic lipids, respectively. Meanwhile, we used Spearman’s correlation analysis method to evaluate the associations between the gut microbiota, fecal metabolites, colonic lipids, and behavioral index. In total, there were 20 bacterial taxa and 18 bacterial taxa significantly increased and decreased, respectively, in the CSDS mice. Further, microbial functional prediction demonstrated a disturbance of lipid, carbohydrate, and amino acid metabolism in the CSDS mice. We also found 20 differential fecal metabolites and 36 differential colonic lipids (in the category of glycerolipids, glycerophospholipids, and sphingolipids) in the CSDS mice. Moreover, correlation analysis showed that fecal metabolomic signature was associated with the alterations in the gut microbiota composition and colonic lipidomic profile. Of note, three lipids [PC(16:0/20:4), PG(22:6/22:6), and PI(18:0/20:3), all in the category of glycerophospholipids] were significantly associated with anxiety- and depression-like phenotypes in mice. Taken together, our results indicated that the gut microbiota might be involved in the pathogenesis of depression via influencing fecal metabolites and colonic glycerophospholipid metabolism.

## Introduction

Depression is a debilitating mood disorder, characterized by the core symptoms of low mood, loss of interest, and even suicidal ideation (Cipriani et al., 2016). The lifetime prevalence of depression is evaluated to be around 20% and has been on the rise in recent decades, which has imposed not only a social and economic burden but is predicted to be a leading cause of global disability (Pu et al., 2020). Increasing studies have demonstrated that depression is greatly influenced by genetic and environmental factors (Li et al., 2016; Hüls et al., 2020). Various theories, such as neurotransmission disorder (Boonstra et al., 2015), endocrine imbalance (Liu S. et al., 2020) and immune dysregulation (Wu et al., 2016; Makris et al., 2020), have been proposed to explain the pathophysiology of this disease. However, antidepressant therapies based on these prevailing theories merely relieve clinical symptoms in about 40–50% of depressive patients (Warden et al., 2007; Voineskos et al., 2020). Thus, it is of great significance to find novel potential molecular mechanisms underlying depression.

Recently, the concept of the microbiota–gut–brain (MGB) axis has been proposed and garnered much attention with suggestions it may contribute to breakthroughs in understanding the etiology and pathophysiology of depression (Burokas et al., 2017; Wu et al., 2020). Several human studies have found disparities in gut microbiota composition between depressive patients and healthy controls at various taxonomic levels (Jiang et al., 2015; Chen J.-J. et al., 2020). At the phylum level, the relative abundances of *Firmicutes*, *Bacteroidetes*, *Proteobacteria*, and *Actinobacteria* were observed to be significantly altered. Similar changes were also observed in animal models of depression (Yu et al., 2017; Tillmann et al., 2019). Intriguingly, our previous work in rodents demonstrated that fecal microbiota transplantation from depressive patients to germ-free mice could induce depression-like behaviors in mice (Zheng et al., 2016). Other preclinical studies showed that supplementation with single- or multistrain probiotics, such as *Bifidobacteria* (Akkasheh et al., 2016; Tian P. et al., 2019), *Lactobacilli* (Akkasheh et al., 2016), and *Clostridia* (Tian T. et al., 2019), could modify emotional behaviors. These preliminary results revealed that gut microbiota plays a crucial role in the onset and progression of depression. Moreover, emerging evidence showed that gut microbiota may affect brain function via factors such as the vagus nerve (Marcondes Ávila et al., 2020), short-chain fatty acids (Stilling et al., 2016), and proinflammatory cytokines (Li et al., 2019). Nevertheless, the exact mechanism whereby gut microbiota influences the host remains elusive.

The gastrointestinal (GI) tract is a complex ecosystem inhabiting the majority of microorganisms in the human body (Eckburg et al., 2005). In the GI tract, small molecule metabolites produced by gut microbiota from undigested nutriment can be absorbed by intestinal epithelial cells, and subsequently provide energy and deliver external signals for the host (Macia et al., 2015; Rivière et al., 2016). Large intestine sites such as the colon show a higher diversity of microbiota (Gu et al., 2013), indicating it is an important site to explore the relationship between gut microbiota and the host. However, considerably less research is available regarding colon tissues and the metabolic profile inside.

Mounting evidence showed that metabolism disorder was involved in the MGB axis of depression, especially lipid metabolism disorder. Our latest findings revealed that microbiota dysbiosis was associated with disturbed hippocampal glycerophospholipid metabolism in female cynomolgus macaque who exhibited naturally occurring depression-like behaviors (Zheng et al., 2020). Consistently, we also observed disturbed lipid metabolism in the prefrontal cortex of germ-free mice (Chen et al., 2019). Thus, we hypothesized that gut microbiota might influence lipid metabolism in the colon as well, which might be an important link in the gut–brain interaction.

Thus, in this study, we established a mouse model of depression and applied multi-omics techniques (16S rRNA gene sequencing, non-target metabolomics, and lipidomics) to investigate the associations between the gut microbiota and GI tract. Our preliminary findings may enrich our understanding of the MGB axis in depression and provide useful information for further targeted experiments.

## Materials and Methods

### Animals and Ethics Statement

Male C57BL/6J mice (6–8 weeks of age, *n* = 20) were purchased from the experimental animal facility of Chongqing Medical University (Chongqing, China). Since the social defeat model of depression involves conflict stress (i.e., physical threat) from a more dominant resident counterpart, we also purchased CD1 male breeders (8 weeks of age, *n* = 40) to be used as aggressors for this investigation. Prior to defeat stress−exposure, CD1 aggressors were singly housed, while C57BL/6J mice were housed in groups of five, in standard polypropylene cages containing wood shavings. Mice were maintained in a standard condition with a 12-h light/dark cycle (lights on at 7:00 am), temperature of 22 ± 1°C, relative humidity of 40–60%, and with access to food and water *ad libitum*. Experiments were conducted in compliance with the National Institutes of Health’s Animal Research Guide, and with approval of the Ethics Committee of Chongqing Medical University. Efforts were made to use the minimum number of animals.

### Experimental Design and Behavioral Tests

**FIGURE 1:**
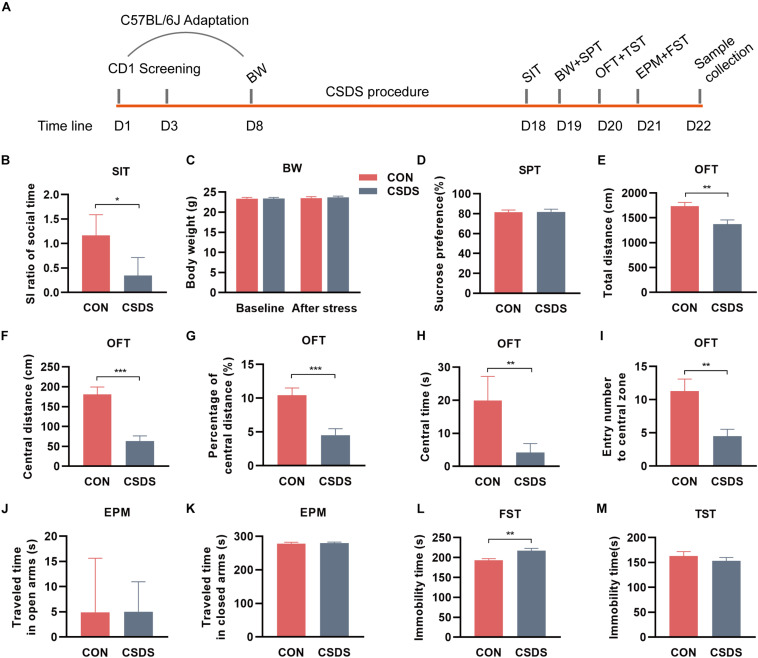
Experimental schedule and assessments of behavior indices. **(A)** Schedule of the CSDS procedure. During the first week, C57BL/6J mice were kept in experimental cages for environmental adaptation, and meanwhile, CD1 mice underwent the screening process. After the adaptation, C57BL/6J mice in the CSDS group were subjected to 10 consecutive days of CSDS exposure. After 4 days of behavioral tests (SI, BW, SPT, OFT, and FST), all C57BL/6J mice were sacrificed. **(B)** SIT: Mice in the CSDS group showed a significantly lower SI ratio than those in the control group. **(C)** BW: No significant difference was observed at baseline or after social defeated stress. **(D)** SPT: No significant difference was observed between the CSDS and control groups. **(E–I)** OFT: Mice in the CSDS group showed a significantly reduced total distance, central distance, percentage of central distance, central time, and entry number to the central zone than those in the control group. **(J,K)** EPM: No significant difference was observed in the time traveling in open or closed arms. **(L)** FST: Mice in the CSDS group showed a significantly increased immobility time than the control group. **(M)** TST: No significant difference was observed between the two groups. When the data conformed to normal distribution and homogeneity of variance, two-tail Student *t*-test was used and data are presented as mean ± SEM. Otherwise, the non-parametric Mann–Whitney *U* test was required and data are presented as median ± interquartile range (*n* = 10 mice in each group). *p < 0.05, **p < 0.01, ***p < 0.001, ns, no significance. CON, mice in the control group; CSDS, mice in the CSDS group.

The flowchart of the entire experimental schedule is presented in Figure 1A. Prior to the CSDS procedure, all CD1 mice were screened as aggressor candidates (Wang et al., 2016). Meanwhile, all C57BL/6J mice were acclimated in the experimental room for a week and randomly divided into control and CSDS groups. Then, the body weight (BW) of each C57BL/6J mouse was measured as baseline. After that, C57BL/6J mice in the CSDS group were subjected to social defeated stress for 10 consecutive days (Wang et al., 2016). Subsequently, all C57BL/6J mice underwent behavioral tests, including BW, a social interaction test (SIT), sucrose preference test (SPT), open field test (OFT), elevated plus maze (EPM), tail suspended test (TST), and forced swimming behavior test (FST). When behavioral tests were completed, C57BL/6J mice were anesthetized and then sacrificed by dislocation of the cervical vertebrae. Feces and colon tissues were collected, rapidly frozen with liquid nitrogen, and stored at −80°C until the assay. For the purpose of our study, CSDS-treated mice with an SI ratio ≥ 1 were considered stress-resilient and were excluded from subsequent multi-omics experiments. More details are presented in the Supplementary Materials.

### 16S Ribosomal RNA (16S rRNA) Gene Sequencing

The total microbial DNA from fecal samples (*n* = 10/8, control/CSDS) was extracted using the E.Z.N.A.^®^ soil DNA Kit (Omega Bio-tek, Norcross, GA, United States) according to the manufacturer’s instructions. After DNA concentration determination, purification, and quality evaluation, the V3-V4 region of the 16S rRNA gene was amplified with primers 338F and 806R (Zheng et al., 2019) by a thermocycler PCR system (GeneAmp 9700, ABI, United States). The PCR products were checked by 2% agarose gel electrophoresis, further purified by the AxyPrep DNA Gel Extraction Kit (Axygen Biosciences, Union City, CA, United States), and quantified by QuantiFluor^TM^-ST (Promega, United States). Then high-throughput sequencing was performed on an Illumina MiSeq platform (Illumina, San Diego, CA, United States) according to the standard protocols by Majorbio Bio-Pharm Technology Co. Ltd. (Shanghai, China). After quality filtering the obtained raw gene sequences, the remaining sequences with ≥97% similarity were classified into the same operational taxonomic units (OTUs) using UPARSE (version 7.1)^1^, and chimeric sequences were identified and removed using UCHIME. The taxonomy of each 16S rRNA gene sequence was analyzed by RDP Classifier^2^ against the Silva (SSU123) 16S rRNA database using a confidence threshold of 70%. More details are displayed in the Supplementary Materials.

The α-diversity was evaluated by the indexes: richness (Ace and Chao), evenness (Shannon), and global diversity (Simpson). The β-diversity was calculated based on weighted UniFrac algorithms and presented according to principal coordinate analysis (PCoA). Further, the differential bacterial taxa were identified by the linear discriminant analysis (LDA) effect size (LEfSe) method, and LDA scores ranked the dominant bacterial taxa responsible for discrimination between the two groups. The potential pathways of the altered gut microbiota were predicted using Phylogenetic Investigation of Communities by Reconstruction of Unobserved STates (PICRUSt) analysis based on the Kyoto Encyclopedia of Genes and Genomes (KEGG) database.

### Metabolome Analysis Based on Gas Chromatography-Mass Spectrometry (GC-MS)

This protocol was in accordance with the previous study (Yang Y. et al., 2019), with minor modifications. First, fecal samples (*n* = 10/8, control/CSDS) were dehydrated in a SpeedVac (Thermo SPD111V) for 3 h and weighed into 2 mL screw-cap tubes, for extraction. Then, 600 μL of the extraction solvent (80% methanol) containing internal standard (10 mM of L-alanine-2,3,3,3-d4) was added into each tube. Later, the stool-solvent mix was homogenized for 5 min and further dissociated by sonication. After centrifugation at 16,000 × *g* for 15 min, the supernatant was individually isolated and dried. Finally, the dried fecal extracts were derivatized based on a methyl chloroformate (MCF) approach and prepared for GC-MS analysis. More details about MCF and GC-MS protocols are displayed in the Supplementary Materials.

The raw mass spectra data obtained from GC-MS were processed by AMDIS software referring to the in-house library (detailed in the Supplementary Materials). The relative abundance of identified metabolites was adjusted to Gaussian distribution via log transformation prior to data analysis. The normalized data were introduced into SIMCA-P 14.1 software (Umetrics, Umeaå, Sweden) and then subjected to partial least-squares discriminant analysis (PLS-DA). Furthermore, a permutation test (200-iteration) was performed to assess whether there was a non-randomness of separation. By analysis of PLS-DA loadings plot, the metabolites with variable importance in projection (VIP) > 1.0 were considered key metabolites contributing to discrimination between the two groups. Meanwhile, Student’s *t*-test was used to calculate the *p*-value of each metabolite. Only metabolites with a *p*-value < 0.05 and VIP > 1.0 were considered to be significantly differential metabolites for subsequent pathway analysis (Yang L. N. et al., 2019). The predicted metabolic pathways were determined by our Pathway Activity Profiling R package based on the KEGG database (Han et al., 2019).

### Lipidome Analysis Based on Liquid Chromatography-Mass Spectrometry (LC-MS)

This process was conducted as our previous study (Chen et al., 2019). First, frozen colon tissues (*n* = 10/8, control/CSDS) were thawed at 4°C and then transferred into 2 mL screw-cap tubes. Secondly, 200 μL of pre-cooled ultrapure water, 240 μL of cold methanol, and 800 μL of MTBE were added to samples individually and the resultant mixtures were vortexed for 15 s, followed by sonication in an ice bath for 20 min, and rest at room temperature for 20 min. Later, the mixtures were centrifuged for 15 min at 8,000 × *g* at 10°C, and the upper organic phase was extracted and dried with nitrogen. Fourthly, dried samples were re-dissolved in 200 μL of isopropanol solution and vortexed. After centrifugation for 15 min at 8,000 × *g* at 10°C, the supernatants were prepared for LC-MS/MS analysis. More details about LC-MS/MS analysis are presented in the Supplementary Materials.

Lipid species were identified using the LipidSearch software version 4.1 (Thermo Scientific^TM^) and processed for peak alignment, retention time correction, and extraction peak area using a 5-ppm mass tolerance. For the data obtained from LipidSearch, molecules with RSD > 30% or missing values > 50% in the intra-group were removed. After normalization, data were uploaded to SIMCA-P 14.1 software (Umetrics, Umeaå, Sweden) for multidimensional analysis. Student’s *t*-test was used to calculate the *p*-value. Lipids with *p*-value < 0.05 and VIP > 1.0 were considered significant (Chen et al., 2019).

### Statistical Analysis

Data analyses were conducted using SPSS 21.0 (SPSS, Chicago, IL, United States) and plotted with GraphPad Prism 8.0 (La Jolla, CA, United States). For continuous variables that conformed to normal distribution, Student’s *t*-test was used to evaluate the significance; for non-normally distributed variables, non-parametric Mann–Whitney *U* test was applied. Spearman’s rank correlation analysis was used to determine the relationships among behaviors, microbiota composition, fecal metabolites, and colonic lipids. *p* < 0.05 was considered significant.

## Results

### CSDS Induces Social Interaction Deficiency and Anxiety- and Depression-Like Phenotypes

After exposure to 10 consecutive days of the CSDS procedure, mice in the CSDS group showed a lower SI ratio of social time in SIT (*z* = −2.206, *p* = 0.027; Figure 1B, indicating the social interaction deficiency behavior in the CSDS mice. There were two mice in the CSDS group with an SI ratio ≥ 1, so they were excluded from subsequent multi-omics experiments. However, no significance was observed in BW between the two groups at baseline or after stress (*t* = −0.062, *p* = 0.951; *t* = −0.325, *p* = 0.749; Figure 1C). Along with this, no difference was observed in SPT (*t* = −0.134, *p* = 0.895; Figure 1D). In OFT, mice in the CSDS group showed a dramatically reduced total distance (*t* = 3.136, *p* = 0.006; Figure 1E), central distance (*t* = 5.095, *p* < 0.001; Figure 1F), percentage of central distance (*t* = 4.240, *p* < 0.001; Figure 1G), central time (*z* = −3.178, *p* = 0.001; Figure 1H), and entry frequency to the central zone (*t* = 3.272, *p* = 0.004; Figure 1I), suggesting anxiety-like behavior in the CSDS mice. In EPM, no significant difference was observed in the time traveling in open or closed arms between the control and CSDS mice (*z* = 0, *p* = 1.000, Figure 1J; *z* = −0.227, *p* = 0.821; Figure 1K). In FST, the stress resulted in a significant elevation of immobile time (*t* = −3.549, *p* = 0.002; Figure 1L), suggesting depression-like behavior in the CSDS mice. However, there was no significant difference in the immobility of TST between the control and CSDS mice (*t* = 0.877, *p* = 0.392; Figure 1M). Collectively, these results indicated that the CSDS model of depression was successful established.

### CSDS Remodels the Composition of Gut Microbiota in Mice

**FIGURE 2:**
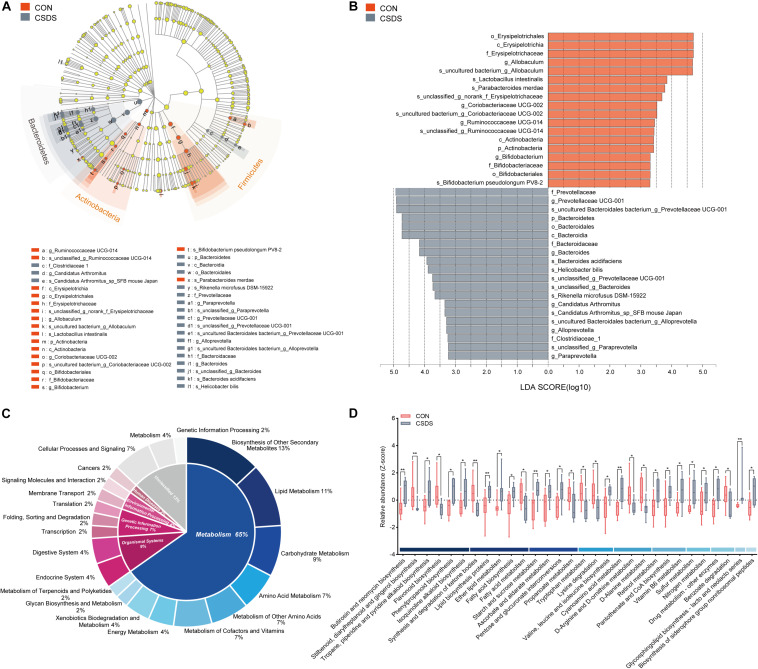
Comparison of the gut microbiota composition and functional predictions of the altered gut microbiota between the control and CSDS mice. **(A)** Phylogenetic diagram of LEfSe analysis illustrated the differential gut microbiota composition between the control and CSDS mice from the phylum to species levels. At the phylum level, Bacteroidetes were significantly enriched in the CSDS mice, whereas Actinobacteria and Firmicutes were enriched in the control mice. **(B)** Histogram of LDA scores computed by LEfSe analysis revealed the most differentially abundant bacteria taxa in the control and CSDS mice belonged to order Erysipelotrichales and family Prevotellaceae, respectively. **(C)** PICRUSt functional prediction of the altered gut microbiota indicated that the metabolism category ranked the top, with a proportion of 65%. In the metabolism category, lipid metabolism, carbohydrate metabolism, and amino acid metabolism counted for 11%, 9%, and 7%, respectively. **(D)** Histogram of all the significantly dysregulated metabolic pathways in the metabolism category. Mann–Whitney *U* test, *p < 0.05, **p < 0.01. CON, mice in the control group; CSDS, mice in the CSDS group.

To explore whether CSDS could alter gut microbiota composition, we employed 16S rRNA gene-sequencing. A total of 813395 high-quality reads were identified in 18 fecal samples with an average sequence length of 441.59. These reads were clustered into 673 OTUs (Supplementary Table 1). No significance was found in the α-diversity indexes including richness, evenness, and global diversity (Supplementary Figure 1), indicating that the within-sample diversity was not changed after CSDS treatment. However, the 3D-PCoA plot showed an obvious separation in gut microbiota composition between the control and CSDS mice (Supplementary Figure 2). To further identify the differential bacterial taxa contributing to the separation, LEfSe analysis was conducted. As depicted in the cladogram (Figure 2A), there were 38 differential bacterial taxa between the two groups in all, namely, 18 enriched in the control mice while there were 20 enriched in the CSDS mice. At the phylum level, *Actinobacteria* was dominant in the control mice while *Bacteroidetes* was dominant in the CSDS mice. Although no significance was observed in *Firmicutes* at the phylum level, 12 differential bacterial taxa belonged to *Firmicutes* from the class to species levels. The relative abundance of gut microbiota community on the genus level is illustrated in the Supplementary Figure 3. On the other hand, LDA scores computed by LEfSe analysis showed the individual contribution of differential taxa to the separation of the control and CSDS mice (Figure 2B and Table 1). The higher the LDA scores, the greater the contribution. In general, *order Erysipelotrichales*, *class Erysipelotrichia*, and *family Erysipelotrichaceae* were the top three taxa in the control mice; while *family Prevotellaceae*, *genus Prevotellaceae UCG 001*, and *species uncultured Bacteroidales bacterium Prevotellaceae UCG 001* were the top three taxa in the CSDS mice.

**TABLE 1:** Differentially abundant taxonomy between the control and CSDS groups analyzed by LEfSe (from phylum to species levels).

Taxon	Enrichment	LDA	*p*-
	in group	score	Value
o_Erysipelotrichales	CON	4.7044	0.0034
c_Erysipelotrichia	CON	4.7044	0.0034
f_Erysipelotrichaceae	CON	4.7044	0.0034
g_Allobaculum	CON	4.6789	0.0025
s_uncultured_bacterium_g_Allobaculum	CON	4.6789	0.0025
s_Lactobacillus_intestinalis	CON	3.8470	0.0209
s_Parabacteroides_merdae	CON	3.7799	0.0245
s_unclassified_g_norank_f_ Erysipelotrichaceae	CON	3.6864	0.0113
g_Coriobacteriaceae_UCG_002	CON	3.5212	0.0321
s_uncultured_bacterium_g_ Coriobacteriaceae_UCG_002	CON	3.5212	0.0321
g_Ruminococcaceae_UCG_014	CON	3.4485	0.0263
s_unclassified_g_Ruminococcaceae_ UCG_014	CON	3.4300	0.0025
c_Actinobacteria	CON	3.4179	0.0410
p_Actinobacteria	CON	3.4179	0.0410
g_Bifidobacterium	CON	3.3058	0.0087
f_Bifidobacteriaceae	CON	3.3058	0.0087
o_Bifidobacteriales	CON	3.3058	0.0087
s_Bifidobacterium_pseudolongum_PV8_2	CON	3.2994	0.0406
f_Prevotellaceae	CSDS	4.9406	0.0410
g_Prevotellaceae_UCG_001	CSDS	4.9099	0.0209
s_uncultured_Bacteroidales_bacterium _g_Prevotellaceae_UCG_001	CSDS	4.9070	0.0209
p_Bacteroidetes	CSDS	4.7297	0.0410
o_Bacteroidales	CSDS	4.7297	0.0410
c_Bacteroidia	CSDS	4.7297	0.0410
f_Bacteroidaceae	CSDS	4.1655	0.0059
g_Bacteroides	CSDS	4.1655	0.0059
s_Bacteroides_acidifaciens	CSDS	3.9261	0.0025
s_Helicobacter_bilis	CSDS	3.8861	0.0104
s_unclassified_g_Prevotellaceae_UCG_001	CSDS	3.7407	0.0045
s_unclassified_g_Bacteroides	CSDS	3.7289	0.0263
s_Rikenella_microfusus_DSM_15922	CSDS	3.6643	0.0390
g_Candidatus_Arthromitus	CSDS	3.3425	0.0099
s_Candidatus_Arthromitus_sp_SFB _mouse_Japan	CSDS	3.3425	0.0099
s_uncultured_Bacteroidales_bacterium _g_Alloprevotella	CSDS	3.2879	0.0462
g_Alloprevotella	CSDS	3.2854	0.0462
f_Clostridiaceae_1	CSDS	3.2430	0.0059
s_unclassified_g_Paraprevotella	CSDS	3.2280	0.0430
g_Paraprevotella	CSDS	3.2280	0.0430
			

*CON, control; CSDS, chronic social defeated stress.*

### Disturbed Gut Microbiota Composition Predicts Metabolism Disorder in the CSDS Mice

To determine whether the changes of gut microbiota composition induced by CSDS could lead to functional activity alterations, PICRUSt was used to predict the abundance of functional categories based on the standardized OTU abundance and KEGG database. In the first level of KEGG pathways, the microbial gene functions related to ‘metabolism’ ranked the top, accounting for 65% of the functional categories. Among the ‘metabolism’ category, ‘biosynthesis of other secondary metabolites,’ ‘lipid metabolism,’ ‘carbohydrate metabolism,’ ‘amino acid metabolism,’ ‘metabolism of other amino acids,’ and ‘metabolism of cofactors and vitamins’ were the top six metabolic pathways in the second level of KEGG pathways, consisting of 13%, 11%, 9%, 7%, 7%, and 7%, respectively (Figure 2C). The third level of KEGG pathways is illustrated in Figure 2D and Supplementary Figure 4. In the category of ‘lipid metabolism,’ ‘fatty acid biosynthesis,’ and ‘fatty acid metabolism’ were involved; in the category of ‘carbohydrate metabolism,’ ‘starch and sucrose metabolism,’ ‘pentose and glucuronate interconversions,’ and ‘propanoate metabolism’ were involved; in the category of ‘amino acid metabolism,’ ‘tryptophan metabolism’ was involved.

### Fecal Metabolic Analysis Indicates Metabolism Disorder in the CSDS Mice

**FIGURE 3:**
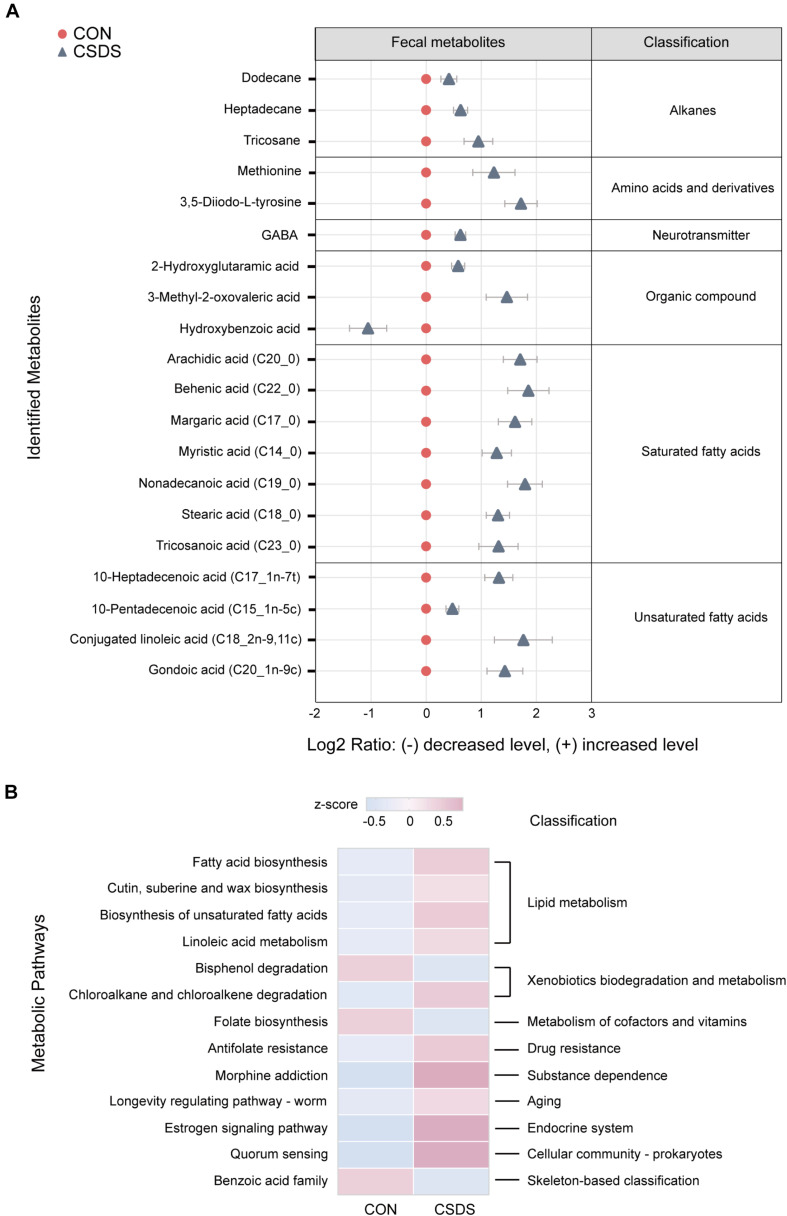
Identification and pathway analysis of differential fecal metabolites between the control and CSDS mice. **(A)** 20 fecal metabolites were identified to be significantly changed in the CSDS mice. Compared to the control mice, only one metabolite (hydroxybenzoic acid) was significantly decreased, whereas the other 19 metabolites were increased in the CSDS mice. **(B)** Pathway analysis for fecal metabolites based on KEGG database revealed that 13 pathways were disturbed in the CSDS mice, of which most (4/13) were involved in lipid metabolism. CON, mice in the control group; CSDS, mice in the CSDS group.

As the functional analysis by PICRUSt revealed that the metabolism functional category ranked the top, we accordingly employed the GC-MS non-targeted metabolomic technique to assess the contributions of the fecal metabolome. After data normalization, the PLS-DA model revealed a clear distinction between the metabolites in the control and CSDS groups (*R^2^Y* = 0.722, *Q*^2^ = 0.423; Supplementary Figure 5A). Moreover, the permutation plot indicated that the original PLS-DA model was valid and not over-fitted (*R*^2^ = 0.553, *Q*^2^ = −0.024; Supplementary Figure 5B). A total of 20 differential metabolites (*p* < 0.05 and VIP > 1.0), including 19 upregulated and one (hydroxybenzoic acid) downregulated, were identified. Among them, 11 metabolites (55%) were fatty acids (FAs, including saturated and unsaturated FAs), and the rest were alkanes, amino acids and derivatives, neurotransmitters, and organic compounds. Notably, γ-aminobutyric acid (GABA), as an important inhibiting neurotransmitter, was observed to be elevated in the CSDS mice (Figure 3A and Supplementary Table 2). A further pathway analysis showed that 13 pathways were significantly altered (Figure 3B), 4 (30.77%) were assigned to the category ‘lipid metabolism,’ including ‘fatty acid biosynthesis,’ ‘cutin, suberin, and wax biosynthesis,’ ‘biosynthesis of unsaturated fatty acids,’ and ‘linoleic acid metabolism,’ which were all enhanced in the CSDS group. Meanwhile, pathways related to ‘xenobiotics biodegradation and metabolism’ were significantly changed, with ‘bisphenol degradation’ downregulated but ‘chloroalkane and chloroalkene degradation’ upregulated in the CSDS group. Other pathways were involved in ‘folate biosynthesis,’ ‘antifolate resistance,’ and ‘morphine addiction,’ etc. Furthermore, a Venn plot based on the second level of KEGG pathways revealed that ‘lipid metabolism,’ ‘xenobiotics biodegradation and metabolism,’ and ‘metabolism of cofactors and vitamins’ were the overlapping categories of pathway analysis of gut microbiome and fecal metabolome. As for the third level, “fatty acid biosynthesis” was the only overlapping category (Supplementary Figure 6).

### Colonic Lipidome Reveals Disturbance of Glycerophospholipids, Glycerolipids, and Sphingolipids in the CSDS Mice

**FIGURE 4:**
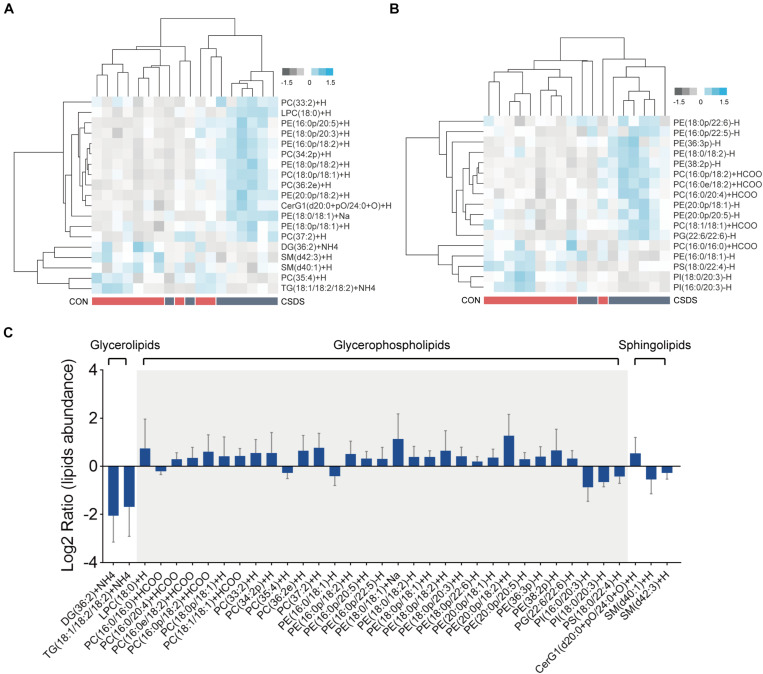
Differential lipids identified in the colon tissues between the control and CSDS mice. **(A)** Heatmaps of 19 differential lipids identified in the positive mode and **(B)** 17 differential lipids identified in the negative mode. **(C)** These differential lipids belonged to glycerolipids, glycerophospholipids, and sphingolipids categories, of which most (31/36) were glycerophospholipids. CON, mice in the control group; CSDS, mice in the CSDS group.

All lipids identified were classified into 30 classes (Supplementary Table 3). In the positive mode, the PLS-DA plot showed that the CSDS mice could be obviously separated from the control with no overlap (*R^2^Y* = 0.809, *Q*^2^ = 0.412; Supplementary Figure 7A); meanwhile, the permutation plot indicated that the original PLS-DA model was valid and not over-fitted (*R*^2^ = 0.649, *Q*^2^ = 0.100; Supplementary Figure 7C). After PLS-DA analysis and *t*-test, 19 differential lipids were identified (Figure 4A). In the negative mode, the PLS-DA plot also indicated a distinct separation between the two groups (*R^2^Y* = 0.926, *Q*^2^ = 0.479; Supplementary Figure 7B); the permutation plot showed that the original model was valid and not over-fitted (*R*^2^ = 0.877, *Q*^2^ = −0.008; Supplementary Figure 7D). Using the same method, 17 lipids in the negative mode were identified as significantly differentially expressed (Figure 4B). The 36 differential lipids belonged to glycerophospholipids, glycerolipids, and sphingolipids (Figure 4C and Supplementary Table 4). As shown in Figure 4C, when compared to the control mice, most glycerophospholipids (25/31) were upregulated in the depressed group, while all glycerolipids (2/2: DG, diglyceride; TG, triglyceride) were downregulated. In the sphingolipids category, glucosylceramide (CerG1) levels were increased in the CSDS mice, while the other two kinds of sphingomyelin (SM) were decreased.

### Correlations Between Differential Bacterial Taxa, Fecal Metabolites, Colonic Lipids, and Behaviors

**FIGURE 5:**
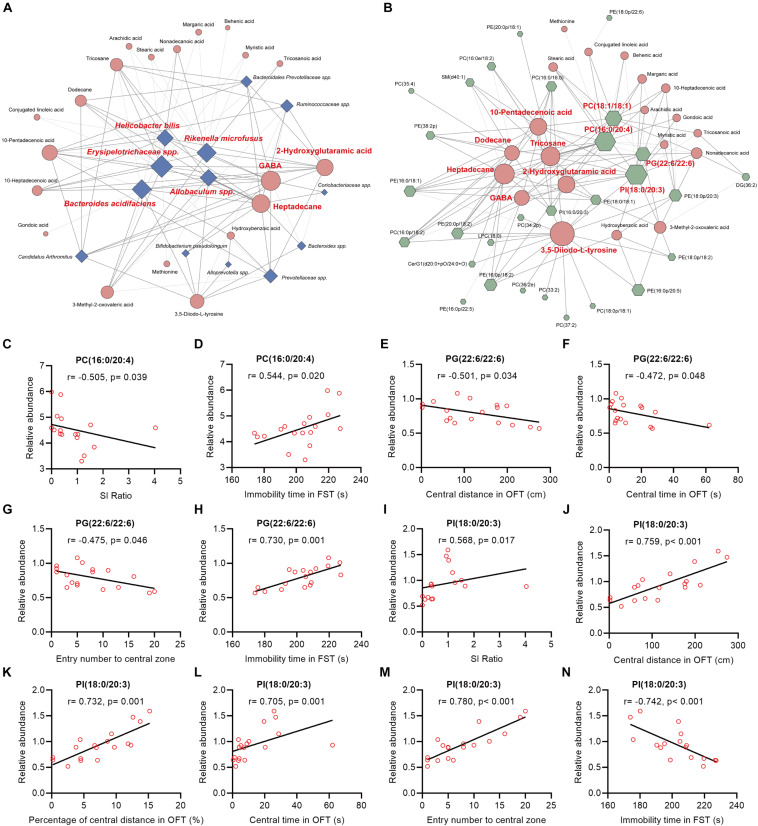
Spearman’s correlations between the differential bacteria species, fecal metabolites, colonic lipids, and behavior indices. **(A,B)** The network maps illustrated correlations between the differential bacteria species and fecal metabolites, and between the differential fecal metabolites and colonic lipids, respectively. Blue nodes represent bacteria species; orange nodes represent fecal metabolites; and green nodes represent colonic lipids. Edges between nodes represent Spearman’s correlation. The node size corresponds to the number of correlations, that is, a larger node for bacteria taxa indicates it linked to more fecal metabolites. Nodes (the number of correlations over or equal to 10) were marked in red. The edge width corresponds to correlation coefficient. **(C–N)** The correlation analysis between PC(16:0/20:4), PG(22:6/22:6), PI(18:0/20:3), and behavioral index, respectively.

In this study, we found that (Figure 5A, Supplementary Figure 8): (i) the majority (13/16) of bacterial species were significantly correlated with the 20 fecal metabolites to varying degrees; (ii) there were 5 bacterial species significantly correlated with 10 or more fecal metabolites: *species Erysipelotrichaceae* was negatively correlated with 14 fecal metabolites; *species Rikenella microfusus* was positively correlated with 12 metabolites; *species Bacteroides acidifaciens* was negatively correlated with 11 metabolites while positively correlated with hydroxybenzoic acid; *species Allobaculum* was negatively correlated with 10 metabolites; *species Helicobacter bilis* was positively correlated with 10 metabolites; (iii) there were 3 fecal metabolites significantly correlated with 10 or more bacterial species: GABA was negatively correlated with five bacterial species, and positively correlated with seven species; heptadecane was negatively correlated with five species, and positively correlated with six species; 2-hydroxyglutaramic acid was negatively correlated with three species, and positively correlated with seven species.

Intriguingly, we also found that (Figure 5B and Supplementary Figure 9): (i) the majority (26/36) of colonic lipids were significantly correlated with the 20 fecal metabolites to varying degrees; (ii) there were 7 fecal metabolites significantly correlated with 10 or more lipids: 3,5-Diiodo-L-tyrosine (DIT) was positively correlated with 17 lipids while negatively correlated with PE(16:0/18:1) and PC(16:0/16:0); alkane metabolites heptadecane, tricosane, and dodecane were correlated with 15, 14, and 10 lipids, respectively; unsaturated FA 10-pentadecenoic acid was positively correlated with seven lipids while negatively correlated with five lipids; 2-hydroxyglutaramic acid was positively correlated with eight lipids while negatively correlated with four lipids; GABA was positively correlated with seven lipids while negatively correlated with PE(16:0/18:1), PC(16:0/16:0), and PI(18:0/20:3); (iii) there were four colonic lipids correlated with 10 or more fecal metabolites: PI(18:0/20:3) was negatively correlated with 16 metabolites while positively correlated with hydroxybenzoic acid; PC(16:0/24:0) and PC(18:1/18:1) were positively correlated with 16 and 12 metabolites, respectively; PG(22:6/22:6) was positively correlated with 10 metabolites.

Further analysis revealed that the mentioned lipids PC(16:0/20:4), PG(22:6/22:6), and PI(18:0/20:3) were significantly correlated with mice behavioral index (Figures 5C–N): (i) the relative abundance of PC(16:0/20:4) in mice colon tissues was negatively and positively correlated with SI ratio and immobile time in FST, respectively (Figures 5C,D); (ii) PG(22:6/22:6) was significantly negatively correlated with central distance/time/entry numbers in OFT, whereas it was positively correlated with immobile time in FST (Figures 5E–H); (iii) PI(18:0/20:3) was markedly positively correlated with SI ratio and indices in OFT, whereas it was negatively correlated with immobile time in FST (Figures 5I–N).

## Discussion

In the present study, in order to investigate the effects of chronic stress on the gut microbiota and host metabolism and further elucidate the role of MGB axis in depression, we applied multi-omics techniques and conducted an integrated analysis in a mouse CSDS model of depression. As one of the most widely used models of depression, CSDS has different paradigms with distinct periods of stress, such as 10 days and 21 days (Pan et al., 2020; Lu et al., 2021). Compared to the 21-day paradigm, the 10-day paradigm has lower operation costs and an easier stress operation procedure, but higher resilience. In order to decrease the resilience to stress in our study, efforts were made to ensure participation of skilled and regular experimenters, successful screening of CD1 mice, and good control of stress intensity during the whole process (with a resilience ratio of 20%, 2 resilient mice in 10 defeated mice). Herein, we found that CSDS altered gut microbiota composition in mice. In total, there were 20 bacterial taxa and 18 bacterial taxa significantly increased and decreased, respectively, in the CSDS mice. Furtherly, microbial functional prediction demonstrated a disturbance of lipid, carbohydrate, and amino acid metabolism in the CSDS mice. We also found 20 differential fecal metabolites and 36 differential colonic lipids (in the category of glycerolipids, glycerophospholipids, and sphingolipids) in the CSDS mice. Moreover, Spearman’s correlation analysis showed that fecal metabolomic signature was associated with the alterations in the gut microbiota composition and colonic lipidomic profile. Meanwhile, three lipids [PC(16:0/20:4), PG(22:6/22:6), and PI(18:0/20:3), all in the category of glycerophospholipids] were significantly associated with anxiety- and depression-like phenotypes in mice. Our results indicated that the gut microbiota might be involved in the pathogenesis of depression via influencing fecal metabolites and colonic glycerophospholipid metabolism.

Mounting evidence has implemented the important role of the gut microbiota and MGB axis in the pathogenesis of depression, but the underlying mechanism of the gut–brain interaction remains unclear. It has been reported that the microbiome can affect a range of neurotransmitters, including GABA and glutamatergic neurotransmitters, which may affect brain activity directly (Duranti et al., 2020; Łoniewski et al., 2021). In the present study, we found that GABA was increased in the feces metabolome profile of the CSDS mice. Consistently, a recent study also found that the level of GABA was upregulated in the habenula of CUMS-susceptible rats (Tian et al., 2020). Meanwhile, a previous study reported that GABA-related genes were increased in the dorsolateral prefrontal cortex of patients suffering from major depression (Zhao et al., 2018). Further, antibiotics administration in specific pathogen-free mice caused altered GABA concentrations in blood (Mazzoli and Pessione, 2016; Strandwitz et al., 2019), indicating peripheral levels of GABA could be modulated by gut microbiota. Except for GABA, we also found that DIT, an endogenous halogenated derivative of L-phenylalanine, was increased in the feces of the CSDS mice. Similar to L-phenylalanine, DIT can significantly and reversibly suppress excitatory glutamatergic synaptic transmission through a series of presynaptic and postsynaptic mechanisms (Kagiyama et al., 2004). Glutamate (Glu) is a common excitatory neurotransmitter, and excessive Glu accumulation in the synaptic cleft can cause excitatory neurotoxicity (Hu et al., 2018). The serum level of Glu has been reported to be higher in patients with major depression (Chen H. et al., 2020), and Glu has been considered a novel target of pharmacological agents in the current treatment of treatment-resistant depression, such as Esketamine (Kasper et al., 2020). Accordingly, in the study, an increased level of DIT in feces might be a neuroprotective response to chronic stress. Given that the relative abundances of several bacterial species were significantly associated with the levels of GABA and DIT, these differential bacterial taxa might be involved in the development of depression by influencing the host neurotransmission system.

In the fecal metabolome profile, we also found that the levels of 11 FAs (including saturated and unsaturated FAs) in the feces of the CSDS mice were all increased, as compared with the controls. The findings were consistent with the results of microbial functional prediction and metabolome pathway analysis, that is, ‘fatty acid biosynthesis’ was enhanced whereas ‘fatty acid metabolism’ was attenuated in the CSDS mice. Nonetheless, previous findings of the differences in FAs in depressed patients or animal models are inconsistent. In terms of saturated FAs, it has been reported that stearic acid (C18:0) and myristic acid (C14:0) were significantly elevated in depressed patients’ plasma and post-stroke depressed patients’ urine, respectively (Liu X. et al., 2016; Xie J. et al., 2020). However, other studies reported that stearic acid (C18:0) was downregulated in the plasma and prefrontal cortex of rats exposed to chronic unpredictable mild stress (CUMS) (Li et al., 2010; Liu L. et al., 2016). In terms of unsaturated FAs, Liu Y. et al. (2016) observed higher levels of several unsaturated FAs, such as linoleic acid (C18:2), in the plasma of melancholic depressed patients; whereas Paige et al. (2007) found that linoleic acid (C18:2) was significantly decreased in the plasma of older depressed patients. In spite of these discrepancies, both the current study and previous studies indicated that FAs metabolism disorder may contribute to the development of depression. Studies have shown that specific FAs are able to cross the blood–brain barrier and participate in brain biochemical reactions (Ahmmed et al., 2020). Long-chain unsaturated FAs (*C* > 12) can activate G protein-coupled receptor 40 in the brain, ultimately altering emotional behavior and regulating the immune-inflammatory process (Kimura et al., 2020). Additionally, among the four unsaturated FAs identified in the study, 10-pentadecenoic acid (C15:1n-5c) was closely associated with several bacterial taxa, colonic glycerophospholipids, and SM (d40:1). These findings suggested that FAs may help determine the interaction between the gut microbiota and depression.

Numerous studies have observed significant microbiota composition alterations in various chronic stress-induced animal models of depression (Wu et al., 2020; Song et al., 2021). It has been reported that stress might directly alter the gut microbiota composition via enteric neuronal communication, increased colonic permeability and epithelial secretion, and the immune system (Westfall et al., 2017; Jiang et al., 2019). On the whole, at the phylum level, disturbances of *Firmicutes*, *Bacteroidetes*, and *Actinobacteria* were the hallmark in those investigations (Zheng et al., 2016; Wei et al., 2019). This is in line with our findings. In addition, we observed a lower abundance of the species of genus *Allobaculum* in the CSDS mice. A recent study also found that genus *Allobaculum* was decreased in mice subjected to chronic restrained stress, and its relative abundance was positively related to the fecal level of propionic acid (Wu et al., 2020). Of note, propionate has been reported to exert anti-depressant effects in rats exposed to CUMS (Li et al., 2018). Furtherly, oral treatment with probiotics *Lactobacillus reuteri* ameliorated mice depression-like behaviors induced by CSDS, and improved the decrease in abundance of *Allobaculum* (Xie R. et al., 2020). Moreover, a previous study aiming to explore the effects of the degree of lipid saturation on depression-like behavior showed that a fish oil-based diet could improve depression-like behaviors in mice and meanwhile elevate the abundance of *Allobaculum* (Lee et al., 2019). These findings suggested that *Allobaculum* restoration was closely associated with emotional improvement. In the study, we also found that *Allobaculum* spp. was significantly and negatively correlated with GABA, DIT, and alkanes, suggesting the species might be a potentially key bacteria that mediates the MGB axis.

Recent evidence has linked lipid metabolism disorder to the pathophysiology of depression (Xu et al., 2012), and showed that perturbation of lipids could increase the risk of suicide events (Zheng et al., 2013). Here, 36 differential lipids were identified; they belonged to glycerolipids, glycerophospholipids, and sphingolipids. More importantly, glycerophospholipids accounted for the most with 86.11%; among them, PC(16:0/20:4), PG(22:6/22:6), and PI(18:0/20:3) had significant correlations with anxiety- and depression-like behaviors to varying degrees. Glycerophospholipids, one of phospholipids, are critical components of neuronal membranes and myelin, and principal regulators of synaptic function (Romme et al., 2017; Lee et al., 2018). In line with the current findings, our previous study found that absence of gut microbiota could affect the metabolism of glycerophospholipids and sphingolipids in the prefrontal cortex of mice, and colonization with microbiota from specific pathogen-free mice to germ-free mice could restore part of these lipids (Chen et al., 2019). Meanwhile, we also found hippocampal glycerophospholipid metabolism disorder in depressive macaques, which was highly correlated with depression-like behaviors and several microbial genes, suggesting that gut microbiota may play a role in depression via glycerophospholipid metabolism (Zheng et al., 2020). Additionally, Liu X. et al. (2016) reported that phosphatidylcholine (PC), phosphatidylethanolamine (PE), phosphatidylinositol (PI), and sphingomyelin (SM) were remarkably increased in the plasma of depressed patients. Taken together, we speculated that glycerophospholipid metabolism disorder in the colon might be involved in depression, which might mediate the initial interactions between the microbiota and the host.

It should be mentioned there are some limitations in the present study: (i) Our study focused exclusively on lipidomic changes in the colon but did not consider brain regions associated with depression. Future research concerning key brain regions (e.g., hippocampus) is needed to better understand the role of the MGB axis; (ii) meanwhile, further research concerning other representative regions of large intestines, such as cecum, could extend our present study; (iii) due to limited resolution of the 16S rRNA sequencing method, further research based on shotgun metagenomics should be applied to identify certain bacterial species and microbial functions linked with depression; and (iv) the roles of fecal metabolites associated with differential bacterial species and colonic lipids require further intervention experiments to validate.

**FIGURE 6:**
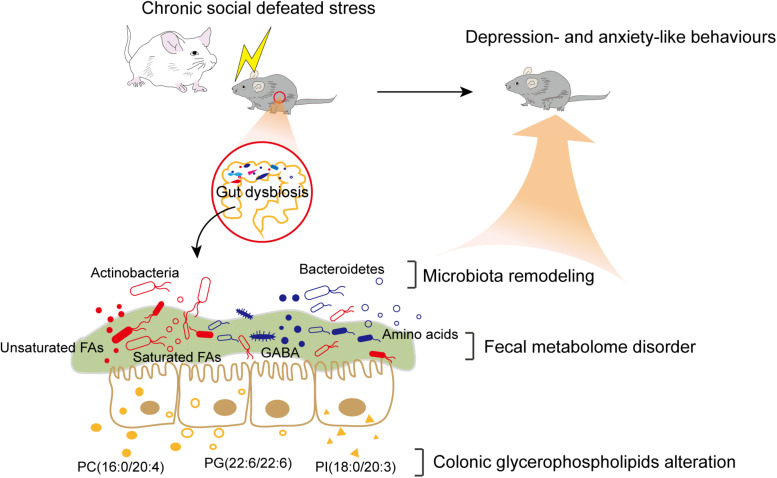
Brief summary of the study. CSDS induced gut microbiota remodeling and alterations of the fecal metabolome and colonic lipidome in C57BL/6J mice. The interactions among differential bacteria taxa, fecal metabolites, and colonic lipids might contribute to the potential mechanism of the MGB axis in the pathogenesis of depression.

In summary, our findings revealed that CSDS induced gut microbiota remodeling and alterations of the fecal metabolome and colonic lipidome (Figure 6). Several significant correlations between differential bacteria taxa, metabolites, and lipids were identified. Intriguingly, certain fecal metabolites were simultaneously associated with differential bacterial species and colonic lipids. Therefore, we speculated that altered colonic lipids, especially glycerophospholipids, might influence fecal metabolism, which reciprocally influence gut microbiota composition. Together, the interactions among microbiota, metabolites, and lipids create a highly integrated molecular communication network. These results highlighted the key role and potential mechanism of the MGB axis in the pathogenesis of depression and might provide useful information for future research.

## Data Availability Statement

The raw DNA sequence data were deposited in the National Center for NCBI Sequence Read Archive (https://www.ncbi.nlm.nih.gov/bioproject/PRJNA735389).

## Ethics Statement

The animal study was reviewed and approved by the Ethics Committee of Chongqing Medical University.

## Author Contributions

XG and PX contributed to the study concept and design. XG, CH, XY, and YH performed the experiments. JC and JP contributed to the experimental technical guidance. XG contributed to the data analysis and manuscript drafting. All authors reviewed and approved the manuscript before its submission.

## Conflict of Interest

The authors declare that the research was conducted in the absence of any commercial or financial relationships that could be construed as a potential conflict of interest.

## Footnotes

1 http://drive5.com/uparse/

2 http://rdp.cme.msu.edu/

## References

[B1] Ahmmed M. K. Ahmmed F. Tian H. S. Carne A. Bekhit A. E. (2020). Marine omega-3 (n-3) phospholipids: a comprehensive review of their properties, sources, bioavailability, and relation to brain health. *Compr. Rev. Food Sci. Food Saf*. 19 64–123. 10.1111/1541-4337.12510 33319514

[B2] Akkasheh G. Kashani-Poor Z. Tajabadi-Ebrahimi M. Jafari P. Akbari H. Taghizadeh M. (2016). Clinical and metabolic response to probiotic administration in patients with major depressive disorder: a randomized, double-blind, placebo-controlled trial. *Nutrition* 32 315–320. 10.1016/j.nut.2015.09.003 26706022

[B3] Boonstra E. de Kleijn R. Colzato L. S. Alkemade A. Forstmann B. U. Nieuwenhuis S. (2015). Neurotransmitters as food supplements: the effects of GABA on brain and behavior. *Front. Psychol*. 6:1520. 10.3389/fpsyg.2015.01520 26500584 PMC4594160

[B4] Burokas A. Arboleya S. Moloney R. D. Peterson V. L. Murphy K. Clarke G. (2017). Targeting the Microbiota-Gut-Brain Axis: prebiotics Have Anxiolytic and Antidepressant-like Effects and Reverse the Impact of Chronic Stress in Mice. *Biol. Psychiatry* 82 472–487. 10.1016/j.biopsych.2016.12.031 28242013

[B5] Chen H. Xie H. Huang S. Xiao T. Wang Z. Ni X. (2020). Development of mass spectrometry-based relatively quantitative targeted method for amino acids and neurotransmitters: applications in the diagnosis of major depression. *J. Pharm. Biomed. Anal.* 194:113773. 10.1016/j.jpba.2020.113773 33279298

[B6] Chen J.-J. He S. Fang L. Wang B. Bai S.-J. Xie J. (2020). Age-specific differential changes on gut microbiota composition in patients with major depressive disorder. *Aging* 12 2764–2776. 10.18632/aging.102775 32040443 PMC7041727

[B7] Chen J. J. Xie J. Zeng B. H. Li W. W. Bai S. J. Zhou C. (2019). Absence of gut microbiota affects lipid metabolism in the prefrontal cortex of mice. *Neurol. Res*. 41 1104–1112. 10.1080/01616412.2019.1675021 31587617

[B8] Cipriani A. Zhou X. Del Giovane C. Hetrick S. E. Qin B. Whittington C. (2016). Comparative efficacy and tolerability of antidepressants for major depressive disorder in children and adolescents: a network meta-analysis. *Lancet* 388 881–890. 10.1016/s0140-6736(16)30385-3 27289172

[B9] Duranti S. Ruiz L. Lugli G. A. Tames H. Milani C. Mancabelli L. (2020). Bifidobacterium adolescentis as a key member of the human gut microbiota in the production of GABA. *Sci. Rep*. 10:14112. 10.1038/s41598-020-70986-z 32839473 PMC7445748

[B10] Eckburg P. B. Bik E. M. Bernstein C. N. Purdom E. Dethlefsen L. Sargent M. (2005). Diversity of the human intestinal microbial flora. *Science* 308 1635–1638. 10.1126/science.1110591 15831718 PMC1395357

[B11] Gu S. Chen D. Zhang J. N. Lv X. Wang K. Duan L. P. (2013). Bacterial community mapping of the mouse gastrointestinal tract. *PLoS One*. 8:e74957. 10.1371/journal.pone.0074957 24116019 PMC3792069

[B12] Han T. L. Cannon R. D. Gallo S. M. Villas-Boas S. G. (2019). A metabolomic study of the effect of Candida albicans glutamate dehydrogenase deletion on growth and morphogenesis. *NPJ Biofilms Microbiomes* 5:13. 10.1038/s41522-019-0086-5 30992998 PMC6453907

[B13] Hu X. Yang J. Sun Y. Gao X. Zhang L. Li Y. (2018). Lanthanum chloride impairs memory in rats by disturbing the glutamate-glutamine cycle and over-activating NMDA receptors. *Food Chem. Toxicol.* 113 1–13. 10.1016/j.fct.2018.01.023 29353069

[B14] Hüls A. Robins C. Conneely K. N. De Jager P. L. Bennett D. A. Epstein M. P. (2020). Association between DNA methylation levels in brain tissue and late-life depression in community-based participants. *Transl. Psychiatry* 10:262. 10.1038/s41398-020-00948-6 32733030 PMC7393126

[B15] Jiang H. Ling Z. Zhang Y. Mao H. Ma Z. Yin Y. (2015). Altered fecal microbiota composition in patients with major depressive disorder. *Brain Behav. Immun.* 48 186–194. 10.1016/j.bbi.2015.03.016 25882912

[B16] Jiang Y. Greenwood-Van Meerveld B. Johnson A. C. Travagli R. A. (2019). Role of estrogen and stress on the brain-gut axis. *Am J. Physiol. Gastrointest. Liver Physiol*. 317 G203–G209. 10.1152/ajpgi.00144.2019 31241977 PMC6734369

[B17] Kagiyama T. Glushakov A. V. Sumners C. Roose B. Dennis D. M. Phillips M. I. (2004). Neuroprotective action of halogenated derivatives of L-phenylalanine. *Stroke* 35 1192–1196. 10.1161/01.Str.0000125722.10606.07 15073406

[B18] Kasper S. Cubała W. J. Fagiolini A. Ramos-Quiroga J. A. Souery D. Young A. H. (2020). Practical recommendations for the management of treatment-resistant depression with esketamine nasal spray therapy: basic science, evidence-based knowledge and expert guidance. *World J. Biol. Psychiatry.* 10.1080/15622975.2020.1836399 [Epub online ahead of print]. 33138665

[B19] Kimura I. Ichimura A. Ohue-Kitano R. Igarashi M. (2020). Free Fatty Acid Receptors in Health and Disease. *Physiol. Rev*. 100 171–210. 10.1152/physrev.00041.2018 31487233

[B20] Lee H. C. Lo Y. C. Yu S. C. Tung T. H. Lin I. H. Huang S. Y. (2019). Degree of lipid saturation affects depressive-like behaviour and gut microbiota in mice. *Int. J. Food Sci. Nutr.* 71 440–452. 10.1080/09637486.2019.1681380 31645150

[B21] Lee J. C. Park S. M. Kim I. Y. Sung H. Seong J. K. Moon M. H. (2018). High-fat diet-induced lipidome perturbations in the cortex, hippocampus, hypothalamus, and olfactory bulb of mice. *Biochim. Biophys. Acta Mol. Cell Biol. Lipids* 1863 980–990. 10.1016/j.bbalip.2018.05.007 29787912

[B22] Li J. Hou L. Wang C. Jia X. Qin X. Wu C. (2018). Short Term Intrarectal Administration of Sodium Propionate Induces Antidepressant-Like Effects in Rats Exposed to Chronic Unpredictable Mild Stress. *Front. Psychiatry* 9:454. 10.3389/fpsyt.2018.00454 30319461 PMC6170646

[B23] Li M. D’Arcy C. Meng X. (2016). Maltreatment in childhood substantially increases the risk of adult depression and anxiety in prospective cohort studies: systematic review, meta-analysis, and proportional attributable fractions. *Psychol. Med.* 46 717–730. 10.1017/s0033291715002743 26708271

[B24] Li N. Wang Q. Wang Y. Sun A. Lin Y. Jin Y. (2019). Fecal microbiota transplantation from chronic unpredictable mild stress mice donors affects anxiety-like and depression-like behavior in recipient mice via the gut microbiota-inflammation-brain axis. *Stress* 22 592–602. 10.1080/10253890.2019.1617267 31124390

[B25] Li Z. Y. Zheng X. Y. Gao X. X. Zhou Y. Z. Sun H. F. Zhang L. Z. (2010). Study of plasma metabolic profiling and biomarkers of chronic unpredictable mild stress rats based on gas chromatography/mass spectrometry. *Rapid Commun. Mass Spectrom.* 24 3539–3546. 10.1002/rcm.4809 21080506

[B26] Liu L. Zhou X. Zhang Y. Liu Y. Yang L. Pu J. (2016). The identification of metabolic disturbances in the prefrontal cortex of the chronic restraint stress rat model of depression. *Behav. Brain Res*. 305 148–156. 10.1016/j.bbr.2016.03.005 26947756

[B27] Liu S. Guo R. Liu F. Yuan Q. Yu Y. Ren F. (2020). Gut Microbiota Regulates Depression-Like Behavior in Rats Through the Neuroendocrine-Immune-Mitochondrial Pathway. *Neuropsychiatr. Dis. Treat*. 16 859–869. 10.2147/ndt.S243551 32280227 PMC7127849

[B28] Liu X. Li J. Zheng P. Zhao X. Zhou C. Hu C. (2016). Plasma lipidomics reveals potential lipid markers of major depressive disorder. *Anal. Bioanal. Chem*. 408 6497–6507. 10.1007/s00216-016-9768-5 27457104

[B29] Liu X. Liu C. Tian J. Gao X. Li K. Du G. (2020). Plasma metabolomics of depressed patients and treatment with Xiaoyaosan based on mass spectrometry technique. *J. Ethnopharmacol*. 246:112219. 10.1016/j.jep.2019.112219 31494201

[B30] Liu Y. Yieh L. Yang T. Drinkenburg W. Peeters P. Steckler T. (2016). Metabolomic biosignature differentiates melancholic depressive patients from healthy controls. *BMC Genomics* 17:669. 10.1186/s12864-016-2953-2 27549765 PMC4994306

[B31] Łoniewski I. Misera A. Skonieczna-Żydecka K. Kaczmarczyk M. Kaźmierczak-Siedlecka K. Misiak B. (2021). Major Depressive Disorder and gut microbiota - Association not causation. A scoping review. *Prog. Neuropsychopharmacol. Biol. Psychiatry* 106:110111. 10.1016/j.pnpbp.2020.110111 32976952

[B32] Lu J. Gong X. Yao X. Guang Y. Yang H. Ji R. (2021). Prolonged chronic social defeat stress promotes less resilience and higher uniformity in depression-like behaviors in adult male mice. *Biochem. Biophys. Res. Commun*. 553 107–113. 10.1016/j.bbrc.2021.03.058 33765554

[B33] Macia L. Tan J. Vieira A. T. Leach K. Stanley D. Luong S. (2015). Metabolite-sensing receptors GPR43 and GPR109A facilitate dietary fibre-induced gut homeostasis through regulation of the inflammasome. *Nat. Commun.* 6:6734. 10.1038/ncomms7734 25828455

[B34] Makris A. P. Karianaki M. Tsamis K. I. Paschou S. A. (2020). The role of the gut-brain axis in depression: endocrine, neural, and immune pathways. *Hormones* 20 1–12. 10.1007/s42000-020-00236-4 32827123

[B35] Marcondes Ávila P. R. Fiorot M. Michels M. Dominguini D. Abatti M. Vieira A. (2020). Effects of microbiota transplantation and the role of the vagus nerve in gut-brain axis in animals subjected to chronic mild stress. *J. Affect. Disord*. 277 410–416. 10.1016/j.jad.2020.08.013 32866799

[B36] Mazzoli R. Pessione E. (2016). The Neuro-endocrinological Role of Microbial Glutamate and GABA Signaling. *Front. Microbiol*. 7:1934. 10.3389/fmicb.2016.01934 27965654 PMC5127831

[B37] Paige L. A. Mitchell M. W. Krishnan K. R. Kaddurah-Daouk R. Steffens D. C. (2007). A preliminary metabolomic analysis of older adults with and without depression. *Int. J. Geriatr. Psychiatry* 22 418–423. 10.1002/gps.1690 17048218

[B38] Pan H. Q. Zhang W. H. Liao C. Z. He Y. Xiao Z. M. Qin X. (2020). Chronic Stress Oppositely Regulates Tonic Inhibition in Thy1-Expressing and Non-expressing Neurons in Amygdala. *Front. Neurosci*. 14:299. 10.3389/fnins.2020.00299 32362809 PMC7180173

[B39] Pu J. Liu Y. Zhang H. Tian L. Gui S. Yu Y. (2020). An integrated meta-analysis of peripheral blood metabolites and biological functions in major depressive disorder. *Mol. Psychiatry* 10.1038/s41380-020-0645-4 31959849 PMC8550972

[B40] Rivière A. Selak M. Lantin D. Leroy F. De Vuyst L. (2016). Bifidobacteria and Butyrate-Producing Colon Bacteria: importance and Strategies for Their Stimulation in the Human Gut. *Front. Microbiol.* 7:979. 10.3389/fmicb.2016.00979 27446020 PMC4923077

[B41] Romme I. A. de Reus M. A. Ophoff R. A. Kahn R. S. van den Heuvel M. P. (2017). Connectome Disconnectivity and Cortical Gene Expression in Patients With Schizophrenia. *Biol. Psychiatry* 81 495–502. 10.1016/j.biopsych.2016.07.012 27720199

[B42] Song X. Wang W. Ding S. Liu X. Wang Y. Ma H. (2021). Puerarin ameliorates depression-like behaviors of with chronic unpredictable mild stress mice by remodeling their gut microbiota. *J. Affect. Disord*. 290 353–363. 10.1016/j.jad.2021.04.037 34049088

[B43] Stilling R. M. van de Wouw M. Clarke G. Stanton C. Dinan T. G. Cryan J. F. (2016). The neuropharmacology of butyrate: the bread and butter of the microbiota-gut-brain axis? *Neurochem. Int*. 99 110–132. 10.1016/j.neuint.2016.06.011 27346602

[B44] Strandwitz P. Kim K. H. Terekhova D. Liu J. K. Sharma A. Levering J. (2019). GABA-modulating bacteria of the human gut microbiota. *Nat. Microbiol.* 4 396–403. 10.1038/s41564-018-0307-3 30531975 PMC6384127

[B45] Tian P. Wang G. Zhao J. Zhang H. Chen W. (2019). Bifidobacterium with the role of 5-hydroxytryptophan synthesis regulation alleviates the symptom of depression and related microbiota dysbiosis. *J. Nutr. Biochem*. 66 43–51. 10.1016/j.jnutbio.2019.01.007 30743155

[B46] Tian T. Xu B. Qin Y. Fan L. Chen J. Zheng P. (2019). Clostridium butyricum miyairi 588 has preventive effects on chronic social defeat stress-induced depressive-like behaviour and modulates microglial activation in mice. *Biochem. Biophys. Res. Commun*. 516 430–436. 10.1016/j.bbrc.2019.06.053 31227215

[B47] Tian Y. Wu Z. Wang Y. Chen C. He Y. Lan T. (2020). Alterations of neurotransmitters and related metabolites in the habenula from CUMS-susceptible and -resilient rats. *Biochem. Biophys. Res. Commun.* 534 422–428. 10.1016/j.bbrc.2020.11.065 33246560

[B48] Tillmann S. Abildgaard A. Winther G. Wegener G. (2019). Altered fecal microbiota composition in the Flinders sensitive line rat model of depression. *Psychopharmacology* 236 1445–1457. 10.1007/s00213-018-5094-2 30470860 PMC6599185

[B49] Voineskos D. Daskalakis Z. J. Blumberger D. M. (2020). Management of Treatment-Resistant Depression: challenges and Strategies. *Neuropsychiatr. Dis. Treat*. 16 221–234. 10.2147/ndt.S198774 32021216 PMC6982454

[B50] Wang W. Guo H. Zhang S. X. Li J. Cheng K. Bai S. J. (2016). Targeted Metabolomic Pathway Analysis and Validation Revealed Glutamatergic Disorder in the Prefrontal Cortex among the Chronic Social Defeat Stress Mice Model of Depression. *J. Proteome Res*. 15 3784–3792. 10.1021/acs.jproteome.6b00577 27599184

[B51] Warden D. Rush A. J. Trivedi M. H. Fava M. Wisniewski S. R. (2007). The STAR^∗^D Project results: a comprehensive review of findings. *Curr. Psychiatry Rep*. 9 449–459. 10.1007/s11920-007-0061-3 18221624

[B52] Wei L. Li Y. Tang W. Sun Q. Chen L. Wang X. (2019). Chronic Unpredictable Mild Stress in Rats Induces Colonic Inflammation. *Front. Physiol*. 10:1228. 10.3389/fphys.2019.01228 31616319 PMC6764080

[B53] Westfall S. Lomis N. Kahouli I. Dia S. Y. Singh S. P. Prakash S. (2017). Microbiome, probiotics and neurodegenerative diseases: deciphering the gut brain axis. *Cell. Mol. Life Sci.* 74 3769–3787. 10.1007/s00018-017-2550-9 28643167 PMC11107790

[B54] Wu M. Tian T. Mao Q. Zou T. Zhou C. J. Xie J. (2020). Associations between disordered gut microbiota and changes of neurotransmitters and short-chain fatty acids in depressed mice. *Transl. Psychiatry* 10:350. 10.1038/s41398-020-01038-3 33067412 PMC7567879

[B55] Wu Y. Tang J. Zhou C. Zhao L. Chen J. Zeng L. (2016). Quantitative proteomics analysis of the liver reveals immune regulation and lipid metabolism dysregulation in a mouse model of depression. *Behav. Brain Res*. 311 330–339. 10.1016/j.bbr.2016.05.057 27247144

[B56] Xie J. Han Y. Hong Y. Li W. W. Pei Q. Zhou X. (2020). Identification of Potential Metabolite Markers for Middle-Aged Patients with Post-Stroke Depression Using Urine Metabolomics. *Neuropsychiatr. Dis. Treat*. 16 2017–2024. 10.2147/ndt.S271990 32922015 PMC7457842

[B57] Xie R. Jiang P. Lin L. Jiang J. Yu B. Rao J. (2020). Oral treatment with Lactobacillus reuteri attenuates depressive-like behaviors and serotonin metabolism alterations induced by chronic social defeat stress. *J. Psychiatr. Res*. 122 70–78. 10.1016/j.jpsychires.2019.12.013 31927268

[B58] Xu H. B. Zhang R. F. Luo D. Zhou Y. Wang Y. Fang L. (2012). Comparative proteomic analysis of plasma from major depressive patients: identification of proteins associated with lipid metabolism and immunoregulation. *Int. J. Neuropsychopharmacol*. 15 1413–1425. 10.1017/s1461145712000302 22717272

[B59] Yang L.-N. Pu J.-C. Liu L.-X. Wang G.-W. Zhou X.-Y. Zhang Y.-Q. (2019). Integrated Metabolomics and Proteomics Analysis Revealed Second Messenger System Disturbance in Hippocampus of Chronic Social Defeat Stress Rat. *Front. Neurosci*. 13:247. 10.3389/fnins.2019.00247 30983951 PMC6448023

[B60] Yang Y. Yin Y. Chen X. Chen C. Xia Y. Qi H. (2019). Evaluating different extraction solvents for GC-MS based metabolomic analysis of the fecal metabolome of adult and baby giant pandas. *Sci. Rep*. 9:12017. 10.1038/s41598-019-48453-1 31427618 PMC6700143

[B61] Yu M. Jia H. Zhou C. Yang Y. Zhao Y. Yang M. (2017). Variations in gut microbiota and fecal metabolic phenotype associated with depression by 16S rRNA gene sequencing and LC/MS-based metabolomics. *J. Pharm. Biomed. Anal*. 138 231–239. 10.1016/j.jpba.2017.02.008 28219800

[B62] Zhao J. Verwer R. W. H. Gao S. F. Qi X. R. Lucassen P. J. Kessels H. W. (2018). Prefrontal alterations in GABAergic and glutamatergic gene expression in relation to depression and suicide. *J. Psychiatr. Res*. 102 261–274. 10.1016/j.jpsychires.2018.04.020 29753198

[B63] Zheng P. Gao H. C. Qi Z. G. Jia J. M. Li F. F. Chen J. J. (2013). Peripheral metabolic abnormalities of lipids and amino acids implicated in increased risk of suicidal behavior in major depressive disorder. *Metabolomics* 9 688–696. 10.1007/s11306-012-0474-9

[B64] Zheng P. Wu J. Zhang H. Perry S. W. Yin B. Tan X. (2020). The gut microbiome modulates gut-brain axis glycerophospholipid metabolism in a region-specific manner in a nonhuman primate model of depression. *Mol. Psychiatry* 10.1038/s41380-020-0744-2 [Epub Online ahead of print]. 32376998 PMC8440210

[B65] Zheng P. Zeng B. Liu M. Chen J. Pan J. Han Y. (2019). The gut microbiome from patients with schizophrenia modulates the glutamate-glutamine-GABA cycle and schizophrenia-relevant behaviors in mice. *Sci. Adv*. 5:eaau8317. 10.1126/sciadv.aau8317 30775438 PMC6365110

[B66] Zheng P. Zeng B. Zhou C. Liu M. Fang Z. Xu X. (2016). Gut microbiome remodeling induces depressive-like behaviors through a pathway mediated by the host’s metabolism. *Mol. Psychiatry* 21 786–796. 10.1038/mp.2016.44 27067014

