# Supplementary material for: Altered Fecal Metabolites and Colonic Glycerophospholipids Were Associated With Abnormal Composition of Gut Microbiota in a Depression Model of Mice
Source: Front Neurosci. 2021 Jul 19;15:701355. doi: 10.3389/fnins.2021.701355 (PMC8326978; doi:10.3389/fnins.2021.701355)
Supplement: Supplementary file 1 [file Data_Sheet_1.pdf]

Supplementary Table S1. The taxonomy annotations of identified gut microbial OTU in the control and CSDS mice.

OTU	Phylum	Class	Order	Family	Genus	Species
OTU700	p__Firmicutes	c__Clostridia	o__Clostridiales	f__Lachnospira	g__Lachnospiraceae_NK4A136_group	s__unclassified_g__Lachnospiraceae_NK4A136_group
OTU345	p__Firmicutes	c__Clostridia	o__Clostridiales	f__Clostridiace	g__Clostridium_sensu_stricto_1	s__Clostridium_sp._ND2
OTU622	p__Firmicutes	c__Clostridia	o__Clostridiales	f__Lachnospira	g__unclassified_f__Lachnospiraceae	s__unclassified_f__Lachnospiraceae
OTU185	p__Firmicutes	c__Clostridia	o__Clostridiales	f__Clostridiales	g__norank_f__Clostridiales_vadinBB60_group	s__unclassified_g__norank_f__Clostridiales_vadinBB60_grou
OTU682	p__Firmicutes	c__Clostridia	o__Clostridiales	f__Lachnospira	g__unclassified_f__Lachnospiraceae	s__unclassified_f__Lachnospiraceae
OTU685	p__Firmicutes	c__Clostridia	o__Clostridiales	f__Lachnospira	g__unclassified_f__Lachnospiraceae	s__unclassified_f__Lachnospiraceae
OTU423	p__Spirochaeta	c__Spirochaete	o__Spirochaetale	f__Brachyspira	g__Brachyspira	s__unclassified_g__Brachyspira
OTU263	p__Firmicutes	c__Clostridia	o__Clostridiales	f__Ruminococc	g__Ruminococcaceae_UCG-014	s__uncultured_organism_g__Ruminococcaceae_UCG-014
OTU99	p__Firmicutes	c__Clostridia	o__Clostridiales	f__Lachnospira	g__[Ruminococcus]_torques_group	s__uncultured_bacterium_g__[Ruminococcus]_torques_group
OTU525	p__Firmicutes	c__Clostridia	o__Clostridiales	f__Clostridiales	g__norank_f__Clostridiales_vadinBB60_group	s__unclassified_g__norank_f__Clostridiales_vadinBB60_grou
OTU418	p__Firmicutes	c__Clostridia	o__Clostridiales	f__Lachnospira	g__Roseburia	s__Lachnospiraceae_bacterium_DW52
OTU100	p__Firmicutes	c__Clostridia	o__Clostridiales	f__Ruminococc	g__Ruminococcaceae_UCG-014	s__uncultured_bacterium_g__Ruminococcaceae_UCG-014
OTU210	p__Firmicutes	c__Clostridia	o__Clostridiales	f__Clostridiales	g__norank_f__Clostridiales_vadinBB60_group	s__uncultured_bacterium_g__norank_f__Clostridiales_vadinI
OTU80	p__Firmicutes	c__Clostridia	o__Clostridiales	f__Ruminococc	g__Ruminococcaceae_UCG-013	s__unclassified_g__Ruminococcaceae_UCG-013
OTU654	p__Bacteroidet	c__Bacteroidia	o__Bacteroidales	f__Prevotellace	g__unclassified_f__Prevotellaceae	s__unclassified_f__Prevotellaceae
OTU325	p__Firmicutes	c__Negativicut	o__Selenomonad	f__Veillonellac	g__Quinella	s__uncultured_bacterium_g__Quinella
OTU158	p__Firmicutes	c__Clostridia	o__Clostridiales	f__Clostridiales	g__norank_f__Clostridiales_vadinBB60_group	s__unclassified_g__norank_f__Clostridiales_vadinBB60_grou
OTU483	p__Firmicutes	c__Clostridia	o__Clostridiales	f__Lachnospira	g__Lachnospiraceae_NK4A136_group	s__unclassified_g__Lachnospiraceae_NK4A136_group
OTU131	p__Firmicutes	c__Clostridia	o__Clostridiales	f__Lachnospira	g__Lachnospiraceae_UCG-001	s__unclassified_g__Lachnospiraceae_UCG-001
OTU456	p__Firmicutes	c__Clostridia	o__Clostridiales	f__Lachnospira	g__Lachnospiraceae_NK4A136_group	s__uncultured_bacterium_g__Lachnospiraceae_NK4A136_gr
OTU698	p__Firmicutes	c__Clostridia	o__Clostridiales	f__Ruminococc	g__Anaerotruncus	s__uncultured_bacterium_g__Anaerotruncus
OTU291	p__Firmicutes	c__Clostridia	o__Clostridiales	f__Family_XIII	g__Family_XIII_AD3011_group	s__unclassified_g__Family_XIII_AD3011_group
OTU390	p__Firmicutes	c__Clostridia	o__Clostridiales	f__Peptococcac	g__norank_f__Peptococcaceae	s__unclassified_g__norank_f__Peptococcaceae
OTU706	p__Proteobacte	c__Deltaproteo	o__Desulfovibric	f__Desulfovibri	g__Bilophila	s__uncultured_bacterium_g__Bilophila
OTU498	p__Bacteroidet	c__Bacteroidia	o__Bacteroidales	f__Prevotellace	g__Prevotellaceae_UCG-003	s__unclassified_g__Prevotellaceae_UCG-003
OTU71	p__Firmicutes	c__Clostridia	o__Clostridiales	f__Ruminococc	g__unclassified_f__Ruminococcaceae	s__unclassified_f__Ruminococcaceae
OTU350	p__Firmicutes	c__Bacilli	o__Lactobacillal	f__Lactobacilla	g__Lactobacillus	s__unclassified_g__Lactobacillus
OTU246	p__Firmicutes	c__Clostridia	o__Clostridiales	f__Lachnospira	g__Roseburia	s__unclassified_g__Roseburia
OTU385	p__Tenericutes	c__Mollicutes	o__Mollicutes_R	f__norank_o__	g__norank_o__Mollicutes_RF9	s__unclassified_g__norank_o__Mollicutes_RF9
OTU77	p__Firmicutes	c__Clostridia	o__Clostridiales	f__Ruminococc	g__unclassified_f__Ruminococcaceae	s__unclassified_f__Ruminococcaceae
OTU712	p__Bacteroidet	c__Bacteroidia	o__Bacteroidales	f__Bacteroidale	g__norank_f__Bacteroidales_S24-7_group	s__unclassified_g__norank_f__Bacteroidales_S24-7_group
OTU274	p__Deferribact	c__Deferribact	o__Deferribacter	f__Deferribacte	g__Mucispirillum	s__unclassified_g__Mucispirillum
OTU553	p__Tenericutes	c__Mollicutes	o__Mollicutes_R	f__norank_o__	g__norank_o__Mollicutes_RF9	s__uncultured_bacterium_g__norank_o__Mollicutes_RF9
OTU384	p__Firmicutes	c__Clostridia	o__Clostridiales	f__Lachnospira	g__Lachnospiraceae_NK4A136_group	s__unclassified_g__Lachnospiraceae_NK4A136_group
OTU136	p__Firmicutes	c__Clostridia	o__Clostridiales	f__Lachnospira	g__norank_f__Lachnospiraceae	s__unclassified_g__norank_f__Lachnospiraceae
OTU30	p__Bacteroidet	c__Bacteroidia	o__Bacteroidales	f__Rikenellace	g__Rikenellaceae_RC9_gut_group	s__unclassified_g__Rikenellaceae_RC9_gut_group

OTU126	p__Firmicutes	c__Clostridia	o__Clostridiales	f__Ruminococc	g__[Eubacterium]_coprostanoligenes_group		s__gut_metagenome_g__[Eubacterium]_coprostanoligenes_g
OTU162	p__Firmicutes	c__Clostridia	o__Clostridiales	f__Ruminococc	g__Caproiciproducens		s__unclassified_g__Caproiciproducens
OTU702	p__Firmicutes	c__Clostridia	o__Clostridiales	f__Ruminococc	g__Ruminiclostridium_5		s__unclassified_g__Ruminiclostridium_5
OTU221	p__Firmicutes	c__Erysipelotri	o__Erysipelotricl	f__Erysipelotric	g__Holdemanella		s__uncultured_bacterium_g__Holdemanella
OTU715	p__Bacteroidet	c__Bacteroidia	o__Bacteroidales	f__Bacteroidale	g__norank_f__Bacteroidales_S24-7_group		s__unclassified_g__norank_f__Bacteroidales_S24-7_group
OTU271	p__Firmicutes	c__Clostridia	o__Clostridiales	f__Lachnospira	g__[Eubacterium]_ventriosum_group		s__uncultured_organism_g__[Eubacterium]_ventriosum_grou
OTU294	p__Firmicutes	c__Clostridia	o__Clostridiales	f__Ruminococc	g__unclassified_f__Ruminococcaceae		s__unclassified_f__Ruminococcaceae
OTU664	p__Firmicutes	c__Clostridia	o__Clostridiales	f__Lachnospira	g__unclassified_f__Lachnospiraceae		s__unclassified_f__Lachnospiraceae
OTU548	p__Bacteroidet	c__Bacteroidia	o__Bacteroidales	f__Porphyromo	g__Odoribacter		s__uncultured_bacterium_g__Odoribacter
OTU632	p__Firmicutes	c__Clostridia	o__Clostridiales	f__Ruminococc	g__Anaerotruncus		s__unclassified_g__Anaerotruncus
OTU431	p__Bacteroidet	c__Bacteroidia	o__Bacteroidales	f__Bacteroidale	g__norank_f__Bacteroidales_S24-7_group		s__uncultured_bacterium_g__norank_f__Bacteroidales_S24-7
OTU213	p__Bacteroidet	c__Bacteroidia	o__Bacteroidales	f__Prevotellace	g__Prevotellaceae_UCG-001		s__uncultured_Bacteroidales_bacterium_g__Prevotellaceae_U
OTU64	p__Firmicutes	c__Clostridia	o__Clostridiales	f__Lachnospira	g__Roseburia		s__unclassified_g__Roseburia
OTU663	p__Firmicutes	c__Clostridia	o__Clostridiales	f__Lachnospira	g__unclassified_f__Lachnospiraceae		s__unclassified_f__Lachnospiraceae
OTU349	p__Bacteroidet	c__Bacteroidia	o__Bacteroidales	f__Bacteroidac	g__Bacteroides		s__Bacteroides_vulgatus_ATCC_8482
OTU261	p__unclassified	c__unclassified	o__unclassified_	f__unclassified_	g__unclassified_k__norank		s__unclassified_k__norank
OTU307	p__Actinobacte	c__Actinobacte	o__Coriobacteria	f__Coriobacteri	g__Adlercreutzia		s__uncultured_bacterium_g__Adlercreutzia
OTU54	p__Bacteroidet	c__Bacteroidia	o__Bacteroidales	f__Bacteroidale	g__norank_f__Bacteroidales_S24-7_group		s__uncultured_bacterium_g__norank_f__Bacteroidales_S24-7
OTU703	p__Firmicutes	c__Clostridia	o__Clostridiales	f__Lachnospira	g__Lachnospiraceae_NK4A136_group		s__unclassified_g__Lachnospiraceae_NK4A136_group
OTU72	p__Firmicutes	c__Clostridia	o__Clostridiales	f__Ruminococc	g__norank_f__Ruminococcaceae		s__[Clostridium]_leptum_g__norank
OTU91	p__Firmicutes	c__Clostridia	o__Clostridiales	f__Clostridiales	g__norank_f__Clostridiales_vadinBB60_group		s__unclassified_g__norank_f__Clostridiales_vadinBB60_grou
OTU537	p__Bacteroidet	c__Bacteroidia	o__Bacteroidales	f__Bacteroidale	g__norank_f__Bacteroidales_S24-7_group		s__uncultured_bacterium_g__norank_f__Bacteroidales_S24-7
OTU48	p__Bacteroidet	c__Bacteroidia	o__Bacteroidales	f__unclassified_	g__unclassified_o__Bacteroidales		s__unclassified_o__Bacteroidales
OTU555	p__Firmicutes	c__Clostridia	o__Clostridiales	f__Lachnospira	g__norank_f__Lachnospiraceae		s__unclassified_g__norank_f__Lachnospiraceae
OTU62	p__Firmicutes	c__Clostridia	o__Clostridiales	f__Lachnospira	g__Roseburia		s__unclassified_g__Roseburia
OTU354	p__Firmicutes	c__Clostridia	o__Clostridiales	f__Lachnospira	g__Lachnospiraceae_NK4A136_group		s__uncultured_bacterium_g__Lachnospiraceae_NK4A136_gr
OTU656	p__Proteobacte	c__Alphaprote	o__Rhodospirilla	f__Rhodospirill	g__norank_f__Rhodospirillaceae		s__gut_metagenome_g__norank_f__Rhodospirillaceae
OTU270	p__Bacteroidet	c__Bacteroidia	o__Bacteroidales	f__Bacteroidale	g__norank_f__Bacteroidales_S24-7_group		s__uncultured_Bacteroidales_bacterium_g__norank_f__Bacte
OTU135	p__Firmicutes	c__Clostridia	o__Clostridiales	f__Lachnospira	g__unclassified_f__Lachnospiraceae		s__unclassified_f__Lachnospiraceae
OTU550	p__Firmicutes	c__Erysipelotri	o__Erysipelotricl	f__Erysipelotric	g__Candidatus_Stoquefichus		s__uncultured_bacterium_g__Candidatus_Stoquefichus
OTU53	p__Bacteroidet	c__Bacteroidia	o__Bacteroidales	f__Bacteroidale	g__norank_f__Bacteroidales_S24-7_group		s__uncultured_bacterium_g__norank_f__Bacteroidales_S24-7
OTU683	p__Firmicutes	c__Clostridia	o__Clostridiales	f__Ruminococc	g__Ruminococcaceae_UCG-014		s__unclassified_g__Ruminococcaceae_UCG-014
OTU281	p__Proteobacte	c__Deltaproteo	o__Desulfovibric	f__Desulfovibri	g__Desulfovibrio		s__uncultured_Desulfovibrionales_bacterium_g__Desulfovibı
OTU541	p__Firmicutes	c__Clostridia	o__Clostridiales	f__Ruminococc	g__Ruminococcaceae_UCG-014		s__unclassified_g__Ruminococcaceae_UCG-014
OTU21	p__Firmicutes	c__Clostridia	o__Clostridiales	f__Ruminococc	g__Ruminococcaceae_UCG-010		s__unclassified_g__Ruminococcaceae_UCG-010
OTU627	p__Bacteroidet	c__Bacteroidia	o__Bacteroidales	f__Bacteroidale	g__norank_f__Bacteroidales_S24-7_group		s__uncultured_bacterium_g__norank_f__Bacteroidales_S24-7
OTU164	p__Firmicutes	c__Clostridia	o__Clostridiales	f__Ruminococc	g__Ruminiclostridium_9		s__unclassified_g__Ruminiclostridium_9
OTU31	p__Firmicutes	c__Clostridia	o__Clostridiales	f__Family_XIII	g__[Eubacterium]_brachy_group		s__uncultured_Eubacteriaceae_bacterium_g__[Eubacterium]_
OTU549	p__Bacteroidet	c__Bacteroidia	o__Bacteroidales	f__Bacteroidale	g__norank_f__Bacteroidales_S24-7_group		s__uncultured_bacterium_g__norank_f__Bacteroidales_S24-7

OTU329	p__Bacteroidet	c__Bacteroidia	o__Bacteroidales	f__Porphyromo	g__Odoribacter	s__unclassified_g__Odoribacter
OTU293	p__Actinobacte	c__Actinobacte	o__Coriobacteria	f__Coriobacteri	g__norank_f__Coriobacteriaceae	s__uncultured_Coriobacteriales_bacterium
OTU369	p__Firmicutes	c__Clostridia	o__Clostridiales	f__Lachnospira	g__Lachnospiraceae_NK4A136_group	s__unclassified_g__Lachnospiraceae_NK4A136_group
OTU298	p__Firmicutes	c__Clostridia	o__Clostridiales	f__Lachnospira	g__Lachnospiraceae_NK4A136_group	s__unclassified_g__Lachnospiraceae_NK4A136_group
OTU436	p__Firmicutes	c__Clostridia	o__Clostridiales	f__Ruminococc	g__Oscillibacter	s__uncultured_bacterium_g__Oscillibacter
OTU132	p__Firmicutes	c__Clostridia	o__Clostridiales	f__Lachnospira	g__Roseburia	s__unclassified_g__Roseburia
OTU434	p__Firmicutes	c__Clostridia	o__Clostridiales	f__Ruminococc	g__Anaerotruncus	s__Anaerotruncus_colihominis_DSM_17241
OTU399	p__Firmicutes	c__Clostridia	o__Clostridiales	f__Family_XIII	g__[Eubacterium]_brachy_group	s__uncultured_bacterium_g__[Eubacterium]_brachy_group
OTU95	p__Firmicutes	c__Clostridia	o__Clostridiales	f__Clostridiales	g__norank_f__Clostridiales_vadinBB60_group	s__uncultured_bacterium_g__norank_f__Clostridiales_vadinI
OTU397	p__Firmicutes	c__Bacilli	o__Lactobacillal	f__Lactobacilla	g__Lactobacillus	s__unclassified_g__Lactobacillus
OTU426	p__Firmicutes	c__Clostridia	o__Clostridiales	f__Peptococcac	g__Peptococcus	s__uncultured_bacterium_g__Peptococcus
OTU165	p__Tenericutes	c__Mollicutes	o__Mollicutes_R	f__norank_o__	g__norank_o__Mollicutes_RF9	s__unclassified_g__norank_o__Mollicutes_RF9
OTU716	p__Firmicutes	c__Clostridia	o__Clostridiales	f__Lachnospira	g__Marvinbryantia	s__Clostridiales_bacterium_CIEAF_012
OTU217	p__Firmicutes	c__Clostridia	o__Clostridiales	f__Lachnospira	g__Blautia	s__Ruminococcus_sp._5_1_39BFAA
OTU489	p__Bacteroidet	c__Bacteroidia	o__Bacteroidales	f__Rikenellacea	g__Rikenella	s__unclassified_g__Rikenella
OTU36	p__Bacteroidet	c__Bacteroidia	o__Bacteroidales	f__Bacteroidale	g__norank_f__Bacteroidales_S24-7_group	s__uncultured_bacterium_g__norank_f__Bacteroidales_S24-7
OTU127	p__Firmicutes	c__Clostridia	o__Clostridiales	f__Lachnospira	g__Roseburia	s__unclassified_g__Roseburia
OTU4	p__Firmicutes	c__Clostridia	o__Clostridiales	f__Ruminococc	g__Ruminiclostridium_5	s__uncultured_Clostridiales_bacterium_g__Ruminiclostridiun
OTU129	p__Firmicutes	c__Clostridia	o__Clostridiales	f__Lachnospira	g__Lachnospiraceae_UCG-001	s__unclassified_g__Lachnospiraceae_UCG-001
OTU303	p__Bacteroidet	c__Bacteroidia	o__Bacteroidales	f__Prevotellace	g__Prevotellaceae_UCG-001	s__unclassified_g__Prevotellaceae_UCG-001
OTU49	p__Bacteroidet	c__Bacteroidia	o__Bacteroidales	f__Bacteroidale	g__norank_f__Bacteroidales_S24-7_group	s__unclassified_g__norank_f__Bacteroidales_S24-7_group
OTU351	p__Firmicutes	c__unclassified	o__unclassified_	f__unclassified_	g__unclassified_p__Firmicutes	s__unclassified_p__Firmicutes
OTU573	p__Firmicutes	c__Clostridia	o__Clostridiales	f__Lachnospira	g__norank_f__Lachnospiraceae	s__uncultured_bacterium_g__norank_f__Lachnospiraceae
OTU358	p__Bacteroidet	c__Bacteroidia	o__Bacteroidales	f__Bacteroidale	g__norank_f__Bacteroidales_S24-7_group	s__uncultured_bacterium_g__norank_f__Bacteroidales_S24-7
OTU471	p__Firmicutes	c__Clostridia	o__Clostridiales	f__Lachnospira	g__norank_f__Lachnospiraceae	s__unclassified_g__norank_f__Lachnospiraceae
OTU333	p__Firmicutes	c__Clostridia	o__Clostridiales	f__Ruminococc	g__unclassified_f__Ruminococcaceae	s__unclassified_f__Ruminococcaceae
OTU613	p__Firmicutes	c__Erysipelotri	o__Erysipelotricl	f__Erysipelotric	g__unclassified_f__Erysipelotrichaceae	s__unclassified_f__Erysipelotrichaceae
OTU331	p__Firmicutes	c__Clostridia	o__Clostridiales	f__Lachnospira	g__Lachnospiraceae_NK4A136_group	s__uncultured_bacterium_g__Lachnospiraceae_NK4A136_gr
OTU558	p__Bacteroidet	c__Bacteroidia	o__Bacteroidales	f__Bacteroidale	g__norank_f__Bacteroidales_S24-7_group	s__uncultured_bacterium_g__norank_f__Bacteroidales_S24-7
OTU598	p__Firmicutes	c__Erysipelotri	o__Erysipelotricl	f__Erysipelotric	g__unclassified_f__Erysipelotrichaceae	s__unclassified_f__Erysipelotrichaceae
OTU398	p__Firmicutes	c__Clostridia	o__Clostridiales	f__Lachnospira	g__Lachnospiraceae_FCS020_group	s__unclassified_g__Lachnospiraceae_FCS020_group
OTU28	p__Firmicutes	c__Clostridia	o__Clostridiales	f__Ruminococc	g__Ruminiclostridium_6	s__unclassified_g__Ruminiclostridium_6
OTU446	p__Firmicutes	c__Clostridia	o__Clostridiales	f__Lachnospira	g__Lachnospiraceae_NK4A136_group	s__uncultured_bacterium_g__Lachnospiraceae_NK4A136_gr
OTU648	p__Firmicutes	c__Clostridia	o__Clostridiales	f__Peptococcac	g__norank_f__Peptococcaceae	s__unclassified_g__norank_f__Peptococcaceae
OTU29	p__Firmicutes	c__Clostridia	o__Clostridiales	f__Lachnospira	g__Lachnospiraceae_NC2004_group	s__unclassified_g__Lachnospiraceae_NC2004_group
OTU687	p__Firmicutes	c__Clostridia	o__Clostridiales	f__Lachnospira	g__Marvinbryantia	s__uncultured_bacterium_g__Marvinbryantia
OTU717	p__Firmicutes	c__Clostridia	o__Clostridiales	f__Lachnospira	g__Roseburia	s__uncultured_bacterium_g__Roseburia
OTU82	p__Actinobacte	c__Actinobacte	o__Coriobacteria	f__Coriobacteri	g__unclassified_f__Coriobacteriaceae	s__unclassified_f__Coriobacteriaceae
OTU497	p__Firmicutes	c__Clostridia	o__Clostridiales	f__Lachnospira	g__unclassified_f__Lachnospiraceae	s__unclassified_f__Lachnospiraceae

OTU355	p__Bacteroidet	c__Bacteroidia	o__Bacteroidales	f__Prevotellace	g__Prevotellaceae_UCG-001	s__unclassified_g__Prevotellaceae_UCG-001
OTU381	p__Firmicutes	c__Clostridia	o__Clostridiales	f__Clostridiales	g__norank_f__Clostridiales_vadinBB60_group	s__uncultured_bacterium_g__norank_f__Clostridiales_vadinI
OTU20	p__Firmicutes	c__Clostridia	o__Clostridiales	f__Ruminococc	g__Ruminococcaceae_UCG-014	s__uncultured_bacterium_g__Ruminococcaceae_UCG-014
OTU533	p__Firmicutes	c__Clostridia	o__Clostridiales	f__Lachnospira	g__Lachnospiraceae_NK4A136_group	s__unclassified_g__Lachnospiraceae_NK4A136_group
OTU401	p__Firmicutes	c__unclassified	o__unclassified_	f__unclassified_	g__unclassified_p__Firmicutes	s__unclassified_p__Firmicutes
OTU634	p__Proteobacte	c__Alphaprote	o__Sphingomona	f__Sphingomon	g__Sphingomonas	s__Sphingomonas_paucimobilis_g__Sphingomonas
OTU78	p__Firmicutes	c__Clostridia	o__Clostridiales	f__Lachnospira	g__Lachnospiraceae_NK4A136_group	s__unclassified_g__Lachnospiraceae_NK4A136_group
OTU283	p__Firmicutes	c__Clostridia	o__Clostridiales	f__Lachnospira	g__norank_f__Lachnospiraceae	s__Clostridium_sp._Culture-54
OTU370	p__Actinobacte	c__Actinobacte	o__Coriobacteria	f__Coriobacteri	g__Enterorhabdus	s__uncultured_bacterium_g__Enterorhabdus
OTU377	p__Firmicutes	c__Clostridia	o__Clostridiales	f__Ruminococc	g__unclassified_f__Ruminococcaceae	s__unclassified_f__Ruminococcaceae
OTU284	p__Proteobacte	c__Deltaproteo	o__Desulfovibric	f__Desulfovibri	g__Desulfovibrio	s__unclassified_g__Desulfovibrio
OTU79	p__Bacteroidet	c__Bacteroidia	o__Bacteroidales	f__Bacteroidale	g__norank_f__Bacteroidales_S24-7_group	s__uncultured_bacterium_g__norank_f__Bacteroidales_S24-7
OTU194	p__Bacteroidet	c__Bacteroidia	o__Bacteroidales	f__Bacteroidale	g__norank_f__Bacteroidales_S24-7_group	s__uncultured_Bacteroidales_bacterium_g__norank_f__Bacte
OTU538	p__Firmicutes	c__Clostridia	o__Clostridiales	f__Lachnospira	g__unclassified_f__Lachnospiraceae	s__unclassified_f__Lachnospiraceae
OTU203	p__Bacteroidet	c__Bacteroidia	o__Bacteroidales	f__Bacteroidale	g__norank_f__Bacteroidales_S24-7_group	s__uncultured_bacterium_g__norank_f__Bacteroidales_S24-7
OTU57	p__Bacteroidet	c__Bacteroidia	o__Bacteroidales	f__Bacteroidale	g__norank_f__Bacteroidales_S24-7_group	s__uncultured_organism_g__norank_f__Bacteroidales_S24-7
OTU578	p__Firmicutes	c__Clostridia	o__Clostridiales	f__Christensene	g__norank_f__Christensenellaceae	s__uncultured_bacterium_g__norank_f__Christensenellaceae
OTU611	p__Firmicutes	c__Clostridia	o__Clostridiales	f__Ruminococc	g__Ruminococcaceae_UCG-005	s__uncultured_Clostridiales_bacterium_g__Ruminococcaceae
OTU500	p__Bacteroidet	c__Bacteroidia	o__Bacteroidales	f__Bacteroidac	g__Bacteroides	s__unclassified_g__Bacteroides
OTU605	p__Firmicutes	c__Clostridia	o__Clostridiales	f__Lachnospira	g__Lachnospiraceae_UCG-001	s__unclassified_g__Lachnospiraceae_UCG-001
OTU220	p__Bacteroidet	c__Bacteroidia	o__Bacteroidales	f__Bacteroidale	g__norank_f__Bacteroidales_S24-7_group	s__uncultured_Bacteroidales_bacterium_g__norank_f__Bacte
OTU499	p__Bacteroidet	c__Bacteroidia	o__Bacteroidales	f__unclassified_	g__unclassified_o__Bacteroidales	s__unclassified_o__Bacteroidales
OTU474	p__Firmicutes	c__Clostridia	o__Clostridiales	f__Lachnospira	g__unclassified_f__Lachnospiraceae	s__unclassified_f__Lachnospiraceae
OTU309	p__Bacteroidet	c__Bacteroidia	o__Bacteroidales	f__Bacteroidale	g__norank_f__Bacteroidales_S24-7_group	s__unclassified_g__norank_f__Bacteroidales_S24-7_group
OTU459	p__Firmicutes	c__Clostridia	o__Clostridiales	f__Ruminococc	g__unclassified_f__Ruminococcaceae	s__unclassified_f__Ruminococcaceae
OTU526	p__Bacteroidet	c__Bacteroidia	o__Bacteroidales	f__Prevotellace	g__Prevotellaceae_UCG-001	s__uncultured_Bacteroidales_bacterium_g__Prevotellaceae_U
OTU403	p__Firmicutes	c__Clostridia	o__Clostridiales	f__Ruminococc	g__Butyricicoccus	s__unclassified_g__Butyricicoccus
OTU175	p__Bacteroidet	c__Bacteroidia	o__Bacteroidales	f__Bacteroidale	g__norank_f__Bacteroidales_S24-7_group	s__unclassified_g__norank_f__Bacteroidales_S24-7_group
OTU625	p__Firmicutes	c__Erysipelotri	o__Erysipelotricl	f__Erysipelotric	g__unclassified_f__Erysipelotrichaceae	s__unclassified_f__Erysipelotrichaceae
OTU612	p__Firmicutes	c__Clostridia	o__Clostridiales	f__Clostridiales	g__norank_f__Clostridiales_vadinBB60_group	s__unclassified_g__norank_f__Clostridiales_vadinBB60_grou
OTU156	p__Firmicutes	c__Bacilli	o__Lactobacillal	f__Lactobacilla	g__Lactobacillus	s__unclassified_g__Lactobacillus
OTU159	p__Firmicutes	c__Bacilli	o__Lactobacillal	f__Lactobacilla	g__Lactobacillus	s__unclassified_g__Lactobacillus
OTU26	p__Firmicutes	c__Clostridia	o__Clostridiales	f__Lachnospira	g__unclassified_f__Lachnospiraceae	s__unclassified_f__Lachnospiraceae
OTU308	p__Proteobacte	c__Deltaproteo	o__Desulfovibric	f__Desulfovibri	g__Desulfovibrio	s__uncultured_bacterium_g__Desulfovibrio
OTU691	p__Bacteroidet	c__Bacteroidia	o__Bacteroidales	f__Prevotellace	g__unclassified_f__Prevotellaceae	s__unclassified_f__Prevotellaceae
OTU496	p__Bacteroidet	c__Bacteroidia	o__Bacteroidales	f__unclassified_	g__unclassified_o__Bacteroidales	s__unclassified_o__Bacteroidales
OTU475	p__Firmicutes	c__Clostridia	o__Clostridiales	f__Clostridiacea	g__Clostridium_sensu_stricto_10	s__uncultured_bacterium_g__Clostridium_sensu_stricto_10
OTU531	p__Bacteroidet	c__Bacteroidia	o__Bacteroidales	f__Porphyromo	g__Odoribacter	s__uncultured_bacterium_g__Odoribacter
OTU51	p__Bacteroidet	c__Bacteroidia	o__Bacteroidales	f__Bacteroidale	g__norank_f__Bacteroidales_S24-7_group	s__uncultured_bacterium_g__norank_f__Bacteroidales_S24-7

OTU661	p__Firmicutes	c__Clostridia	o__Clostridiales	f__Lachnospira	g__Lachnoclostridium	s__unclassified_g__Lachnoclostridium
OTU268	p__Proteobacte	c__Alphaprote	o__Rhodospirilla	f__Rhodospirill	g__norank_f__Rhodospirillaceae	s__gut_metagenome_g__norank_f__Rhodospirillaceae
OTU659	p__Firmicutes	c__Clostridia	o__Clostridiales	f__Lachnospira	g__unclassified_f__Lachnospiraceae	s__unclassified_f__Lachnospiraceae
OTU255	p__Firmicutes	c__Clostridia	o__Clostridiales	f__Clostridiales	g__norank_f__Clostridiales_vadinBB60_group	s__uncultured_bacterium_g__norank_f__Clostridiales_vadinB
OTU204	p__Bacteroidet	c__Bacteroidia	o__Bacteroidales	f__Bacteroidale	g__norank_f__Bacteroidales_S24-7_group	s__unclassified_g__norank_f__Bacteroidales_S24-7_group
OTU12	p__Bacteroidet	c__Bacteroidia	o__Bacteroidales	f__Bacteroidale	g__norank_f__Bacteroidales_S24-7_group	s__uncultured_bacterium_g__norank_f__Bacteroidales_S24-7
OTU669	p__Bacteroidet	c__Bacteroidia	o__Bacteroidales	f__Bacteroidale	g__norank_f__Bacteroidales_S24-7_group	s__uncultured_bacterium_g__norank_f__Bacteroidales_S24-7
OTU679	p__Firmicutes	c__Clostridia	o__Clostridiales	f__Lachnospira	g__Coprococcus_1	s__uncultured_bacterium_g__Coprococcus_1
OTU360	p__Firmicutes	c__Clostridia	o__Clostridiales	f__Lachnospira	g__Roseburia	s__uncultured_bacterium_g__Roseburia
OTU256	p__Firmicutes	c__Clostridia	o__Clostridiales	f__Clostridiales	g__norank_f__Clostridiales_vadinBB60_group	s__uncultured_bacterium_g__norank_f__Clostridiales_vadinB
OTU328	p__Bacteroidet	c__Bacteroidia	o__Bacteroidales	f__Bacteroidale	g__norank_f__Bacteroidales_S24-7_group	s__unclassified_g__norank_f__Bacteroidales_S24-7_group
OTU180	p__Firmicutes	c__Clostridia	o__Clostridiales	f__Ruminococc	g__Ruminococcaceae_UCG-014	s__unclassified_g__Ruminococcaceae_UCG-014
OTU657	p__Firmicutes	c__Clostridia	o__Clostridiales	f__Lachnospira	g__norank_f__Lachnospiraceae	s__unclassified_g__norank_f__Lachnospiraceae
OTU260	p__Bacteroidet	c__Bacteroidia	o__Bacteroidales	f__unclassified_	g__unclassified_o__Bacteroidales	s__unclassified_o__Bacteroidales
OTU209	p__Bacteroidet	c__Bacteroidia	o__Bacteroidales	f__Bacteroidale	g__norank_f__Bacteroidales_S24-7_group	s__uncultured_bacterium_g__norank_f__Bacteroidales_S24-7
OTU249	p__Bacteroidet	c__Flavobacter	o__Flavobacteria	f__Flavobacteri	g__norank_f__Flavobacteriaceae	s__gut_metagenome_g__norank_f__Flavobacteriaceae
OTU371	p__Firmicutes	c__Clostridia	o__Clostridiales	f__Lachnospira	g__unclassified_f__Lachnospiraceae	s__unclassified_f__Lachnospiraceae
OTU363	p__Firmicutes	c__Clostridia	o__Clostridiales	f__Ruminococc	g__Anaerotruncus	s__unclassified_g__Anaerotruncus
OTU96	p__Firmicutes	c__Clostridia	o__Clostridiales	f__Ruminococc	g__Anaerotruncus	s__uncultured_bacterium_g__Anaerotruncus
OTU415	p__Firmicutes	c__Clostridia	o__Clostridiales	f__Lachnospira	g__Blautia	s__unclassified_g__Blautia
OTU407	p__Firmicutes	c__Clostridia	o__Clostridiales	f__Lachnospira	g__unclassified_f__Lachnospiraceae	s__unclassified_f__Lachnospiraceae
OTU449	p__Firmicutes	c__Clostridia	o__Clostridiales	f__Ruminococc	g__Ruminiclostridium	s__uncultured_bacterium_g__Ruminiclostridium
OTU322	p__Bacteroidet	c__Bacteroidia	o__Bacteroidales	f__Porphyromo	g__Parabacteroides	s__unclassified_g__Parabacteroides
OTU516	p__Bacteroidet	c__Bacteroidia	o__Bacteroidales	f__Porphyromo	g__Butyricimonas	s__Butyricimonas_virosa
OTU402	p__Firmicutes	c__Clostridia	o__Clostridiales	f__Lachnospira	g__norank_f__Lachnospiraceae	s__unclassified_g__norank_f__Lachnospiraceae
OTU681	p__Actinobacte	c__Actinobacte	o__Coriobacteria	f__Coriobacteri	g__Gordonibacter	s__uncultured_bacterium_g__Gordonibacter
OTU507	p__Firmicutes	c__Clostridia	o__Clostridiales	f__Family_XIII	g__Family_XIII_UCG-001	s__uncultured_bacterium_g__Family_XIII_UCG-001
OTU639	p__Firmicutes	c__Clostridia	o__Clostridiales	f__Lachnospira	g__norank_f__Lachnospiraceae	s__unclassified_g__norank_f__Lachnospiraceae
OTU610	p__Firmicutes	c__Clostridia	o__Clostridiales	f__Clostridiales	g__norank_f__Clostridiales_vadinBB60_group	s__unclassified_g__norank_f__Clostridiales_vadinBB60_grou
OTU448	p__Firmicutes	c__Clostridia	o__Clostridiales	f__Lachnospira	g__norank_f__Lachnospiraceae	s__unclassified_g__norank_f__Lachnospiraceae
OTU600	p__Firmicutes	c__Clostridia	o__Clostridiales	f__Ruminococc	g__Anaerotruncus	s__unclassified_g__Anaerotruncus
OTU581	p__Firmicutes	c__Clostridia	o__Clostridiales	f__Ruminococc	g__Ruminiclostridium_9	s__unclassified_g__Ruminiclostridium_9
OTU324	p__Bacteroidet	c__Bacteroidia	o__Bacteroidales	f__Porphyromo	g__Parabacteroides	s__Parabacteroides_goldsteinii
OTU636	p__Bacteroidet	c__Bacteroidia	o__Bacteroidales	f__Prevotellace	g__Paraprevotella	s__unclassified_g__Paraprevotella
OTU495	p__Firmicutes	c__Clostridia	o__Clostridiales	f__Lachnospira	g__unclassified_f__Lachnospiraceae	s__unclassified_f__Lachnospiraceae
OTU487	p__Bacteroidet	c__Bacteroidia	o__Bacteroidales	f__Bacteroidale	g__norank_f__Bacteroidales_S24-7_group	s__uncultured_bacterium_g__norank_f__Bacteroidales_S24-7
OTU259	p__Tenericutes	c__Mollicutes	o__Mollicutes_R	f__norank_o__	g__norank_o__Mollicutes_RF9	s__uncultured_Erysipelotrichaceae_bacterium_g__norank_o_
OTU378	p__Firmicutes	c__Clostridia	o__Clostridiales	f__Lachnospira	g__Tyzzerella	s__uncultured_bacterium_g__Tyzzerella
OTU527	p__Bacteroidet	c__Bacteroidia	o__Bacteroidales	f__Bacteroidac	g__Bacteroides	s__unclassified_g__Bacteroides

OTU39	p__Firmicutes	c__Clostridia	o__Clostridiales	f__Ruminococc	g__Ruminococcaceae_UCG-014	s__unclassified_g__Ruminococcaceae_UCG-014
OTU323	p__Bacteroidet	c__Bacteroidia	o__Bacteroidales	f__Rikenellacea	g__Alistipes	s__uncultured_bacterium_g__Alistipes
OTU252	p__Bacteroidet	c__Bacteroidia	o__Bacteroidales	f__unclassified_	g__unclassified_o__Bacteroidales	s__unclassified_o__Bacteroidales
OTU410	p__Firmicutes	c__Clostridia	o__Clostridiales	f__Ruminococc	g__Faecalibacterium	s__unclassified_g__Faecalibacterium
OTU532	p__Bacteroidet	c__Bacteroidia	o__Bacteroidales	f__Bacteroidale	g__norank_f__Bacteroidales_S24-7_group	s__unclassified_g__norank_f__Bacteroidales_S24-7_group
OTU680	p__Firmicutes	c__Clostridia	o__Clostridiales	f__Lachnospira	g__Coprococcus_1	s__unclassified_g__Coprococcus_1
OTU166	p__Actinobacte	c__Actinobacte	o__Coriobacteria	f__Coriobacteri	g__Enterorhabdus	s__uncultured_bacterium_g__Enterorhabdus
OTU694	p__Firmicutes	c__Clostridia	o__Clostridiales	f__Lachnospira	g__unclassified_f__Lachnospiraceae	s__unclassified_f__Lachnospiraceae
OTU317	p__Bacteroidet	c__Bacteroidia	o__Bacteroidales	f__Bacteroidale	g__norank_f__Bacteroidales_S24-7_group	s__unclassified_g__norank_f__Bacteroidales_S24-7_group
OTU506	p__Firmicutes	c__Clostridia	o__Clostridiales	f__Ruminococc	g__Ruminiclostridium_9	s__uncultured_bacterium_g__Ruminiclostridium_9
OTU113	p__Bacteroidet	c__Bacteroidia	o__Bacteroidales	f__Bacteroidale	g__norank_f__Bacteroidales_S24-7_group	s__uncultured_bacterium_g__norank_f__Bacteroidales_S24-7
OTU174	p__Firmicutes	c__Clostridia	o__Clostridiales	f__Lachnospira	g__Lachnospiraceae_UCG-001	s__unclassified_g__Lachnospiraceae_UCG-001
OTU432	p__Firmicutes	c__Clostridia	o__Clostridiales	f__Lachnospira	g__unclassified_f__Lachnospiraceae	s__unclassified_f__Lachnospiraceae
OTU592	p__Firmicutes	c__Clostridia	o__Clostridiales	f__Lachnospira	g__Lachnospiraceae_NK4A136_group	s__uncultured_bacterium_g__Lachnospiraceae_NK4A136_gr
OTU440	p__Actinobacte	c__Actinobacte	o__Propionibacte	f__Propionibact	g__Propionibacterium	s__Propionibacterium_acnes_subsp._acnes_g__Propionibacte
OTU523	p__Firmicutes	c__Bacilli	o__Lactobacillal	f__Streptococca	g__Streptococcus	s__Streptococcus_hyointestinalis
OTU214	p__Actinobacte	c__Actinobacte	o__Coriobacteria	f__Coriobacteri	g__Enterorhabdus	s__uncultured_bacterium_g__Enterorhabdus
OTU651	p__Bacteroidet	c__Bacteroidia	o__Bacteroidales	f__Bacteroidale	g__norank_f__Bacteroidales_S24-7_group	s__uncultured_bacterium_g__norank_f__Bacteroidales_S24-7
OTU201	p__Actinobacte	c__Actinobacte	o__Coriobacteria	f__Coriobacteri	g__Parvibacter	s__uncultured_bacterium_g__Parvibacter
OTU631	p__Firmicutes	c__Erysipelotri	o__Erysipelotricl	f__Erysipelotric	g__norank_f__Erysipelotrichaceae	s__unclassified_g__norank_f__Erysipelotrichaceae
OTU560	p__Firmicutes	c__Clostridia	o__Clostridiales	f__Lachnospira	g__unclassified_f__Lachnospiraceae	s__unclassified_f__Lachnospiraceae
OTU571	p__Firmicutes	c__Clostridia	o__Clostridiales	f__Lachnospira	g__unclassified_f__Lachnospiraceae	s__unclassified_f__Lachnospiraceae
OTU684	p__Cyanobacte	c__Cyanobacte	o__Gastranaerop	f__norank_o__(	g__norank_o__Gastranaerophilales	s__uncultured_bacterium_g__norank_o__Gastranaerophilales
OTU490	p__Bacteroidet	c__Bacteroidia	o__Bacteroidales	f__Bacteroidale	g__norank_f__Bacteroidales_S24-7_group	s__unclassified_g__norank_f__Bacteroidales_S24-7_group
OTU618	p__Firmicutes	c__Clostridia	o__Clostridiales	f__Ruminococc	g__Anaerotruncus	s__uncultured_bacterium_g__Anaerotruncus
OTU247	p__Firmicutes	c__Clostridia	o__Clostridiales	f__Lachnospira	g__Lachnospiraceae_NK4A136_group	s__uncultured_bacterium_g__Lachnospiraceae_NK4A136_gr
OTU675	p__Firmicutes	c__Clostridia	o__Clostridiales	f__Lachnospira	g__norank_f__Lachnospiraceae	s__mouse_gut_metagenome_g__norank
OTU295	p__Bacteroidet	c__Bacteroidia	o__Bacteroidales	f__Bacteroidale	g__norank_f__Bacteroidales_S24-7_group	s__unclassified_g__norank_f__Bacteroidales_S24-7_group
OTU108	p__Firmicutes	c__Clostridia	o__Clostridiales	f__Ruminococc	g__Ruminococcaceae_UCG-013	s__uncultured_organism_g__Ruminococcaceae_UCG-013
OTU594	p__Firmicutes	c__Clostridia	o__Clostridiales	f__Ruminococc	g__unclassified_f__Ruminococcaceae	s__unclassified_f__Ruminococcaceae
OTU697	p__Firmicutes	c__Clostridia	o__Clostridiales	f__Lachnospira	g__norank_f__Lachnospiraceae	s__unclassified_g__norank_f__Lachnospiraceae
OTU267	p__Proteobacte	c__Alphaprote	o__Rhodospirilla	f__Rhodospirill	g__norank_f__Rhodospirillaceae	s__gut_metagenome_g__norank_f__Rhodospirillaceae
OTU68	p__Firmicutes	c__Clostridia	o__Clostridiales	f__Lachnospira	g__Lachnospiraceae_NK4A136_group	s__unclassified_g__Lachnospiraceae_NK4A136_group
OTU445	p__Deferribacte	c__Deferribact	o__Deferribacter	f__Deferribacte	g__Mucispirillum	s__uncultured_bacterium_g__Mucispirillum
OTU167	p__Bacteroidet	c__Bacteroidia	o__Bacteroidales	f__Bacteroidale	g__norank_f__Bacteroidales_S24-7_group	s__uncultured_bacterium_g__norank_f__Bacteroidales_S24-7
OTU393	p__Firmicutes	c__Clostridia	o__Clostridiales	f__Ruminococc	g__Ruminococcaceae_UCG-014	s__unclassified_g__Ruminococcaceae_UCG-014
OTU235	p__Firmicutes	c__Clostridia	o__Clostridiales	f__Family_XIII	g__Anaerovorax	s__uncultured_bacterium_g__Anaerovorax
OTU454	p__Firmicutes	c__Clostridia	o__Clostridiales	f__Lachnospira	g__Lachnospiraceae_NK4A136_group	s__unclassified_g__Lachnospiraceae_NK4A136_group
OTU101	p__Proteobacte	c__Gammaprot	o__Enterobacteri	f__Enterobacte	g__Escherichia-Shigella	s__unclassified_g__Escherichia-Shigella

OTU372	p__Bacteroidet	c__Bacteroidia	o__Bacteroidales	f__Bacteroidale	g__norank_f__Bacteroidales_S24-7_group		s__uncultured_bacterium_g__norank_f__Bacteroidales_S24-7
OTU3	p__Firmicutes	c__Clostridia	o__Clostridiales	f__Lachnospira	g__unclassified_f__Lachnospiraceae		s__unclassified_f__Lachnospiraceae
OTU169	p__Actinobacte	c__Actinobacte	o__Coriobacteria	f__Coriobacteri	g__Enterorhabdus		s__uncultured_bacterium_g__Enterorhabdus
OTU130	p__Firmicutes	c__Clostridia	o__Clostridiales	f__Ruminococc	g__Ruminiclostridium_5		s__uncultured_bacterium_g__Ruminiclostridium_5
OTU469	p__Firmicutes	c__Clostridia	o__Clostridiales	f__Ruminococc	g__unclassified_f__Ruminococcaceae		s__unclassified_f__Ruminococcaceae
OTU404	p__Firmicutes	c__Clostridia	o__Clostridiales	f__Clostridiales	g__norank_f__Clostridiales_vadinBB60_group		s__uncultured_bacterium_g__norank_f__Clostridiales_vadinB
OTU81	p__Firmicutes	c__Clostridia	o__Clostridiales	f__Lachnospira	g__unclassified_f__Lachnospiraceae		s__unclassified_f__Lachnospiraceae
OTU65	p__Firmicutes	c__Clostridia	o__Clostridiales	f__Ruminococc	g__unclassified_f__Ruminococcaceae		s__unclassified_f__Ruminococcaceae
OTU44	p__Firmicutes	c__Clostridia	o__Clostridiales	f__Ruminococc	g__unclassified_f__Ruminococcaceae		s__unclassified_f__Ruminococcaceae
OTU195	p__Bacteroidet	c__Bacteroidia	o__Bacteroidales	f__Bacteroidale	g__norank_f__Bacteroidales_S24-7_group		s__uncultured_bacterium_g__norank_f__Bacteroidales_S24-7
OTU365	p__Firmicutes	c__Clostridia	o__Clostridiales	f__Lachnospira	g__[Eubacterium]_fissicatena_group		s__unclassified_g__[Eubacterium]_fissicatena_group
OTU326	p__Bacteroidet	c__Bacteroidia	o__Bacteroidales	f__Porphyromo	g__Parabacteroides		s__unclassified_g__Parabacteroides
OTU258	p__Firmicutes	c__Clostridia	o__Clostridiales	f__Ruminococc	g__Anaerotruncus		s__Anaerotruncus_sp._G3_2012_
OTU359	p__Bacteroidet	c__Bacteroidia	o__Bacteroidales	f__Bacteroidale	g__norank_f__Bacteroidales_S24-7_group		s__uncultured_bacterium_g__norank_f__Bacteroidales_S24-7
OTU240	p__Firmicutes	c__Clostridia	o__Clostridiales	f__Clostridiales	g__norank_f__Clostridiales_vadinBB60_group		s__uncultured_bacterium_g__norank_f__Clostridiales_vadinB
OTU234	p__Tenericutes	c__Mollicutes	o__Mollicutes_R	f__norank_o__I	g__norank_o__Mollicutes_RF9		s__uncultured_bacterium_g__norank_o__Mollicutes_RF9
OTU542	p__Firmicutes	c__Erysipelotri	o__Erysipelotricl	f__Erysipelotric	g__unclassified_f__Erysipelotrichaceae		s__unclassified_f__Erysipelotrichaceae
OTU83	p__Firmicutes	c__Clostridia	o__Clostridiales	f__Ruminococc	g__Ruminococcaceae_UCG-014		s__unclassified_g__Ruminococcaceae_UCG-014
OTU642	p__Firmicutes	c__Clostridia	o__Clostridiales	f__Lachnospira	g__unclassified_f__Lachnospiraceae		s__unclassified_f__Lachnospiraceae
OTU70	p__Firmicutes	c__Clostridia	o__Clostridiales	f__Ruminococc	g__Ruminococcaceae_UCG-005		s__uncultured_Clostridiales_bacterium_g__Ruminococcaceae
OTU491	p__Bacteroidet	c__Bacteroidia	o__Bacteroidales	f__Prevotellace	g__unclassified_f__Prevotellaceae		s__unclassified_f__Prevotellaceae
OTU708	p__Firmicutes	c__Clostridia	o__Clostridiales	f__Lachnospira	g__Lachnospiraceae_UCG-001		s__uncultured_Clostridiales_bacterium_g__Lachnospiraceae_
OTU269	p__Firmicutes	c__Clostridia	o__Clostridiales	f__Lachnospira	g__Lachnoclostridium		s__unclassified_g__Lachnoclostridium
OTU461	p__Firmicutes	c__Clostridia	o__Clostridiales	f__Lachnospira	g__norank_f__Lachnospiraceae		s__unclassified_g__norank_f__Lachnospiraceae
OTU150	p__Actinobacte	c__Actinobacte	o__Bifidobacteri	f__Bifidobacter	g__Bifidobacterium		s__Bifidobacterium_pseudolongum_PV8-2
OTU299	p__Cyanobacte	c__Cyanobacte	o__norank_c__C	f__norank_c__(	g__norank_c__Cyanobacteria		s__unclassified_g__norank_c__Cyanobacteria
OTU264	p__Firmicutes	c__Clostridia	o__Clostridiales	f__Lachnospira	g__Coprococcus_1		s__uncultured_bacterium_g__Coprococcus_1
OTU574	p__Bacteroidet	c__Bacteroidia	o__Bacteroidales	f__Bacteroidale	g__norank_f__Bacteroidales_S24-7_group		s__uncultured_bacterium_g__norank_f__Bacteroidales_S24-7
OTU616	p__Firmicutes	c__Clostridia	o__Clostridiales	f__Lachnospira	g__norank_f__Lachnospiraceae		s__uncultured_bacterium_g__norank_f__Lachnospiraceae
OTU577	p__Bacteroidet	c__Bacteroidia	o__Bacteroidales	f__Prevotellace	g__Prevotellaceae_UCG-003		s__unclassified_g__Prevotellaceae_UCG-003
OTU84	p__Bacteroidet	c__Bacteroidia	o__Bacteroidales	f__Bacteroidale	g__norank_f__Bacteroidales_S24-7_group		s__uncultured_bacterium_g__norank_f__Bacteroidales_S24-7
OTU74	p__Tenericutes	c__Mollicutes	o__Mollicutes_R	f__norank_o__I	g__norank_o__Mollicutes_RF9		s__unclassified_g__norank_o__Mollicutes_RF9
OTU69	p__Firmicutes	c__Clostridia	o__Clostridiales	f__Ruminococc	g__Ruminococcaceae_NK4A214_group		s__uncultured_bacterium_g__Ruminococcaceae_NK4A214_g
OTU485	p__Proteobacte	c__Betaproteob	o__Burkholderial	f__Alcaligenace	g__Parasutterella		s__uncultured_bacterium_g__Parasutterella
OTU207	p__Bacteroidet	c__Bacteroidia	o__Bacteroidales	f__Bacteroidale	g__norank_f__Bacteroidales_S24-7_group		s__uncultured_bacterium_g__norank_f__Bacteroidales_S24-7
OTU47	p__Bacteroidet	c__Bacteroidia	o__Bacteroidales	f__Bacteroidale	g__norank_f__Bacteroidales_S24-7_group		s__uncultured_bacterium_g__norank_f__Bacteroidales_S24-7
OTU145	p__Bacteroidet	c__Bacteroidia	o__Bacteroidales	f__Bacteroidale	g__norank_f__Bacteroidales_S24-7_group		s__uncultured_bacterium_g__norank_f__Bacteroidales_S24-7
OTU243	p__Bacteroidet	c__Bacteroidia	o__Bacteroidales	f__Bacteroidale	g__norank_f__Bacteroidales_S24-7_group		s__uncultured_bacterium_g__norank_f__Bacteroidales_S24-7
OTU477	p__Firmicutes	c__Clostridia	o__Clostridiales	f__Lachnospira	g__unclassified_f__Lachnospiraceae		s__unclassified_f__Lachnospiraceae

OTU148	p__Firmicutes	c__Bacilli	o__Lactobacillale	f__Enterococca	g__Enterococcus	s__unclassified_g__Enterococcus
OTU320	p__Bacteroidete	c__Bacteroidia	o__Bacteroidales	f__Bacteroidace	g__Bacteroides	s__Bacteroides_acidifaciens
OTU301	p__Firmicutes	c__Clostridia	o__Clostridiales	f__Lachnospira	g__Roseburia	s__uncultured_bacterium_g__Roseburia
OTU635	p__Firmicutes	c__Clostridia	o__Clostridiales	f__Lachnospira	g__unclassified_f__Lachnospiraceae	s__unclassified_f__Lachnospiraceae
OTU147	p__Cyanobacte	c__Cyanobacte	o__Gastranaerop	f__norank_o__(	g__norank_o__Gastranaerophilales	s__uncultured_rumen_bacterium_g__norank_o__Gastranaero
OTU678	p__Proteobacte	c__Epsilonprot	o__Campylobact	f__Helicobacte	g__Helicobacter	s__Helicobacter_mastomyrinus
OTU15	p__Bacteroidete	c__Bacteroidia	o__Bacteroidales	f__Bacteroidale	g__norank_f__Bacteroidales_S24-7_group	s__unclassified_g__norank_f__Bacteroidales_S24-7_group
OTU278	p__Proteobacte	c__Deltaproteo	o__Desulfovibric	f__Desulfovibri	g__Desulfovibrio	s__uncultured_bacterium_g__Desulfovibrio
OTU514	p__Bacteroidete	c__Bacteroidia	o__Bacteroidales	f__Rikenellacea	g__Rikenellaceae_RC9_gut_group	s__uncultured_bacterium_g__Rikenellaceae_RC9_gut_group
OTU408	p__Firmicutes	c__Clostridia	o__Clostridiales	f__Lachnospira	g__norank_f__Lachnospiraceae	s__uncultured_bacterium_g__norank_f__Lachnospiraceae
OTU60	p__Firmicutes	c__Clostridia	o__Clostridiales	f__Lachnospira	g__unclassified_f__Lachnospiraceae	s__unclassified_f__Lachnospiraceae
OTU346	p__Firmicutes	c__Clostridia	o__Clostridiales	f__Lachnospira	g__Lachnospiraceae_UCG-006	s__uncultured_bacterium_g__Lachnospiraceae_UCG-006
OTU494	p__Firmicutes	c__Clostridia	o__Clostridiales	f__Ruminococc	g__unclassified_f__Ruminococcaceae	s__unclassified_f__Ruminococcaceae
OTU262	p__Bacteroidete	c__Bacteroidia	o__Bacteroidales	f__Bacteroidace	g__Bacteroides	s__unclassified_g__Bacteroides
OTU321	p__Bacteroidete	c__Bacteroidia	o__Bacteroidales	f__Rikenellacea	g__Alistipes	s__gut_metagenome_g__Alistipes
OTU335	p__Firmicutes	c__Clostridia	o__Clostridiales	f__Clostridiales	g__norank_f__Clostridiales_vadinBB60_group	s__unclassified_g__norank_f__Clostridiales_vadinBB60_grou
OTU227	p__Firmicutes	c__Clostridia	o__Clostridiales	f__Lachnospira	g__norank_f__Lachnospiraceae	s__Clostridium_sp._Clone-47
OTU395	p__Firmicutes	c__Erysipelotri	o__Erysipelotricl	f__Erysipelotric	g__Faecalibaculum	s__uncultured_bacterium_g__Faecalibaculum
OTU35	p__Proteobacte	c__Betaproteob	o__Burkholderia	f__Alcaligenace	g__Parasutterella	s__uncultured_organism_g__Parasutterella
OTU567	p__Firmicutes	c__Clostridia	o__Clostridiales	f__Lachnospira	g__norank_f__Lachnospiraceae	s__unclassified_g__norank_f__Lachnospiraceae
OTU665	p__Firmicutes	c__Clostridia	o__Clostridiales	f__Ruminococc	g__unclassified_f__Ruminococcaceae	s__unclassified_f__Ruminococcaceae
OTU693	p__Firmicutes	c__Clostridia	o__Clostridiales	f__Lachnospira	g__norank_f__Lachnospiraceae	s__unclassified_g__norank_f__Lachnospiraceae
OTU228	p__Firmicutes	c__Clostridia	o__Clostridiales	f__Lachnospira	g__unclassified_f__Lachnospiraceae	s__unclassified_f__Lachnospiraceae
OTU387	p__Proteobacte	c__Deltaproteo	o__Desulfovibric	f__Desulfovibri	g__Desulfovibrio	s__unclassified_g__Desulfovibrio
OTU535	p__Bacteroidete	c__Bacteroidia	o__Bacteroidales	f__Rikenellacea	g__Rikenella	s__Rikenella_microfusus_DSM_15922
OTU530	p__Bacteroidete	c__Bacteroidia	o__Bacteroidales	f__Bacteroidale	g__norank_f__Bacteroidales_S24-7_group	s__uncultured_Bacteroidales_bacterium_g__norank_f__Bacte
OTU211	p__Bacteroidete	c__Bacteroidia	o__Bacteroidales	f__Porphyromo	g__Parabacteroides	s__Parabacteroides_merdae
OTU11	p__Bacteroidete	c__Bacteroidia	o__Bacteroidales	f__Bacteroidale	g__norank_f__Bacteroidales_S24-7_group	s__uncultured_bacterium_g__norank_f__Bacteroidales_S24-7
OTU14	p__Bacteroidete	c__Bacteroidia	o__Bacteroidales	f__Bacteroidale	g__norank_f__Bacteroidales_S24-7_group	s__uncultured_bacterium_g__norank_f__Bacteroidales_S24-7
OTU374	p__Firmicutes	c__Erysipelotri	o__Erysipelotricl	f__Erysipelotric	g__Faecalitalea	s__[Eubacterium]_dolichum
OTU509	p__Bacteroidete	c__Bacteroidia	o__Bacteroidales	f__Bacteroidale	g__norank_f__Bacteroidales_S24-7_group	s__uncultured_bacterium_g__norank_f__Bacteroidales_S24-7
OTU464	p__Bacteroidete	c__Bacteroidia	o__Bacteroidales	f__Bacteroidale	g__norank_f__Bacteroidales_S24-7_group	s__uncultured_bacterium_g__norank_f__Bacteroidales_S24-7
OTU501	p__Firmicutes	c__Clostridia	o__Clostridiales	f__Ruminococc	g__Anaerotruncus	s__unclassified_g__Anaerotruncus
OTU89	p__Firmicutes	c__Erysipelotri	o__Erysipelotricl	f__Erysipelotric	g__Allobaculum	s__uncultured_bacterium_g__Allobaculum
OTU640	p__Firmicutes	c__Clostridia	o__Clostridiales	f__Ruminococc	g__norank_f__Ruminococcaceae	s__unclassified_g__norank_f__Ruminococcaceae
OTU476	p__Bacteroidete	c__Bacteroidia	o__Bacteroidales	f__Bacteroidale	g__norank_f__Bacteroidales_S24-7_group	s__uncultured_bacterium_g__norank_f__Bacteroidales_S24-7
OTU536	p__Bacteroidete	c__Bacteroidia	o__Bacteroidales	f__Bacteroidale	g__norank_f__Bacteroidales_S24-7_group	s__unclassified_g__norank_f__Bacteroidales_S24-7_group
OTU5	p__Firmicutes	c__Erysipelotri	o__Erysipelotricl	f__Erysipelotric	g__unclassified_f__Erysipelotrichaceae	s__unclassified_f__Erysipelotrichaceae
OTU94	p__Bacteroidete	c__Bacteroidia	o__Bacteroidales	f__Bacteroidale	g__norank_f__Bacteroidales_S24-7_group	s__uncultured_Bacteroidales_bacterium_g__norank_f__Bacte

OTU658	p__unclassified	c__unclassified	o__unclassified_l	f__unclassified_	g__unclassified_k__norank	s__unclassified_k__norank
OTU564	p__Bacteroidete	c__Bacteroidia	o__Bacteroidales	f__Rikenellacea	g__Alistipes	s__uncultured_bacterium_g__Alistipes
OTU193	p__Actinobacte	c__Actinobacte	o__Coriobacteria	f__Coriobacteri	g__Enterorhabdus	s__uncultured_bacterium_g__Enterorhabdus
OTU624	p__Firmicutes	c__Clostridia	o__Clostridiales	f__unclassified_	g__unclassified_o__Clostridiales	s__unclassified_o__Clostridiales
OTU238	p__Firmicutes	c__Bacilli	o__Lactobacillal	f__Lactobacilla	g__Lactobacillus	s__Lactobacillus_intestinalis
OTU338	p__Firmicutes	c__Clostridia	o__Clostridiales	f__Ruminococc	g__Anaerotruncus	s__uncultured_bacterium_g__Anaerotruncus
OTU334	p__Firmicutes	c__Clostridia	o__Clostridiales	f__Ruminococc	g__Anaerotruncus	s__uncultured_bacterium_g__Anaerotruncus
OTU121	p__Firmicutes	c__Clostridia	o__Clostridiales	f__Lachnospira	g__norank_f__Lachnospiraceae	s__unclassified_g__norank_f__Lachnospiraceae
OTU282	p__Firmicutes	c__Clostridia	o__Clostridiales	f__Lachnospira	g__norank_f__Lachnospiraceae	s__unclassified_g__norank_f__Lachnospiraceae
OTU435	p__Firmicutes	c__Clostridia	o__Clostridiales	f__Ruminococc	g__Oscillibacter	s__unclassified_g__Oscillibacter
OTU348	p__Proteobacte	c__Deltaproteo	o__Desulfovibric	f__Desulfovibri	g__Desulfovibrio	s__unclassified_g__Desulfovibrio
OTU115	p__Firmicutes	c__Clostridia	o__Clostridiales	f__Ruminococc	g__Ruminococcus_1	s__unclassified_g__Ruminococcus_1
OTU660	p__Proteobacte	c__Alphaprote	o__Caulobactera	f__Caulobacter	g__Brevundimonas	s__Brevundimonas_nasdae
OTU575	p__Firmicutes	c__Clostridia	o__Clostridiales	f__Ruminococc	g__unclassified_f__Ruminococcaceae	s__unclassified_f__Ruminococcaceae
OTU152	p__Actinobacte	c__Actinobacte	o__Corynebacter	f__Nocardiacea	g__Rhodococcus	s__unclassified_g__Rhodococcus
OTU90	p__Firmicutes	c__Clostridia	o__Clostridiales	f__Ruminococc	g__unclassified_f__Ruminococcaceae	s__unclassified_f__Ruminococcaceae
OTU589	p__Firmicutes	c__Clostridia	o__Clostridiales	f__Lachnospira	g__unclassified_f__Lachnospiraceae	s__unclassified_f__Lachnospiraceae
OTU580	p__Bacteroidete	c__Bacteroidia	o__Bacteroidales	f__Porphyromo	g__Odoribacter	s__uncultured_bacterium_g__Odoribacter
OTU290	p__Firmicutes	c__Clostridia	o__Clostridiales	f__Ruminococc	g__Anaerotruncus	s__uncultured_bacterium_g__Anaerotruncus
OTU254	p__Firmicutes	c__Clostridia	o__Clostridiales	f__Ruminococc	g__norank_f__Ruminococcaceae	s__unclassified_g__norank_f__Ruminococcaceae
OTU447	p__Firmicutes	c__Clostridia	o__Clostridiales	f__Lachnospira	g__norank_f__Lachnospiraceae	s__unclassified_g__norank_f__Lachnospiraceae
OTU177	p__Bacteroidete	c__Bacteroidia	o__Bacteroidales	f__Bacteroidale	g__norank_f__Bacteroidales_S24-7_group	s__uncultured_bacterium_g__norank_f__Bacteroidales_S24-7
OTU265	p__Bacteroidete	c__Bacteroidia	o__Bacteroidales	f__Bacteroidale	g__norank_f__Bacteroidales_S24-7_group	s__uncultured_bacterium_g__norank_f__Bacteroidales_S24-7
OTU472	p__Proteobacte	c__Deltaproteo	o__Desulfovibric	f__Desulfovibri	g__Desulfovibrio	s__unclassified_g__Desulfovibrio
OTU673	p__Firmicutes	c__Clostridia	o__Clostridiales	f__Ruminococc	g__Ruminococcaceae_UCG-014	s__unclassified_g__Ruminococcaceae_UCG-014
OTU439	p__Firmicutes	c__Clostridia	o__Clostridiales	f__Clostridiales	g__norank_f__Clostridiales_vadinBB60_group	s__uncultured_bacterium_g__norank_f__Clostridiales_vadinB
OTU253	p__Firmicutes	c__Clostridia	o__Clostridiales	f__Lachnospira	g__unclassified_f__Lachnospiraceae	s__unclassified_f__Lachnospiraceae
OTU171	p__Bacteroidete	c__Bacteroidia	o__Bacteroidales	f__Bacteroidale	g__norank_f__Bacteroidales_S24-7_group	s__uncultured_bacterium_g__norank_f__Bacteroidales_S24-7
OTU388	p__Firmicutes	c__Clostridia	o__Clostridiales	f__Clostridiales	g__norank_f__Clostridiales_vadinBB60_group	s__uncultured_bacterium_g__norank_f__Clostridiales_vadinB
OTU662	p__Firmicutes	c__Erysipelotri	o__Erysipelotricl	f__Erysipelotric	g__Erysipelatoclostridium	s__uncultured_bacterium_g__Erysipelatoclostridium
OTU607	p__Bacteroidete	c__Bacteroidia	o__Bacteroidales	f__unclassified_	g__unclassified_o__Bacteroidales	s__unclassified_o__Bacteroidales
OTU505	p__Firmicutes	c__Clostridia	o__Clostridiales	f__Ruminococc	g__unclassified_f__Ruminococcaceae	s__unclassified_f__Ruminococcaceae
OTU215	p__Firmicutes	c__Clostridia	o__Clostridiales	f__Ruminococc	g__Subdoligranulum	s__uncultured_bacterium_g__Subdoligranulum
OTU547	p__Bacteroidete	c__Bacteroidia	o__Bacteroidales	f__Bacteroidale	g__norank_f__Bacteroidales_S24-7_group	s__uncultured_bacterium_g__norank_f__Bacteroidales_S24-7
OTU300	p__Firmicutes	c__Clostridia	o__Clostridiales	f__Ruminococc	g__Ruminiclostridium_9	s__uncultured_organism_g__Ruminiclostridium_9
OTU304	p__Firmicutes	c__Clostridia	o__Clostridiales	f__Lachnospira	g__unclassified_f__Lachnospiraceae	s__unclassified_f__Lachnospiraceae
OTU173	p__Firmicutes	c__Bacilli	o__Bacillales	f__Staphylococ	g__Staphylococcus	s__Staphylococcus_nepalensis
OTU714	p__Proteobacte	c__Deltaproteo	o__Desulfovibric	f__Desulfovibri	g__Desulfovibrio	s__uncultured_bacterium_g__Desulfovibrio
OTU430	p__Proteobacte	c__Alphaprote	o__Rhodospirilla	f__Rhodospirill	g__norank_f__Rhodospirillaceae	s__unclassified_g__norank_f__Rhodospirillaceae

OTU696	p__Firmicutes	c__Clostridia	o__Clostridiales	f__Lachnospira	g__unclassified_f__Lachnospiraceae	s__unclassified_f__Lachnospiraceae
OTU297	p__Firmicutes	c__Clostridia	o__Clostridiales	f__Ruminococc	g__unclassified_f__Ruminococcaceae	s__unclassified_f__Ruminococcaceae
OTU330	p__Firmicutes	c__Clostridia	o__Clostridiales	f__Lachnospira	g__norank_f__Lachnospiraceae	s__unclassified_g__norank_f__Lachnospiraceae
OTU292	p__Actinobacte	c__Actinobacte	o__Coriobacteria	f__Coriobacteri	g__Coriobacteriaceae_UCG-002	s__uncultured_bacterium_g__Coriobacteriaceae_UCG-002
OTU380	p__Bacteroidet	c__Bacteroidia	o__Bacteroidales	f__Bacteroidale	g__norank_f__Bacteroidales_S24-7_group	s__uncultured_Bacteroidales_bacterium_g__norank_f__Bacte
OTU230	p__Firmicutes	c__Clostridia	o__Clostridiales	f__Ruminococc	g__Oscillibacter	s__uncultured_bacterium_g__Oscillibacter
OTU337	p__Firmicutes	c__Clostridia	o__Clostridiales	f__Ruminococc	g__Anaerotruncus	s__uncultured_bacterium_g__Anaerotruncus
OTU529	p__Bacteroidet	c__Bacteroidia	o__Bacteroidales	f__Prevotellace	g__unclassified_f__Prevotellaceae	s__unclassified_f__Prevotellaceae
OTU306	p__Firmicutes	c__Clostridia	o__Clostridiales	f__Lachnospira	g__Lachnospiraceae_NK4A136_group	s__uncultured_bacterium_g__Lachnospiraceae_NK4A136_gr
OTU176	p__Bacteroidet	c__Bacteroidia	o__Bacteroidales	f__Bacteroidale	g__norank_f__Bacteroidales_S24-7_group	s__uncultured_Bacteroidales_bacterium_g__norank_f__Bacte
OTU692	p__Firmicutes	c__unclassified	o__unclassified_	f__unclassified_	g__unclassified_p__Firmicutes	s__unclassified_p__Firmicutes
OTU543	p__Bacteroidet	c__Bacteroidia	o__Bacteroidales	f__Bacteroidale	g__norank_f__Bacteroidales_S24-7_group	s__unclassified_g__norank_f__Bacteroidales_S24-7_group
OTU125	p__Firmicutes	c__Clostridia	o__Clostridiales	f__Lachnospira	g__Lachnospiraceae_NK4A136_group	s__unclassified_g__Lachnospiraceae_NK4A136_group
OTU155	p__Cyanobacte	c__Cyanobacte	o__Gastranaerop	f__norank_o__	g__norank_o__Gastranaerophilales	s__uncultured_bacterium_g__norank_o__Gastranaerophilales
OTU314	p__Firmicutes	c__Erysipelotri	o__Erysipelotricl	f__Erysipelotric	g__Allobaculum	s__uncultured_bacterium_g__Allobaculum
OTU595	p__Firmicutes	c__Clostridia	o__Clostridiales	f__Ruminococc	g__Ruminococcaceae_UCG-014	s__unclassified_g__Ruminococcaceae_UCG-014
OTU117	p__Firmicutes	c__Clostridia	o__Clostridiales	f__Lachnospira	g__Roseburia	s__uncultured_bacterium_g__Roseburia
OTU690	p__Firmicutes	c__Clostridia	o__Clostridiales	f__Lachnospira	g__norank_f__Lachnospiraceae	s__unclassified_g__norank_f__Lachnospiraceae
OTU644	p__Firmicutes	c__Clostridia	o__Clostridiales	f__Ruminococc	g__Ruminiclostridium	s__unclassified_g__Ruminiclostridium
OTU375	p__Firmicutes	c__Clostridia	o__Clostridiales	f__Ruminococc	g__Ruminococcaceae_UCG-014	s__unclassified_g__Ruminococcaceae_UCG-014
OTU546	p__Firmicutes	c__Clostridia	o__Clostridiales	f__Clostridiace	g__Candidatus_Arthromitus	s__Candidatus_Arthromitus_sp._SFB-mouse-Japan
OTU628	p__Firmicutes	c__Clostridia	o__Clostridiales	f__Ruminococc	g__Ruminiclostridium	s__uncultured_bacterium_g__Ruminiclostridium
OTU590	p__Firmicutes	c__Clostridia	o__Clostridiales	f__Ruminococc	g__Ruminococcaceae_UCG-014	s__unclassified_g__Ruminococcaceae_UCG-014
OTU400	p__Saccharibac	c__Unknown_(	o__Unknown_Or	f__Unknown_F	g__Candidatus_Saccharimonas	s__uncultured_bacterium_g__Candidatus_Saccharimonas
OTU198	p__Bacteroidet	c__Bacteroidia	o__Bacteroidales	f__Bacteroidale	g__norank_f__Bacteroidales_S24-7_group	s__uncultured_Bacteroidales_bacterium_g__norank_f__Bacte
OTU9	p__Tenericutes	c__Mollicutes	o__Mollicutes_R	f__norank_o__l	g__norank_o__Mollicutes_RF9	s__uncultured_bacterium_g__norank_o__Mollicutes_RF9
OTU88	p__Proteobacte	c__Deltaproteo	o__Desulfovibric	f__Desulfovibri	g__Desulfovibrio	s__Desulfovibrio_sp._UNSW3caefatS
OTU623	p__Tenericutes	c__Mollicutes	o__Anaeroplasm	f__Anaeroplasn	g__Anaeroplasma	s__unclassified_g__Anaeroplasma
OTU212	p__Firmicutes	c__Clostridia	o__Clostridiales	f__Ruminococc	g__Ruminococcaceae_UCG-010	s__uncultured_bacterium_g__Ruminococcaceae_UCG-010
OTU615	p__Firmicutes	c__Clostridia	o__Clostridiales	f__Lachnospira	g__norank_f__Lachnospiraceae	s__uncultured_bacterium_g__norank_f__Lachnospiraceae
OTU352	p__Firmicutes	c__Clostridia	o__Clostridiales	f__Lachnospira	g__Roseburia	s__unclassified_g__Roseburia
OTU120	p__Firmicutes	c__Clostridia	o__Clostridiales	f__Lachnospira	g__unclassified_f__Lachnospiraceae	s__unclassified_f__Lachnospiraceae
OTU237	p__Firmicutes	c__Clostridia	o__Clostridiales	f__Lachnospira	g__norank_f__Lachnospiraceae	s__unclassified_g__norank_f__Lachnospiraceae
OTU22	p__Firmicutes	c__Clostridia	o__Clostridiales	f__Lachnospira	g__Roseburia	s__unclassified_g__Roseburia
OTU451	p__Bacteroidet	c__Bacteroidia	o__Bacteroidales	f__Rikenellacea	g__Alistipes	s__uncultured_bacterium_g__Alistipes
OTU508	p__Firmicutes	c__Clostridia	o__Clostridiales	f__Ruminococc	g__Oscillibacter	s__[Clostridium]_leptum_g__Oscillibacter
OTU570	p__Firmicutes	c__Clostridia	o__Clostridiales	f__Lachnospira	g__norank_f__Lachnospiraceae	s__Lachnospiraceae_bacterium_DW12
OTU231	p__Tenericutes	c__Mollicutes	o__Mollicutes_R	f__norank_o__l	g__norank_o__Mollicutes_RF9	s__unclassified_g__norank_o__Mollicutes_RF9
OTU46	p__Proteobacte	c__Alphaproteo	o__Rhodospirilla	f__Rhodospirill	g__norank_f__Rhodospirillaceae	s__uncultured_rumen_bacterium_g__norank_f__Rhodospirill

OTU236	p__Firmicutes	c__Clostridia	o__Clostridiales	f__Ruminococc	g__Anaerotruncus		s__uncultured_bacterium_g__Anaerotruncus
OTU583	p__Firmicutes	c__Clostridia	o__Clostridiales	f__Lachnospira	g__norank_f__Lachnospiraceae		s__Clostridium_sp._Culture-27
OTU311	p__Bacteroidet	c__Bacteroidia	o__Bacteroidales	f__unclassified_	g__unclassified_o__Bacteroidales		s__unclassified_o__Bacteroidales
OTU470	p__Firmicutes	c__Clostridia	o__Clostridiales	f__Lachnospira	g__unclassified_f__Lachnospiraceae		s__unclassified_f__Lachnospiraceae
OTU412	p__Firmicutes	c__Clostridia	o__Clostridiales	f__Lachnospira	g__Blautia		s__Blautia_coccoides_g__Blautia
OTU473	p__Firmicutes	c__Clostridia	o__Clostridiales	f__Ruminococc	g__Ruminococcaceae_UCG-014		s__[Clostridium]_papyrosolvens_g__Ruminococcaceae_UCG
OTU248	p__Firmicutes	c__Clostridia	o__Clostridiales	f__Ruminococc	g__Ruminococcaceae_NK4A214_group		s__unclassified_g__Ruminococcaceae_NK4A214_group
OTU699	p__Proteobacte	c__Deltaproteo	o__Desulfovibric	f__Desulfovibri	g__Desulfovibrio		s__unclassified_g__Desulfovibrio
OTU250	p__Firmicutes	c__Clostridia	o__Clostridiales	f__Lachnospira	g__Lachnospiraceae_NK4A136_group		s__uncultured_bacterium_g__Lachnospiraceae_NK4A136_gr
OTU576	p__Bacteroidet	c__Bacteroidia	o__Bacteroidales	f__Bacteroidale	g__norank_f__Bacteroidales_S24-7_group		s__uncultured_bacterium_g__norank_f__Bacteroidales_S24-7
OTU1	p__Firmicutes	c__Clostridia	o__Clostridiales	f__Clostridiales	g__norank_f__Clostridiales_vadinBB60_group		s__uncultured_bacterium_g__norank_f__Clostridiales_vadinI
OTU339	p__Firmicutes	c__Clostridia	o__Clostridiales	f__Ruminococc	g__Ruminococcaceae_UCG-013		s__unclassified_g__Ruminococcaceae_UCG-013
OTU425	p__Cyanobacte	c__Cyanobacte	o__Gastranaerop	f__norank_o__	g__norank_o__Gastranaerophilales		s__uncultured_bacterium_g__norank_o__Gastranaerophilales
OTU76	p__Firmicutes	c__Clostridia	o__Clostridiales	f__Ruminococc	g__Ruminococcaceae_UCG-005		s__uncultured_Clostridiales_bacterium_g__Ruminococcaceae
OTU695	p__Proteobacte	c__Gammaprot	o__Aeromonadal	f__Succinivibri	g__Anaerobiospirillum		s__unclassified_g__Anaerobiospirillum
OTU647	p__Firmicutes	c__Clostridia	o__Clostridiales	f__Ruminococc	g__Oscillibacter		s__unclassified_g__Oscillibacter
OTU409	p__Firmicutes	c__Clostridia	o__Clostridiales	f__Ruminococc	g__Butyricicoccus		s__unclassified_g__Butyricicoccus
OTU620	p__Firmicutes	c__Clostridia	o__Clostridiales	f__Clostridiales	g__norank_f__Clostridiales_vadinBB60_group		s__uncultured_bacterium_g__norank_f__Clostridiales_vadinI
OTU606	p__Bacteroidet	c__Bacteroidia	o__Bacteroidales	f__Bacteroidale	g__norank_f__Bacteroidales_S24-7_group		s__uncultured_bacterium_g__norank_f__Bacteroidales_S24-7
OTU202	p__Actinobacte	c__Actinobacte	o__Coriobacteria	f__Coriobacteri	g__Enterorhabdus		s__Enterorhabdus_mucosicola
OTU382	p__Firmicutes	c__Clostridia	o__Clostridiales	f__Lachnospira	g__Lachnospiraceae_NK4A136_group		s__uncultured_bacterium_g__Lachnospiraceae_NK4A136_gr
OTU192	p__Bacteroidet	c__Bacteroidia	o__Bacteroidales	f__Rikenellacea	g__Alistipes		s__uncultured_bacterium_g__Alistipes
OTU191	p__Bacteroidet	c__Bacteroidia	o__Bacteroidales	f__Rikenellacea	g__Alistipes		s__uncultured_bacterium_g__Alistipes
OTU73	p__Firmicutes	c__Clostridia	o__Clostridiales	f__Ruminococc	g__Ruminiclostridium_9		s__unclassified_g__Ruminiclostridium_9
OTU222	p__Actinobacte	c__Actinobacte	o__Bifidobacteri	f__Bifidobacter	g__Bifidobacterium		s__Bifidobacterium_pseudocatenulatum_DSM_20438__JCM_
OTU134	p__Proteobacte	c__Epsilonprot	o__Campylobact	f__Helicobacte	g__Helicobacter		s__Helicobacter_ganmani
OTU208	p__Bacteroidet	c__Bacteroidia	o__Bacteroidales	f__Bacteroidale	g__norank_f__Bacteroidales_S24-7_group		s__uncultured_bacterium_g__norank_f__Bacteroidales_S24-7
OTU518	p__Bacteroidet	c__Bacteroidia	o__Bacteroidales	f__Bacteroidale	g__norank_f__Bacteroidales_S24-7_group		s__unclassified_g__norank_f__Bacteroidales_S24-7_group
OTU138	p__Firmicutes	c__Clostridia	o__Clostridiales	f__Ruminococc	g__Anaerotruncus		s__uncultured_bacterium_g__Anaerotruncus
OTU184	p__Firmicutes	c__Clostridia	o__Clostridiales	f__Christensene	g__Christensenellaceae_R-7_group		s__unclassified_g__Christensenellaceae_R-7_group
OTU621	p__Firmicutes	c__Clostridia	o__Clostridiales	f__Lachnospira	g__norank_f__Lachnospiraceae		s__unclassified_g__norank_f__Lachnospiraceae
OTU569	p__Firmicutes	c__Clostridia	o__Clostridiales	f__Lachnospira	g__unclassified_f__Lachnospiraceae		s__unclassified_f__Lachnospiraceae
OTU511	p__Firmicutes	c__Clostridia	o__Clostridiales	f__Ruminococc	g__Anaerotruncus		s__unclassified_g__Anaerotruncus
OTU641	p__Firmicutes	c__Clostridia	o__Clostridiales	f__Ruminococc	g__norank_f__Ruminococcaceae		s__[Clostridium]_leptum_g__norank
OTU599	p__Bacteroidet	c__Bacteroidia	o__Bacteroidales	f__Porphyromo	g__Butyricimonas		s__unclassified_g__Butyricimonas
OTU141	p__Firmicutes	c__Clostridia	o__Clostridiales	f__Lachnospira	g__Lachnospiraceae_NK4A136_group		s__uncultured_Clostridiales_bacterium_g__Lachnospiraceae_
OTU32	p__Firmicutes	c__Clostridia	o__Clostridiales	f__Ruminococc	g__Anaerotruncus		s__unclassified_g__Anaerotruncus
OTU676	p__Proteobacte	c__Epsilonprot	o__Campylobact	f__Helicobacte	g__Helicobacter		s__unclassified_g__Helicobacter
OTU655	p__Proteobacte	c__Alphaprote	o__Rhodospirilla	f__Rhodospirill	g__norank_f__Rhodospirillaceae		s__gut_metagenome_g__norank_f__Rhodospirillaceae

OTU102	p__Firmicutes	c__Erysipelotri	o__Erysipelotricl	f__Erysipelotric	g__norank_f__Erysipelotrichaceae	s__uncultured_Erysipelotrichales_bacterium_g__norank
OTU396	p__Tenericutes	c__Mollicutes	o__Mollicutes_R	f__norank_o__l	g__norank_o__Mollicutes_RF9	s__unclassified_g__norank_o__Mollicutes_RF9
OTU617	p__Firmicutes	c__Clostridia	o__Clostridiales	f__Lachnospira	g__Blautia	s__unclassified_g__Blautia
OTU677	p__Firmicutes	c__Clostridia	o__Clostridiales	f__Ruminococc	g__Ruminiclostridium_6	s__uncultured_bacterium_g__Ruminiclostridium_6
OTU341	p__Spirochaeta	c__Spirochaete	o__Spirochaetale	f__Spirochaeta	g__Treponema_2	s__unclassified_g__Treponema_2
OTU394	p__Firmicutes	c__Bacilli	o__Lactobacillal	f__Lactobacilla	g__Lactobacillus	s__Lactobacillus_johnsonii
OTU563	p__Bacteroidet	c__Bacteroidia	o__Bacteroidales	f__Rikenellacea	g__Alistipes	s__Faecalibacterium_prausnitzii_g__Alistipes
OTU347	p__Firmicutes	c__Clostridia	o__Clostridiales	f__Lachnospira	g__norank_f__Lachnospiraceae	s__uncultured_bacterium_g__norank_f__Lachnospiraceae
OTU630	p__Bacteroidet	c__Bacteroidia	o__Bacteroidales	f__Prevotellace	g__Prevotellaceae_UCG-001	s__uncultured_Bacteroidales_bacterium_g__Prevotellaceae_U
OTU668	p__Firmicutes	c__Clostridia	o__Clostridiales	f__Lachnospira	g__norank_f__Lachnospiraceae	s__Lachnospiraceae_bacterium_DW8
OTU609	p__Bacteroidet	c__Bacteroidia	o__Bacteroidales	f__Bacteroidale	g__norank_f__Bacteroidales_S24-7_group	s__uncultured_bacterium_g__norank_f__Bacteroidales_S24-7
OTU364	p__Firmicutes	c__Clostridia	o__Clostridiales	f__Ruminococc	g__Anaerotruncus	s__unclassified_g__Anaerotruncus
OTU42	p__Bacteroidet	c__Bacteroidia	o__Bacteroidales	f__Bacteroidale	g__norank_f__Bacteroidales_S24-7_group	s__unclassified_g__norank_f__Bacteroidales_S24-7_group
OTU416	p__Firmicutes	c__Clostridia	o__Clostridiales	f__Ruminococc	g__Ruminococcus_1	s__unclassified_g__Ruminococcus_1
OTU561	p__Bacteroidet	c__Bacteroidia	o__Bacteroidales	f__Bacteroidale	g__norank_f__Bacteroidales_S24-7_group	s__uncultured_bacterium_g__norank_f__Bacteroidales_S24-7
OTU486	p__Bacteroidet	c__Bacteroidia	o__Bacteroidales	f__Rikenellacea	g__Rikenella	s__uncultured_bacterium_g__Rikenella
OTU718	p__Firmicutes	c__Clostridia	o__Clostridiales	f__Ruminococc	g__Butyricicoccus	s__unclassified_g__Butyricicoccus
OTU163	p__Firmicutes	c__Clostridia	o__Clostridiales	f__Lachnospira	g__Lachnospiraceae_NK4A136_group	s__uncultured_bacterium_g__Lachnospiraceae_NK4A136_gr
OTU674	p__Firmicutes	c__Clostridia	o__Clostridiales	f__Lachnospira	g__Roseburia	s__uncultured_bacterium_g__Roseburia
OTU197	p__Bacteroidet	c__Bacteroidia	o__Bacteroidales	f__Bacteroidale	g__norank_f__Bacteroidales_S24-7_group	s__uncultured_bacterium_g__norank_f__Bacteroidales_S24-7
OTU119	p__Firmicutes	c__Clostridia	o__Clostridiales	f__Lachnospira	g__unclassified_f__Lachnospiraceae	s__unclassified_f__Lachnospiraceae
OTU586	p__Firmicutes	c__Clostridia	o__Clostridiales	f__Clostridiales	g__norank_f__Clostridiales_vadinBB60_group	s__unclassified_g__norank_f__Clostridiales_vadinBB60_grou
OTU332	p__Proteobacte	c__Alphaprote	o__Rhodospirilla	f__Rhodospirill	g__norank_f__Rhodospirillaceae	s__gut_metagenome_g__norank_f__Rhodospirillaceae
OTU646	p__Tenericutes	c__Mollicutes	o__Mollicutes_R	f__norank_o__l	g__norank_o__Mollicutes_RF9	s__uncultured_bacterium_g__norank_o__Mollicutes_RF9
OTU437	p__Firmicutes	c__Erysipelotri	o__Erysipelotricl	f__Erysipelotric	g__unclassified_f__Erysipelotrichaceae	s__unclassified_f__Erysipelotrichaceae
OTU289	p__Firmicutes	c__Clostridia	o__Clostridiales	f__Lachnospira	g__norank_f__Lachnospiraceae	s__uncultured_bacterium_g__norank_f__Lachnospiraceae
OTU704	p__Firmicutes	c__Clostridia	o__Clostridiales	f__Lachnospira	g__norank_f__Lachnospiraceae	s__unclassified_g__norank_f__Lachnospiraceae
OTU318	p__Bacteroidet	c__Bacteroidia	o__Bacteroidales	f__Bacteroidale	g__norank_f__Bacteroidales_S24-7_group	s__uncultured_bacterium_g__norank_f__Bacteroidales_S24-7
OTU376	p__Firmicutes	c__Clostridia	o__Clostridiales	f__Clostridiales	g__norank_f__Clostridiales_vadinBB60_group	s__unclassified_g__norank_f__Clostridiales_vadinBB60_grou
OTU512	p__Bacteroidet	c__Bacteroidia	o__Bacteroidales	f__Bacteroidale	g__norank_f__Bacteroidales_S24-7_group	s__uncultured_bacterium_g__norank_f__Bacteroidales_S24-7
OTU353	p__Bacteroidet	c__Bacteroidia	o__Bacteroidales	f__Bacteroidale	g__norank_f__Bacteroidales_S24-7_group	s__uncultured_bacterium_g__norank_f__Bacteroidales_S24-7
OTU92	p__Bacteroidet	c__Bacteroidia	o__Bacteroidales	f__Bacteroidale	g__norank_f__Bacteroidales_S24-7_group	s__uncultured_bacterium_g__norank_f__Bacteroidales_S24-7
OTU597	p__Bacteroidet	c__Bacteroidia	o__Bacteroidales	f__Bacteroidale	g__norank_f__Bacteroidales_S24-7_group	s__uncultured_bacterium_g__norank_f__Bacteroidales_S24-7
OTU568	p__Bacteroidet	c__Bacteroidia	o__Bacteroidales	f__Bacteroidale	g__norank_f__Bacteroidales_S24-7_group	s__uncultured_bacterium_g__norank_f__Bacteroidales_S24-7
OTU172	p__Bacteroidet	c__Bacteroidia	o__Bacteroidales	f__unclassified_	g__unclassified_o__Bacteroidales	s__unclassified_o__Bacteroidales
OTU417	p__Proteobacte	c__Epsilonprot	o__Campylobact	f__Helicobacter	g__Helicobacter	s__Helicobacter_bilis
OTU441	p__Bacteroidet	c__Bacteroidia	o__Bacteroidales	f__Rikenellacea	g__Alistipes	s__Alistipes_indistinctus_YIT_12060
OTU105	p__Bacteroidet	c__Bacteroidia	o__Bacteroidales	f__Bacteroidale	g__norank_f__Bacteroidales_S24-7_group	s__uncultured_Bacteroidales_bacterium_g__norank_f__Bacte
OTU362	p__Firmicutes	c__Clostridia	o__Clostridiales	f__Lachnospira	g__norank_f__Lachnospiraceae	s__unclassified_g__norank_f__Lachnospiraceae

OTU142	p__Firmicutes	c__Clostridia	o__Clostridiales	f__Clostridiales	g__norank_f__Clostridiales_vadinBB60_group	s__unclassified_g__norank_f__Clostridiales_vadinBB60_grou
OTU588	p__Firmicutes	c__Clostridia	o__Clostridiales	f__Lachnospira	g__unclassified_f__Lachnospiraceae	s__unclassified_f__Lachnospiraceae
OTU601	p__Firmicutes	c__Clostridia	o__Clostridiales	f__Clostridiales	g__norank_f__Clostridiales_vadinBB60_group	s__unclassified_g__norank_f__Clostridiales_vadinBB60_grou
OTU63	p__Firmicutes	c__Clostridia	o__Clostridiales	f__Lachnospira	g__Lachnospiraceae_UCG-006	s__uncultured_bacterium_g__Lachnospiraceae_UCG-006
OTU183	p__Firmicutes	c__Clostridia	o__Clostridiales	f__Lachnospira	g__unclassified_f__Lachnospiraceae	s__unclassified_f__Lachnospiraceae
OTU50	p__Firmicutes	c__Clostridia	o__Clostridiales	f__Lachnospira	g__Lachnospiraceae_NK4A136_group	s__unclassified_g__Lachnospiraceae_NK4A136_group
OTU539	p__Firmicutes	c__Clostridia	o__Clostridiales	f__Ruminococc	g__Ruminococcaceae_UCG-014	s__unclassified_g__Ruminococcaceae_UCG-014
OTU216	p__Actinobacte	c__Actinobacte	o__Bifidobacteri	f__Bifidobacter	g__Bifidobacterium	s__unclassified_g__Bifidobacterium
OTU143	p__Firmicutes	c__Clostridia	o__Clostridiales	f__Ruminococc	g__Ruminococcaceae_UCG-014	s__uncultured_bacterium_g__Ruminococcaceae_UCG-014
OTU565	p__unclassified	c__unclassified	o__unclassified_	f__unclassified_	g__unclassified_k__norank	s__unclassified_k__norank
OTU450	p__Firmicutes	c__Clostridia	o__Clostridiales	f__Ruminococc	g__Ruminococcaceae_UCG-010	s__uncultured_organism_g__Ruminococcaceae_UCG-010
OTU13	p__Bacteroidet	c__Bacteroidia	o__Bacteroidales	f__Bacteroidale	g__norank_f__Bacteroidales_S24-7_group	s__uncultured_bacterium_g__norank_f__Bacteroidales_S24-7
OTU554	p__Bacteroidet	c__Bacteroidia	o__Bacteroidales	f__Prevotellace	g__Prevotellaceae_UCG-003	s__unclassified_g__Prevotellaceae_UCG-003
OTU257	p__Firmicutes	c__Clostridia	o__Clostridiales	f__Ruminococc	g__Ruminococcaceae_UCG-014	s__unclassified_g__Ruminococcaceae_UCG-014
OTU462	p__Firmicutes	c__Clostridia	o__Clostridiales	f__Lachnospira	g__unclassified_f__Lachnospiraceae	s__unclassified_f__Lachnospiraceae
OTU559	p__Firmicutes	c__Clostridia	o__Clostridiales	f__Lachnospira	g__unclassified_f__Lachnospiraceae	s__unclassified_f__Lachnospiraceae
OTU61	p__Firmicutes	c__Clostridia	o__Clostridiales	f__Lachnospira	g__[Eubacterium]_xylanophilum_group	s__uncultured_bacterium_g__[Eubacterium]_xylanophilum_g
OTU272	p__Firmicutes	c__Clostridia	o__Clostridiales	f__Ruminococc	g__Ruminococcaceae_UCG-014	s__unclassified_g__Ruminococcaceae_UCG-014
OTU98	p__Firmicutes	c__Clostridia	o__Clostridiales	f__Family_XIII	g__[Eubacterium]_nodatum_group	s__unclassified_g__[Eubacterium]_nodatum_group
OTU239	p__Firmicutes	c__Clostridia	o__Clostridiales	f__Ruminococc	g__Ruminococcaceae_UCG-013	s__unclassified_g__Ruminococcaceae_UCG-013
OTU356	p__Firmicutes	c__Clostridia	o__Clostridiales	f__Ruminococc	g__Ruminococcus_1	s__unclassified_g__Ruminococcus_1
OTU112	p__Bacteroidet	c__Bacteroidia	o__Bacteroidales	f__Bacteroidale	g__norank_f__Bacteroidales_S24-7_group	s__uncultured_bacterium_g__norank_f__Bacteroidales_S24-7
OTU343	p__Firmicutes	c__Clostridia	o__Clostridiales	f__Lachnospira	g__Lachnospiraceae_NK4A136_group	s__uncultured_bacterium_g__Lachnospiraceae_NK4A136_gr
OTU43	p__Bacteroidet	c__Bacteroidia	o__Bacteroidales	f__Bacteroidale	g__norank_f__Bacteroidales_S24-7_group	s__uncultured_bacterium_g__norank_f__Bacteroidales_S24-7
OTU66	p__Firmicutes	c__Clostridia	o__Clostridiales	f__Lachnospira	g__Lachnospiraceae_NK4A136_group	s__unclassified_g__Lachnospiraceae_NK4A136_group
OTU245	p__Firmicutes	c__Clostridia	o__Clostridiales	f__Lachnospira	g__Eisenbergiella	s__unclassified_g__Eisenbergiella
OTU442	p__Firmicutes	c__Clostridia	o__Clostridiales	f__Lachnospira	g__Lachnospiraceae_UCG-010	s__uncultured_bacterium_g__Lachnospiraceae_UCG-010
OTU585	p__Firmicutes	c__Clostridia	o__Clostridiales	f__Lachnospira	g__unclassified_f__Lachnospiraceae	s__unclassified_f__Lachnospiraceae
OTU422	p__Proteobacte	c__Alphaprote	o__Rhodospirilla	f__Rhodospirill	g__norank_f__Rhodospirillaceae	s__gut_metagenome_g__norank_f__Rhodospirillaceae
OTU705	p__Firmicutes	c__Clostridia	o__Clostridiales	f__Lachnospira	g__norank_f__Lachnospiraceae	s__unclassified_g__norank_f__Lachnospiraceae
OTU513	p__Bacteroidet	c__Bacteroidia	o__Bacteroidales	f__Bacteroidale	g__norank_f__Bacteroidales_S24-7_group	s__uncultured_bacterium_g__norank_f__Bacteroidales_S24-7
OTU645	p__Firmicutes	c__Clostridia	o__Clostridiales	f__Ruminococc	g__Oscillibacter	s__unclassified_g__Oscillibacter
OTU149	p__Firmicutes	c__Clostridia	o__Clostridiales	f__Lachnospira	g__norank_f__Lachnospiraceae	s__uncultured_bacterium_g__norank_f__Lachnospiraceae
OTU478	p__Firmicutes	c__Clostridia	o__Clostridiales	f__Lachnospira	g__norank_f__Lachnospiraceae	s__unclassified_g__norank_f__Lachnospiraceae
OTU27	p__Bacteroidet	c__Bacteroidia	o__Bacteroidales	f__Bacteroidale	g__norank_f__Bacteroidales_S24-7_group	s__uncultured_bacterium_g__norank_f__Bacteroidales_S24-7
OTU701	p__Firmicutes	c__Clostridia	o__Clostridiales	f__Lachnospira	g__unclassified_f__Lachnospiraceae	s__unclassified_f__Lachnospiraceae
OTU391	p__Firmicutes	c__Clostridia	o__Clostridiales	f__Clostridiales	g__norank_f__Clostridiales_vadinBB60_group	s__uncultured_Clostridiales_bacterium_g__norank_f__Clostri
OTU182	p__Actinobacte	c__Actinobacte	o__Coriobacteria	f__Coriobacteri	g__unclassified_f__Coriobacteriaceae	s__unclassified_f__Coriobacteriaceae
OTU670	p__Firmicutes	c__Clostridia	o__Clostridiales	f__Lachnospira	g__Lachnospiraceae_UCG-001	s__uncultured_bacterium_g__Lachnospiraceae_UCG-001

OTU199	p__Bacteroidet	c__Bacteroidia	o__Bacteroidales	f__Rikenellacea	g__Alistipes	s__gut_metagenome_g__Alistipes
OTU273	p__Bacteroidet	c__Bacteroidia	o__Bacteroidales	f__Bacteroidale	g__norank_f__Bacteroidales_S24-7_group	s__uncultured_bacterium_g__norank_f__Bacteroidales_S24-7
OTU110	p__Actinobacte	c__Actinobacte	o__Coriobacteria	f__Coriobacteri	g__unclassified_f__Coriobacteriaceae	s__unclassified_f__Coriobacteriaceae
OTU312	p__Bacteroidet	c__Bacteroidia	o__Bacteroidales	f__Bacteroidale	g__norank_f__Bacteroidales_S24-7_group	s__uncultured_bacterium_g__norank_f__Bacteroidales_S24-7
OTU379	p__Firmicutes	c__Clostridia	o__Clostridiales	f__Clostridiales	g__norank_f__Clostridiales_vadinBB60_group	s__unclassified_g__norank_f__Clostridiales_vadinBB60_grou
OTU367	p__Bacteroidet	c__Bacteroidia	o__Bacteroidales	f__Prevotellace	g__Prevotellaceae_UCG-003	s__unclassified_g__Prevotellaceae_UCG-003
OTU287	p__Firmicutes	c__Bacilli	o__Lactobacillal	f__Streptococca	g__Streptococcus	s__unclassified_g__Streptococcus
OTU584	p__Tenericutes	c__Mollicutes	o__Mycoplasmat	f__Mycoplasma	g__Mycoplasma	s__Mycoplasma_sualvi
OTU6	p__Tenericutes	c__Mollicutes	o__Mollicutes_R	f__norank_o__l	g__norank_o__Mollicutes_RF9	s__unclassified_g__norank_o__Mollicutes_RF9
OTU123	p__Firmicutes	c__Clostridia	o__Clostridiales	f__Lachnospira	g__unclassified_f__Lachnospiraceae	s__unclassified_f__Lachnospiraceae
OTU133	p__Firmicutes	c__Clostridia	o__Clostridiales	f__Lachnospira	g__Coprococcus_1	s__unclassified_g__Coprococcus_1
OTU686	p__Firmicutes	c__Clostridia	o__Clostridiales	f__Lachnospira	g__[Eubacterium]_xylanophilum_group	s__uncultured_bacterium_g__[Eubacterium]_xylanophilum_g
OTU93	p__Cyanobacte	c__Cyanobacte	o__Gastranaerop	f__norank_o__(	g__norank_o__Gastranaerophilales	s__unclassified_g__norank_o__Gastranaerophilales
OTU629	p__Bacteroidet	c__Bacteroidia	o__Bacteroidales	f__Bacteroidale	g__norank_f__Bacteroidales_S24-7_group	s__uncultured_bacterium_g__norank_f__Bacteroidales_S24-7
OTU103	p__Tenericutes	c__Mollicutes	o__Mollicutes_R	f__norank_o__l	g__norank_o__Mollicutes_RF9	s__uncultured_bacterium_g__norank_o__Mollicutes_RF9
OTU593	p__Bacteroidet	c__Bacteroidia	o__Bacteroidales	f__Bacteroidale	g__norank_f__Bacteroidales_S24-7_group	s__uncultured_bacterium_g__norank_f__Bacteroidales_S24-7
OTU562	p__Firmicutes	c__Clostridia	o__Clostridiales	f__Lachnospira	g__unclassified_f__Lachnospiraceae	s__unclassified_f__Lachnospiraceae
OTU719	p__Firmicutes	c__Clostridia	o__Clostridiales	f__Ruminococc	g__Ruminiclostridium	s__unclassified_g__Ruminiclostridium
OTU111	p__Bacteroidet	c__Bacteroidia	o__Bacteroidales	f__Bacteroidale	g__norank_f__Bacteroidales_S24-7_group	s__uncultured_bacterium_g__norank_f__Bacteroidales_S24-7
OTU59	p__Firmicutes	c__Clostridia	o__Clostridiales	f__Ruminococc	g__Anaerotruncus	s__uncultured_bacterium_g__Anaerotruncus
OTU453	p__Firmicutes	c__Clostridia	o__Clostridiales	f__Lachnospira	g__norank_f__Lachnospiraceae	s__unclassified_g__norank_f__Lachnospiraceae
OTU23	p__Bacteroidet	c__Bacteroidia	o__Bacteroidales	f__Rikenellacea	g__Alistipes	s__unclassified_g__Alistipes
OTU2	p__Bacteroidet	c__Bacteroidia	o__Bacteroidales	f__Porphyromo	g__Parabacteroides	s__unclassified_g__Parabacteroides
OTU157	p__Proteobacte	c__Betaproteob	o__Neisseriales	f__Neisseriacea	g__unclassified_f__Neisseriaceae	s__unclassified_f__Neisseriaceae
OTU151	p__Firmicutes	c__Erysipelotri	o__Erysipelotrich	f__Erysipelotric	g__Allobaculum	s__uncultured_bacterium_g__Allobaculum
OTU122	p__Firmicutes	c__Clostridia	o__Clostridiales	f__Lachnospira	g__Lachnospiraceae_UCG-001	s__unclassified_g__Lachnospiraceae_UCG-001
OTU638	p__Firmicutes	c__Clostridia	o__Clostridiales	f__Ruminococc	g__Ruminococcaceae_UCG-005	s__uncultured_bacterium_g__Ruminococcaceae_UCG-005
OTU504	p__Tenericutes	c__Mollicutes	o__Mollicutes_R	f__norank_o__l	g__norank_o__Mollicutes_RF9	s__uncultured_Erysipelotrichaceae_bacterium_g__norank_o_
OTU688	p__Cyanobacte	c__Cyanobacte	o__Gastranaerop	f__norank_o__(	g__norank_o__Gastranaerophilales	s__unclassified_g__norank_o__Gastranaerophilales
OTU383	p__Firmicutes	c__Bacilli	o__Lactobacillal	f__Lactobacilla	g__Lactobacillus	s__unclassified_g__Lactobacillus
OTU544	p__Bacteroidet	c__Bacteroidia	o__Bacteroidales	f__Prevotellace	g__Prevotellaceae_UCG-001	s__uncultured_Bacteroidales_bacterium_g__Prevotellaceae_U
OTU109	p__Bacteroidet	c__Bacteroidia	o__Bacteroidales	f__Bacteroidale	g__norank_f__Bacteroidales_S24-7_group	s__uncultured_bacterium_g__norank_f__Bacteroidales_S24-7
OTU266	p__Bacteroidet	c__Bacteroidia	o__Bacteroidales	f__unclassified_	g__unclassified_o__Bacteroidales	s__unclassified_o__Bacteroidales
OTU672	p__Firmicutes	c__Clostridia	o__Clostridiales	f__Lachnospira	g__norank_f__Lachnospiraceae	s__unclassified_g__norank_f__Lachnospiraceae
OTU566	p__Bacteroidet	c__Bacteroidia	o__Bacteroidales	f__Bacteroidac	g__Bacteroides	s__Bacteroides_acidifaciens
OTU551	p__Bacteroidet	c__Bacteroidia	o__Bacteroidales	f__Bacteroidale	g__norank_f__Bacteroidales_S24-7_group	s__uncultured_bacterium_g__norank_f__Bacteroidales_S24-7
OTU285	p__Tenericutes	c__Mollicutes	o__Mollicutes_R	f__norank_o__l	g__norank_o__Mollicutes_RF9	s__unclassified_g__norank_o__Mollicutes_RF9
OTU604	p__Bacteroidet	c__Bacteroidia	o__Bacteroidales	f__Prevotellace	g__unclassified_f__Prevotellaceae	s__unclassified_f__Prevotellaceae
OTU344	p__Firmicutes	c__Bacilli	o__Lactobacillal	f__Streptococca	g__Streptococcus	s__Streptococcus_sp._HTS9

OTU572	p__Firmicutes	c__Clostridia	o__Clostridiales	f__Ruminococc	g__Anaerotruncus	s__uncultured_bacterium_g__Anaerotruncus
OTU188	p__Bacteroidet	c__Bacteroidia	o__Bacteroidales	f__Bacteroidale	g__norank_f__Bacteroidales_S24-7_group	s__uncultured_bacterium_g__norank_f__Bacteroidales_S24-7
OTU18	p__Firmicutes	c__Clostridia	o__Clostridiales	f__Lachnospira	g__[Eubacterium]_xylanophilum_group	s__uncultured_bacterium_g__[Eubacterium]_xylanophilum_g
OTU492	p__Bacteroidet	c__Bacteroidia	o__Bacteroidales	f__Bacteroidac	g__Bacteroides	s__unclassified_g__Bacteroides
OTU552	p__Bacteroidet	c__Bacteroidia	o__Bacteroidales	f__Prevotellace	g__Prevotellaceae_UCG-001	s__unclassified_g__Prevotellaceae_UCG-001
OTU170	p__Actinobacte	c__Actinobacte	o__Coriobacteria	f__Coriobacteri	g__unclassified_f__Coriobacteriaceae	s__unclassified_f__Coriobacteriaceae
OTU55	p__Bacteroidet	c__Bacteroidia	o__Bacteroidales	f__Bacteroidale	g__norank_f__Bacteroidales_S24-7_group	s__uncultured_bacterium_g__norank_f__Bacteroidales_S24-7
OTU637	p__Firmicutes	c__Clostridia	o__Clostridiales	f__Ruminococc	g__Ruminococcaceae_UCG-014	s__uncultured_bacterium_g__Ruminococcaceae_UCG-014
OTU186	p__Firmicutes	c__Clostridia	o__Clostridiales	f__Lachnospira	g__Tyzzerella_3	s__uncultured_rumen_bacterium_g__Tyzzerella_3
OTU223	p__Firmicutes	c__Clostridia	o__Clostridiales	f__Lachnospira	g__unclassified_f__Lachnospiraceae	s__unclassified_f__Lachnospiraceae
OTU200	p__Actinobacte	c__Actinobacte	o__Coriobacteria	f__Coriobacteri	g__Enterorhabdus	s__uncultured_bacterium_g__Enterorhabdus
OTU452	p__Firmicutes	c__Clostridia	o__Clostridiales	f__Ruminococc	g__Ruminiclostridium_9	s__uncultured_bacterium_g__Ruminiclostridium_9
OTU667	p__Firmicutes	c__Clostridia	o__Clostridiales	f__Lachnospira	g__Lachnospiraceae_NK4A136_group	s__uncultured_Clostridiales_bacterium_g__Lachnospiraceae_
OTU8	p__Bacteroidet	c__Bacteroidia	o__Bacteroidales	f__Bacteroidale	g__norank_f__Bacteroidales_S24-7_group	s__uncultured_bacterium_g__norank_f__Bacteroidales_S24-7
OTU340	p__Bacteroidet	c__Bacteroidia	o__Bacteroidales	f__Prevotellace	g__Prevotellaceae_UCG-003	s__uncultured_bacterium_g__Prevotellaceae_UCG-003
OTU493	p__Firmicutes	c__Clostridia	o__Clostridiales	f__Lachnospira	g__Lachnospiraceae_NK4A136_group	s__Lachnospiraceae_bacterium_COE1
OTU689	p__Firmicutes	c__Clostridia	o__Clostridiales	f__Lachnospira	g__Coprococcus_1	s__unclassified_g__Coprococcus_1
OTU428	p__Firmicutes	c__Clostridia	o__Clostridiales	f__Ruminococc	g__unclassified_f__Ruminococcaceae	s__unclassified_f__Ruminococcaceae
OTU40	p__Firmicutes	c__Clostridia	o__Clostridiales	f__Clostridiales	g__norank_f__Clostridiales_vadinBB60_group	s__uncultured_bacterium_g__norank_f__Clostridiales_vadinE
OTU444	p__Firmicutes	c__Negativicut	o__Selenomonad	f__Veillonellac	g__Veillonella	s__unclassified_g__Veillonella
OTU296	p__Firmicutes	c__Clostridia	o__Clostridiales	f__Ruminococc	g__unclassified_f__Ruminococcaceae	s__unclassified_f__Ruminococcaceae
OTU107	p__Proteobacte	c__Betaproteob	o__Burkholderia	f__Alcaligenac	g__Parasutterella	s__Sutterellaceae_bacterium_YL45
OTU52	p__Bacteroidet	c__Bacteroidia	o__Bacteroidales	f__Bacteroidale	g__norank_f__Bacteroidales_S24-7_group	s__uncultured_bacterium_g__norank_f__Bacteroidales_S24-7
OTU58	p__Firmicutes	c__Clostridia	o__Clostridiales	f__Lachnospira	g__unclassified_f__Lachnospiraceae	s__unclassified_f__Lachnospiraceae
OTU97	p__Firmicutes	c__Erysipelotri	o__Erysipelotrich	f__Erysipelotric	g__Erysipelatoclostridium	s__uncultured_bacterium_g__Erysipelatoclostridium
OTU336	p__Proteobacte	c__Epsilonprot	o__Campylobact	f__Helicobacte	g__Helicobacter	s__Helicobacter_hepaticus
OTU41	p__Bacteroidet	c__Bacteroidia	o__Bacteroidales	f__Bacteroidale	g__norank_f__Bacteroidales_S24-7_group	s__uncultured_bacterium_g__norank_f__Bacteroidales_S24-7
OTU411	p__Tenericutes	c__Mollicutes	o__Anaeroplasm	f__Anaeroplasn	g__Anaeroplasma	s__uncultured_bacterium_g__Anaeroplasma
OTU225	p__Bacteroidet	c__Bacteroidia	o__Bacteroidales	f__Porphyromo	g__Parabacteroides	s__unclassified_g__Parabacteroides
OTU153	p__Firmicutes	c__Bacilli	o__Lactobacillal	f__Lactobacilla	g__Lactobacillus	s__Lactobacillus_ruminis
OTU224	p__Firmicutes	c__Clostridia	o__Clostridiales	f__Ruminococc	g__unclassified_f__Ruminococcaceae	s__unclassified_f__Ruminococcaceae
OTU139	p__Firmicutes	c__Clostridia	o__Clostridiales	f__Ruminococc	g__Ruminococcaceae_UCG-014	s__unclassified_g__Ruminococcaceae_UCG-014
OTU522	p__Bacteroidet	c__Bacteroidia	o__Bacteroidales	f__unclassified_	g__unclassified_o__Bacteroidales	s__unclassified_o__Bacteroidales
OTU582	p__Verrucomic	c__Verrucomic	o__Verrucomicr	f__Verrucomic	g__Akkermansia	s__Akkermansia_muciniphila
OTU389	p__Bacteroidet	c__Bacteroidia	o__Bacteroidales	f__Bacteroidale	g__norank_f__Bacteroidales_S24-7_group	s__uncultured_bacterium_g__norank_f__Bacteroidales_S24-7
OTU288	p__Deferribact	c__Deferribact	o__Deferribacter	f__Deferribacte	g__Mucispirillum	s__Mucispirillum_schaedleri
OTU286	p__Firmicutes	c__Bacilli	o__Lactobacillal	f__Streptococca	g__Streptococcus	s__Streptococcus_danieliae
OTU242	p__Firmicutes	c__Clostridia	o__Clostridiales	f__Lachnospira	g__unclassified_f__Lachnospiraceae	s__unclassified_f__Lachnospiraceae
OTU591	p__Firmicutes	c__Clostridia	o__Clostridiales	f__Lachnospira	g__norank_f__Lachnospiraceae	s__unclassified_g__norank_f__Lachnospiraceae

OTU106	p__Tenericutes	c__Mollicutes	o__Mollicutes_R	f__norank_o__l	g__norank_o__Mollicutes_RF9		s__uncultured_bacterium_g__norank_o__Mollicutes_RF9
OTU128	p__Bacteroidet	c__Bacteroidia	o__Bacteroidales	f__Prevotellace	g__Prevotellaceae_UCG-001		s__uncultured_Bacteroidales_bacterium_g__Prevotellaceae_U
OTU190	p__Bacteroidet	c__Bacteroidia	o__Bacteroidales	f__Prevotellace	g__Prevotellaceae_UCG-001		s__uncultured_Bacteroidales_bacterium_g__Prevotellaceae_U
OTU540	p__Firmicutes	c__Clostridia	o__Clostridiales	f__Lachnospira	g__Roseburia		s__Clostridium_sp._Clone-44
OTU86	p__Actinobacte	c__Actinobacte	o__Coriobacteria	f__Coriobacteri	g__unclassified_f__Coriobacteriaceae		s__unclassified_f__Coriobacteriaceae
OTU463	p__Firmicutes	c__Clostridia	o__Clostridiales	f__Lachnospira	g__norank_f__Lachnospiraceae		s__uncultured_bacterium_g__norank_f__Lachnospiraceae
OTU17	p__Bacteroidet	c__Bacteroidia	o__Bacteroidales	f__Bacteroidale	g__norank_f__Bacteroidales_S24-7_group		s__uncultured_bacterium_g__norank_f__Bacteroidales_S24-7
OTU10	p__Bacteroidet	c__Bacteroidia	o__Bacteroidales	f__Bacteroidale	g__norank_f__Bacteroidales_S24-7_group		s__uncultured_Bacteroidales_bacterium_g__norank_f__Bacte
OTU608	p__Bacteroidet	c__Bacteroidia	o__Bacteroidales	f__Bacteroidale	g__norank_f__Bacteroidales_S24-7_group		s__uncultured_Barnesiella_sp._g__norank
OTU205	p__Firmicutes	c__Clostridia	o__Clostridiales	f__Ruminococc	g__Anaerotruncus		s__uncultured_bacterium_g__Anaerotruncus
OTU327	p__Firmicutes	c__Clostridia	o__Clostridiales	f__Ruminococc	g__norank_f__Ruminococcaceae		s__[Clostridium]_leptum_g__norank
OTU232	p__Bacteroidet	c__Bacteroidia	o__Bacteroidales	f__Bacteroidac	g__Bacteroides		s__Bacteroides_plebeius
OTU37	p__Firmicutes	c__Clostridia	o__Clostridiales	f__Ruminococc	g__Ruminococcaceae_UCG-014		s__unclassified_g__Ruminococcaceae_UCG-014
OTU579	p__Bacteroidet	c__Bacteroidia	o__Bacteroidales	f__Bacteroidale	g__norank_f__Bacteroidales_S24-7_group		s__uncultured_bacterium_g__norank_f__Bacteroidales_S24-7
OTU275	p__Bacteroidet	c__Bacteroidia	o__Bacteroidales	f__Prevotellace	g__Alloprevotella		s__uncultured_Bacteroidales_bacterium_g__Alloprevotella
OTU19	p__Proteobacte	c__Alphaprote	o__Rhodospirilla	f__Rhodospirill	g__norank_f__Rhodospirillaceae		s__gut_metagenome_g__norank_f__Rhodospirillaceae
OTU45	p__Actinobacte	c__Actinobacte	o__Coriobacteria	f__Coriobacteri	g__Senegalimassilia		s__unclassified_g__Senegalimassilia
OTU206	p__Bacteroidet	c__Bacteroidia	o__Bacteroidales	f__Bacteroidale	g__norank_f__Bacteroidales_S24-7_group		s__uncultured_bacterium_g__norank_f__Bacteroidales_S24-7
OTU652	p__Firmicutes	c__Clostridia	o__Clostridiales	f__Lachnospira	g__unclassified_f__Lachnospiraceae		s__unclassified_f__Lachnospiraceae
OTU460	p__Tenericutes	c__Mollicutes	o__Mollicutes_R	f__norank_o__l	g__norank_o__Mollicutes_RF9		s__uncultured_Firmicutes_bacterium_g__norank_o__Mollicu
OTU503	p__Bacteroidet	c__Bacteroidia	o__Bacteroidales	f__Bacteroidac	g__Bacteroides		s__unclassified_g__Bacteroides
OTU707	p__Bacteroidet	c__Bacteroidia	o__Bacteroidales	f__Bacteroidale	g__norank_f__Bacteroidales_S24-7_group		s__uncultured_Bacteroidales_bacterium_g__norank_f__Bacte
OTU465	p__Firmicutes	c__Clostridia	o__Clostridiales	f__Lachnospira	g__Lachnospiraceae_NK4A136_group		s__unclassified_g__Lachnospiraceae_NK4A136_group
OTU25	p__Cyanobacte	c__Cyanobacte	o__Gastranaerop	f__norank_o__(	g__norank_o__Gastranaerophilales		s__uncultured_bacterium_g__norank_o__Gastranaerophilales
OTU277	p__Firmicutes	c__Clostridia	o__Clostridiales	f__Lachnospira	g__unclassified_f__Lachnospiraceae		s__unclassified_f__Lachnospiraceae
OTU87	p__Cyanobacte	c__Cyanobacte	o__Gastranaerop	f__norank_o__(	g__norank_o__Gastranaerophilales		s__uncultured_bacterium_g__norank_o__Gastranaerophilales
OTU104	p__Bacteroidet	c__Bacteroidia	o__Bacteroidales	f__Bacteroidale	g__norank_f__Bacteroidales_S24-7_group		s__uncultured_bacterium_g__norank_f__Bacteroidales_S24-7
OTU557	p__Bacteroidet	c__Bacteroidia	o__Bacteroidales	f__Bacteroidale	g__norank_f__Bacteroidales_S24-7_group		s__unclassified_g__norank_f__Bacteroidales_S24-7_group
OTU643	p__Firmicutes	c__Clostridia	o__Clostridiales	f__Ruminococc	g__Ruminococcaceae_UCG-014		s__unclassified_g__Ruminococcaceae_UCG-014
OTU545	p__Firmicutes	c__Clostridia	o__Clostridiales	f__Lachnospira	g__Lachnospiraceae_NK4A136_group		s__unclassified_g__Lachnospiraceae_NK4A136_group
OTU187	p__Bacteroidet	c__Bacteroidia	o__Bacteroidales	f__Rikenellacea	g__Rikenellaceae_RC9_gut_group		s__unclassified_g__Rikenellaceae_RC9_gut_group
OTU368	p__Firmicutes	c__Clostridia	o__Clostridiales	f__Family_XIII	g__Family_XIII_AD3011_group		s__unclassified_g__Family_XIII_AD3011_group
OTU419	p__Firmicutes	c__Clostridia	o__Clostridiales	f__Lachnospira	g__unclassified_f__Lachnospiraceae		s__unclassified_f__Lachnospiraceae
OTU226	p__Bacteroidet	c__Bacteroidia	o__Bacteroidales	f__Bacteroidale	g__norank_f__Bacteroidales_S24-7_group		s__uncultured_bacterium_g__norank_f__Bacteroidales_S24-7
OTU650	p__Bacteroidet	c__Bacteroidia	o__Bacteroidales	f__Bacteroidale	g__norank_f__Bacteroidales_S24-7_group		s__uncultured_bacterium_g__norank_f__Bacteroidales_S24-7
OTU619	p__Cyanobacte	c__Cyanobacte	o__Gastranaerop	f__norank_o__(	g__norank_o__Gastranaerophilales		s__uncultured_bacterium_g__norank_o__Gastranaerophilales
OTU710	p__Firmicutes	c__Clostridia	o__Clostridiales	f__Ruminococc	g__Ruminiclostridium_5		s__uncultured_bacterium_g__Ruminiclostridium_5
OTU455	p__Firmicutes	c__Negativicut	o__Selenomonad	f__Veillonellac	g__Veillonella		s__unclassified_g__Veillonella
OTU596	p__Firmicutes	c__Clostridia	o__Clostridiales	f__Clostridiales	g__norank_f__Clostridiales_vadinBB60_group		s__unclassified_g__norank_f__Clostridiales_vadinBB60_grou

OTU244	p__Firmicutes	c__Clostridia	o__Clostridiales	f__Ruminococc	g__norank_f__Ruminococcaceae	s__unclassified_g__norank_f__Ruminococcaceae
OTU196	p__Bacteroidete	c__Bacteroidia	o__Bacteroidales	f__Rikenellacea	g__Alistipes	s__uncultured_bacterium_g__Alistipes
OTU458	p__Tenericutes	c__Mollicutes	o__Mollicutes_R	f__norank_o__l	g__norank_o__Mollicutes_RF9	s__uncultured_bacterium_g__norank_o__Mollicutes_RF9
OTU614	p__Bacteroidete	c__Bacteroidia	o__Bacteroidales	f__Bacteroidale	g__norank_f__Bacteroidales_S24-7_group	s__uncultured_bacterium_g__norank_f__Bacteroidales_S24-7
OTU33	p__Tenericutes	c__Mollicutes	o__Mollicutes_R	f__norank_o__l	g__norank_o__Mollicutes_RF9	s__uncultured_bacterium_g__norank_o__Mollicutes_RF9
OTU587	p__Bacteroidete	c__Bacteroidia	o__Bacteroidales	f__Bacteroidale	g__norank_f__Bacteroidales_S24-7_group	s__uncultured_Bacteroidales_bacterium_g__norank_f__Bacte
OTU16	p__Tenericutes	c__Mollicutes	o__Mollicutes_R	f__norank_o__l	g__norank_o__Mollicutes_RF9	s__unclassified_g__norank_o__Mollicutes_RF9
OTU457	p__Firmicutes	c__Clostridia	o__Clostridiales	f__Lachnospira	g__norank_f__Lachnospiraceae	s__unclassified_g__norank_f__Lachnospiraceae
OTU488	p__Bacteroidete	c__Bacteroidia	o__Bacteroidales	f__Bacteroidale	g__norank_f__Bacteroidales_S24-7_group	s__uncultured_bacterium_g__norank_f__Bacteroidales_S24-7
OTU386	p__Bacteroidete	c__Bacteroidia	o__Bacteroidales	f__Prevotellace	g__Prevotellaceae_UCG-003	s__uncultured_bacterium_g__Prevotellaceae_UCG-003
OTU528	p__Firmicutes	c__Erysipelotri	o__Erysipelotrich	f__Erysipelotric	g__Erysipelatoclostridium	s__unclassified_g__Erysipelatoclostridium
OTU279	p__Firmicutes	c__Bacilli	o__Lactobacillale	f__Streptococca	g__Lactococcus	s__uncultured_bacterium_g__Lactococcus
OTU709	p__Firmicutes	c__Clostridia	o__Clostridiales	f__Ruminococc	g__Ruminiclostridium_6	s__uncultured_bacterium_g__Ruminiclostridium_6
OTU7	p__Tenericutes	c__Mollicutes	o__Mollicutes_R	f__norank_o__l	g__norank_o__Mollicutes_RF9	s__uncultured_bacterium_g__norank_o__Mollicutes_RF9
OTU24	p__Bacteroidete	c__Bacteroidia	o__Bacteroidales	f__Bacteroidale	g__norank_f__Bacteroidales_S24-7_group	s__uncultured_bacterium_g__norank_f__Bacteroidales_S24-7
OTU56	p__Firmicutes	c__Clostridia	o__Clostridiales	f__Ruminococc	g__Anaerotruncus	s__[Clostridium]_leptum_g__Anaerotruncus
OTU116	p__Actinobacte	c__Actinobacte	o__Coriobacteria	f__Coriobacteri	g__Enterorhabdus	s__uncultured_bacterium_g__Enterorhabdus
OTU75	p__Firmicutes	c__Clostridia	o__Clostridiales	f__Ruminococc	g__unclassified_f__Ruminococcaceae	s__unclassified_f__Ruminococcaceae
OTU413	p__Cyanobacte	c__Cyanobacte	o__Gastranaerop	f__norank_o__(	g__norank_o__Gastranaerophilales	s__uncultured_bacterium_g__norank_o__Gastranaerophilales
OTU181	p__Firmicutes	c__Clostridia	o__Clostridiales	f__Family_XIII	g__Family_XIII_AD3011_group	s__unclassified_g__Family_XIII_AD3011_group
OTU603	p__Firmicutes	c__Clostridia	o__Clostridiales	f__Family_XIII	g__[Eubacterium]_brachy_group	s__uncultured_bacterium_g__[Eubacterium]_brachy_group
OTU67	p__Firmicutes	c__Clostridia	o__Clostridiales	f__Ruminococc	g__Ruminococcaceae_UCG-009	s__unclassified_g__Ruminococcaceae_UCG-009
OTU502	p__Firmicutes	c__Clostridia	o__Clostridiales	f__Lachnospira	g__Lachnospiraceae_NK4A136_group	s__uncultured_bacterium_g__Lachnospiraceae_NK4A136_gr
OTU357	p__Firmicutes	c__Erysipelotri	o__Erysipelotrich	f__Erysipelotric	g__unclassified_f__Erysipelotrichaceae	s__unclassified_f__Erysipelotrichaceae
OTU124	p__Firmicutes	c__Clostridia	o__Clostridiales	f__Lachnospira	g__norank_f__Lachnospiraceae	s__Lachnospiraceae_bacterium_A2
OTU160	p__Firmicutes	c__Clostridia	o__Clostridiales	f__Ruminococc	g__unclassified_f__Ruminococcaceae	s__unclassified_f__Ruminococcaceae
OTU479	p__Firmicutes	c__Clostridia	o__Clostridiales	f__Lachnospira	g__unclassified_f__Lachnospiraceae	s__unclassified_f__Lachnospiraceae
OTU34	p__Bacteroidete	c__Bacteroidia	o__Bacteroidales	f__Bacteroidale	g__norank_f__Bacteroidales_S24-7_group	s__uncultured_bacterium_g__norank_f__Bacteroidales_S24-7
OTU438	p__Firmicutes	c__Clostridia	o__Clostridiales	f__Clostridiales	g__norank_f__Clostridiales_vadinBB60_group	s__unclassified_g__norank_f__Clostridiales_vadinBB60_grou
OTU666	p__Firmicutes	c__Clostridia	o__Clostridiales	f__Clostridiales	g__norank_f__Clostridiales_vadinBB60_group	s__unclassified_g__norank_f__Clostridiales_vadinBB60_grou
OTU233	p__Firmicutes	c__Clostridia	o__Clostridiales	f__Lachnospira	g__Acetatifactor	s__uncultured_bacterium_g__Acetatifactor
OTU392	p__Firmicutes	c__Clostridia	o__Clostridiales	f__Lachnospira	g__Roseburia	s__unclassified_g__Roseburia
OTU251	p__Actinobacte	c__Actinobacte	o__Coriobacteria	f__Coriobacteri	g__Enterorhabdus	s__uncultured_bacterium_g__Enterorhabdus
OTU484	p__Firmicutes	c__Clostridia	o__Clostridiales	f__Lachnospira	g__unclassified_f__Lachnospiraceae	s__unclassified_f__Lachnospiraceae
OTU515	p__Bacteroidete	c__Bacteroidia	o__Bacteroidales	f__Rikenellacea	g__Rikenellaceae_RC9_gut_group	s__uncultured_bacterium_g__Rikenellaceae_RC9_gut_group
OTU433	p__Firmicutes	c__Clostridia	o__Clostridiales	f__Lachnospira	g__Acetatifactor	s__unclassified_g__Acetatifactor
OTU241	p__Firmicutes	c__Clostridia	o__Clostridiales	f__Lachnospira	g__unclassified_f__Lachnospiraceae	s__unclassified_f__Lachnospiraceae
OTU229	p__Firmicutes	c__Clostridia	o__Clostridiales	f__Ruminococc	g__Anaerotruncus	s__uncultured_bacterium_g__Anaerotruncus
OTU189	p__Bacteroidete	c__Bacteroidia	o__Bacteroidales	f__Bacteroidale	g__norank_f__Bacteroidales_S24-7_group	s__uncultured_bacterium_g__norank_f__Bacteroidales_S24-7

OTU38	p__Firmicutes	c__Erysipelotri	o__Erysipelotricl	f__Erysipelotric	g__[Clostridium]_innocuum_group	s__uncultured_bacterium_g__[Clostridium]_innocuum_group
OTU406	p__Actinobacte	c__Actinobacte	o__Coriobacteria	f__Coriobacteri	g__Enterorhabdus	s__uncultured_bacterium_g__Enterorhabdus
OTU118	p__Firmicutes	c__Clostridia	o__Clostridiales	f__Lachnospira	g__Roseburia	s__unclassified_g__Roseburia
OTU168	p__Proteobacte	c__Deltaproteo	o__Desulfovibric	f__Desulfovibri	g__Desulfovibrio	s__uncultured_Desulfovibrionaceae_bacterium
OTU481	p__Firmicutes	c__Clostridia	o__Clostridiales	f__Lachnospira	g__Lachnospiraceae_NK4A136_group	s__unclassified_g__Lachnospiraceae_NK4A136_group
OTU85	p__Actinobacte	c__Actinobacte	o__Coriobacteria	f__Coriobacteri	g__norank_f__Coriobacteriaceae	s__gut_metagenome_g__norank
OTU711	p__Firmicutes	c__Clostridia	o__Clostridiales	f__Lachnospira	g__Lachnospiraceae_UCG-006	s__uncultured_bacterium_g__Lachnospiraceae_UCG-006
OTU414	p__Tenericutes	c__Mollicutes	o__Mollicutes_R	f__norank_o__l	g__norank_o__Mollicutes_RF9	s__uncultured_bacterium_g__norank_o__Mollicutes_RF9
OTU633	p__Bacteroidet	c__Bacteroidia	o__Bacteroidales	f__Bacteroidale	g__norank_f__Bacteroidales_S24-7_group	s__unclassified_g__norank_f__Bacteroidales_S24-7_group
OTU653	p__Firmicutes	c__Clostridia	o__Clostridiales	f__Lachnospira	g__unclassified_f__Lachnospiraceae	s__unclassified_f__Lachnospiraceae
OTU482	p__Bacteroidet	c__Bacteroidia	o__Bacteroidales	f__Bacteroidale	g__norank_f__Bacteroidales_S24-7_group	s__uncultured_bacterium_g__norank_f__Bacteroidales_S24-7
OTU602	p__Firmicutes	c__Clostridia	o__Clostridiales	f__Lachnospira	g__Roseburia	s__uncultured_Clostridiales_bacterium_g__Roseburia
OTU671	p__Firmicutes	c__Clostridia	o__Clostridiales	f__Lachnospira	g__Roseburia	s__Eubacterium_sp._14-2

Abbreviations: OTU: operational taxonomic unit; CSDS: chronic social defeated stress.

idiales_vadinBB60_group
