# Supplementary material for: Altered Fecal Metabolites and Colonic Glycerophospholipids Were Associated With Abnormal Composition of Gut Microbiota in a Depression Model of Mice
Source: Front Neurosci. 2021 Jul 19;15:701355. doi: 10.3389/fnins.2021.701355 (PMC8326978; doi:10.3389/fnins.2021.701355)
Supplement: Supplementary file 2 [file Data_Sheet_2.PDF]

Supplementary Table S2. Differential fecal metabolites identified from the control and CSDS mice.

NO.	Metabolites	Classification	RT(min)	Ratio (CSDS/CON)	*P-value	**VIP	HMDB ID
1	Dodecane	Alkanes	7.865	1.35882321	0.02959697	1.07088	HMDB0031444
2	Heptadecane	Alkanes	16.318	1.561718389	0.00139399	1.40624	/
3	Tricosane	Alkanes	24.922	2.148423469	0.00780336	1.6877	/
4	Methionine	Amino acids and derivatives	17.933	1.968045062	0.0413865	1.0397	HMDB0000696
5	3,5-Diiodo-L-tyrosine	Amino acids and derivatives	11.188	3.233162901	0.00046925	2.49569	HMDB0003474
6	4-Aminobutyric acid (GABA)	Neurotransmitter	14.562	1.527296492	0.00040917	1.21962	HMDB0000112
7	2-Hydroxyglutaramic acid	Organic compound	16.345	1.500520727	0.00192958	1.07293	/
8	3-Methyl-2-oxovaleric acid	Organic compound	7.489	2.645026308	0.00961521	1.22894	HMDB0000491
9	Hydroxybenzoic acid	Organic compound	16.948	0.468256149	0.04163392	1.01977	HMDB0000500
10	Arachidic acid (C20_0)	Saturated fatty acids	26.655	1.665145757	0.02816732	1.18624	HMDB0002212
11	Behenic acid (C22_0)	Saturated fatty acids	29.662	1.624569824	0.03810448	1.23946	HMDB0000944
12	Margaric acid (C17_0)	Saturated fatty acids	22.921	1.81188208	0.02553855	1.19169	HMDB0002259
13	Myristic acid (C14_0)	Saturated fatty acids	17.511	1.820735061	0.02814069	1.05114	HMDB0000806
14	Nonadecanoic acid (C19_0)	Saturated fatty acids	25.193	1.858476751	0.0183678	1.2378	HMDB0000772
15	Stearic acid (C18_0)	Saturated fatty acids	23.992	1.658597405	0.023294	1.04964	HMDB0000827
16	Tricosanoic acid (C23_0)	Saturated fatty acids	31.161	1.703789864	0.04461954	1.05841	HMDB0001160
17	10-Heptadecenoic acid (C17_1n-7t)	Unsaturated fatty acids	22.861	2.138850757	0.00664333	1.11294	/
18	10-Pentadecenoic acid (C15_1n-5c)	Unsaturated fatty acids	18.802	1.408439091	0.00489725	1.0407	/
19	Conjugated linoleic acid (C18_2n-9,11c)	Unsaturated fatty acids	24.837	2.64766408	0.03634775	1.24582	HMDB0005047
20	Gondoic acid (C20_1n-9c)	Unsaturated fatty acids	26.406	1.907549171	0.03702992	1.11846	HMDB0002231

Abbreviation: RT: retention time.

*p-values were derived from Student's t-test.

**variable importance in the projection (VIP) was obtained from PLS-DA with a threshold of 1.0.
