# Supplementary material for: Altered Fecal Metabolites and Colonic Glycerophospholipids Were Associated With Abnormal Composition of Gut Microbiota in a Depression Model of Mice
Source: Front Neurosci. 2021 Jul 19;15:701355. doi: 10.3389/fnins.2021.701355 (PMC8326978; doi:10.3389/fnins.2021.701355)
Supplement: Supplementary file 3 [file Data_Sheet_3.PDF]

Supplementary Table S3. Lipid categories and classes detected in the lipidomic profiling.

Categories	Lipid class	Abbreviation
Fatty Acyls	Acyl Carnitine	AcCa
	Fatty acid	FA
	(O-acyl)-1-hydroxy fatty acid	OAHFA
	wax exters	WE
Glycerolipids	Diglyceride	DG
	Monoglyceride	MG
	Triglyceride	TG
Glycerophospholipids	Cardiolipin	CL
	Lysophosphatidylcholine	LPC
	Lysophosphatidylethanolamine	LPE
	Lysophosphatidylglycerol	LPG
	Lysophosphatidylinositol	LPI
	Lysophosphatidylserine	LPS
	Phosphatidic acid	PA
	Phosphatidylcholine	PC
	Phosphatidylethanolamine	PE
	Phosphatidylglycerol	PG
	Phosphatidylinositol	PI
	Phosphatidylserine	PS
Prenol lipids	Coenzyme	Co
Sphingolipids	Ceramides	Cer
	Glucosylceramide	CerG1
	Simple Glc series	CerG3GNAc1
	Gangliosides	GM3
	phytosphingosine	phSM
	Sphingomyelin	SM
	Sphingosine	So
Saccharolipids	Monogalactosyldiacylglycerol	MGDG
	Monogalactosylmonoacylglycerol	MGMG
	Sulfoquinovosyldiacylglycerol	SQDG
