# Supplementary material for: Altered Fecal Metabolites and Colonic Glycerophospholipids Were Associated With Abnormal Composition of Gut Microbiota in a Depression Model of Mice
Source: Front Neurosci. 2021 Jul 19;15:701355. doi: 10.3389/fnins.2021.701355 (PMC8326978; doi:10.3389/fnins.2021.701355)
Supplement: Supplementary file 4 [file Data_Sheet_4.PDF]

Supplementary Table S4. Differentially expressed lipids in colon identified between the control and CSDS mice.

NO.	LipidIon	Class	IonFormula	CalMz	RT (min)	Ratio (CSDS/CON)	*P-value	** VIP	Ion Type
1	LPC(18:0)+H	LPC	C26 H55 O7 N1 P1	524.3710685	4.499700142	2.097	0.014393	7.28562	POS
2	PC(34:2p)+H	PC	C42 H81 O7 N1 P1	742.5745185	11.89966565	1.6514	0.024186	3.58639	POS
3	PC(33:2)+H	PC	C41 H79 O8 N1 P1	744.5537835	12.73	1.5571	0.026617	2.77767	POS
4	PC(35:4)+H	PC	C43 H79 O8 N1 P1	768.5537835	12.52682286	0.83223	0.026076	3.02853	POS
5	PC(18:0p/18:1)+H	PC	C44 H87 O7 N1 P1	772.6214685	13.07314813	1.4915	0.047817	2.09599	POS
6	PC(36:2e)+H	PC	C44 H87 O7 N1 P1	772.6214685	13.74587737	1.6924	0.0079443	1.04571	POS
7	PC(37:2)+H	PC	C45 H87 O8 N1 P1	800.6163835	12.74007715	1.833	0.014976	1.45458	POS
8	PE(16:0p/18:2)+H	PE	C39 H75 O7 N1 P1	700.5275685	12.22445989	1.5014	0.0071917	1.6982	POS
9	PE(16:0p/20:5)+H	PE	C41 H73 O7 N1 P1	722.5119185	11.3489034	1.2673	0.018323	1.57083	POS
10	PE(18:0p/18:2)+H	PE	C41 H79 O7 N1 P1	728.5588685	13.26641155	1.7752	0.014444	2.22541	POS
11	PE(18:0p/18:1)+H	PE	C41 H81 O7 N1 P1	730.5745185	14.12163565	1.326	0.020652	2.11964	POS
12	PE(18:0p/20:3)+H	PE	C43 H81 O7 N1 P1	754.5745185	13.49049691	1.3658	0.027685	1.243	POS
13	PE(20:0p/18:2)+H	PE	C43 H83 O7 N1 P1	756.5901685	14.3679061	2.7718	0.0014819	1.72428	POS
14	PE(18:0/18:1)+Na	PE	C41 H80 O8 N1 P1 Na1	768.5513785	12.452	2.6196	0.0018665	6.92601	POS
15	SM(d40:1)+H	SM	C45 H92 O6 N2 P1	787.6687525	14.30168267	0.73049	0.040094	1.84212	POS
16	SM(d42:3)+H	SM	C47 H92 O6 N2 P1	811.6687525	13.27688946	0.83555	0.035384	1.87659	POS
17	CerG1(d20:0+pO/24:0+O)+H	CerG1	C50 H100 O10 N1	874.7341755	16.24026079	1.5764	0.039062	1.08992	POS
18	DG(36:2)+NH4	DG	C39 H76 O5 N1	638.5718005	13.46	0.30213	0.020728	1.36992	POS
19	TG(18:1/18:2/18:2)+NH4	TG	C57 H104 O6 N1	898.7858155	22.39576482	0.40756	0.011865	1.55187	POS
20	PC(16:0/16:0)+HCOO	PC	C41 H81 O10 N1 P1	778.5603605	12.12223731	0.86143	0.016504	5.52837	NEG
21	PC(16:0p/18:2)+HCOO	PC	C43 H81 O9 N1 P1	786.5654455	11.91075447	1.6603	0.0097555	2.61031	NEG
22	PC(16:0e/18:2)+HCOO	PC	C43 H83 O9 N1 P1	788.5810955	12.06224579	1.3188	0.049159	1.73539	NEG
23	PC(16:0/20:4)+HCOO	PC	C45 H81 O10 N1 P1	826.5603605	10.72897206	1.2436	0.018369	2.60717	NEG
24	PC(18:1/18:1)+HCOO	PC	C45 H85 O10 N1 P1	830.5916605	12.36331015	1.3748	0.024915	7.59457	NEG
25	PE(16:0/18:1)-H	PE	C39 H75 O8 N1 P1	716.5235805	12.49966107	0.77724	0.019684	3.06146	NEG
26	PE(36:3p)-H	PE	C41 H75 O7 N1 P1	724.5286655	12.21591645	1.3608	0.011381	2.53289	NEG
27	PE(18:0/18:2)-H	PE	C41 H77 O8 N1 P1	742.5392305	12.58416542	1.3532	0.029908	5.07278	NEG
28	PE(16:0p/22:5)-H	PE	C43 H75 O7 N1 P1	748.5286655	12.36897186	1.2891	0.031743	1.84576	NEG
29	PE(38:2p)-H	PE	C43 H81 O7 N1 P1	754.5756155	14.34999335	1.8086	0.027548	2.62766	NEG
30	PE(20:0p/18:1)-H	PE	C43 H83 O7 N1 P1	756.5912655	15.28191463	1.3155	0.047643	1.36023	NEG
31	PE(18:0p/22:6)-H	PE	C45 H77 O7 N1 P1	774.5443155	12.7129627	1.1522	0.043741	2.62566	NEG
32	PE(20:0p/20:5)-H	PE	C45 H79 O7 N1 P1	776.5599655	13.39987175	1.2402	0.040386	1.06948	NEG
33	PI(18:0/20:3)-H	PI	C47 H84 O13 N0 P1	887.5655065	11.84557614	0.63674	0.0016886	2.09205	NEG
34	PI(16:0/20:3)-H	PI	C45 H80 O13 N0 P1	859.5342065	10.84245634	0.584	0.025959	1.07702	NEG
35	PS(18:0/22:4)-H	PS	C46 H81 O10 N1 P1	838.5603605	12.01253143	0.75293	0.036873	2.84725	NEG
36	PG(22:6/22:6)-H	PG	C50 H74 O10 N0 P1	865.5025115	8.086428683	1.278	0.027752	1.07756	NEG

Abbreviation: POS: positive mode; NEG: negative mode.

*p-values were derived from Student's t-test.

**variable importance in the projection (VIP) was obtained from PLS-DA with a threshold of 1.0.
