# Supplementary material for: Altered Fecal Metabolites and Colonic Glycerophospholipids Were Associated With Abnormal Composition of Gut Microbiota in a Depression Model of Mice
Source: Front Neurosci. 2021 Jul 19;15:701355. doi: 10.3389/fnins.2021.701355 (PMC8326978; doi:10.3389/fnins.2021.701355)
Supplement: Supplementary file 5 [file Image_1.pdf]

Supplementary Figures

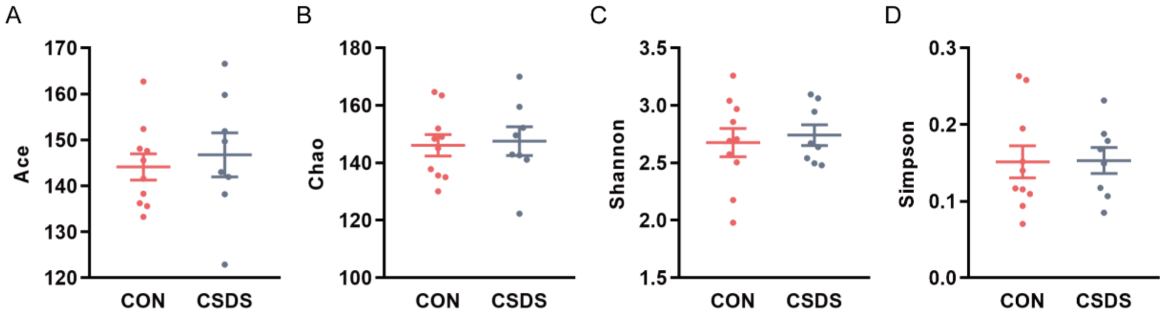

Supplementary Figure 1. No significance was found between the control and CSDS mice in the α-diversity indices of guace, (B) chao, (C) shannon and (D) simpson.

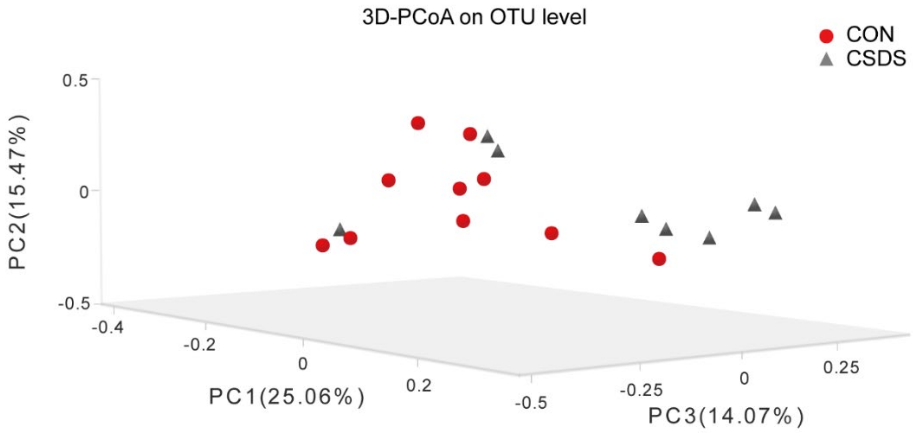

Supplementary Figure 2. The 3D-PCoA plot revealed that there was a clear separation between the control and CSDS mice in the gut microbiota composition.

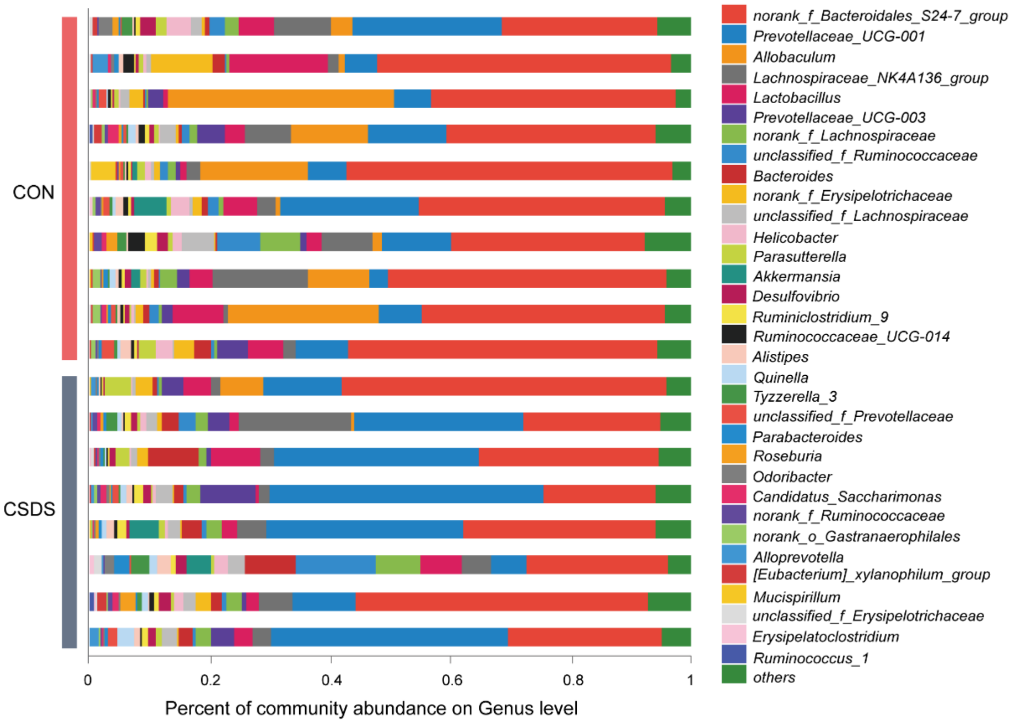

Supplementary Figure 3. The bar plot illustrated the relative abundance of gut microbiota community between the control and CSDS mice on genus level. The gut microbiome was mainly composed of 33 genera.

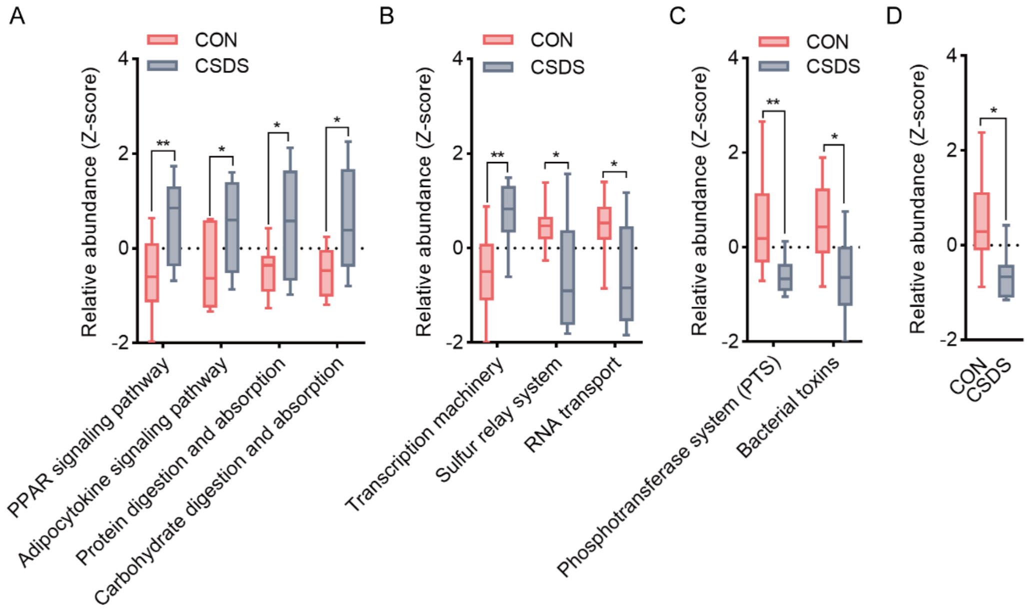

Supplementary Figure 4. Histograms of significantly altered functional category in the (A) Organismal Systems, (B) Genetic Information Processing, (C) Environmental Information Processing and (D) Human Diseases.

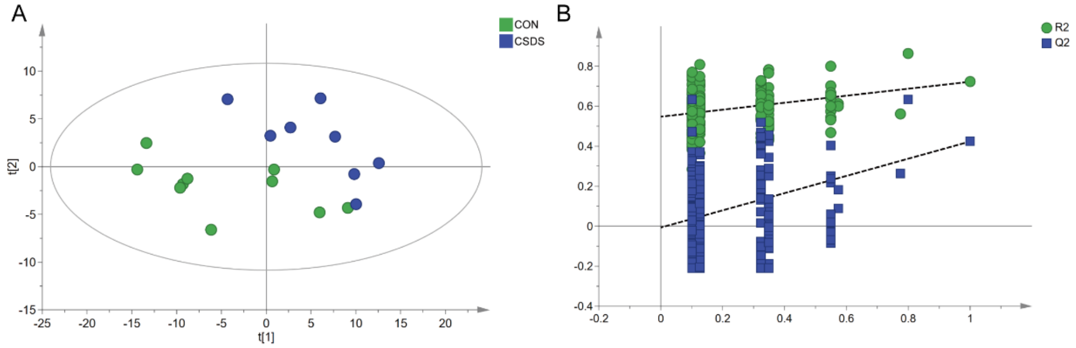

Supplementary Figure 5. (A) The PLS-DA model revealed a clear distinction between the metabolites in the control and CSDS groups (R2Y = 0.722, Q2 = 0.423) and (B) the permutation plot indicated that the original PLS-DA model was valid and not over-fitted (R2 = 0. 553, Q2 = −0.024).

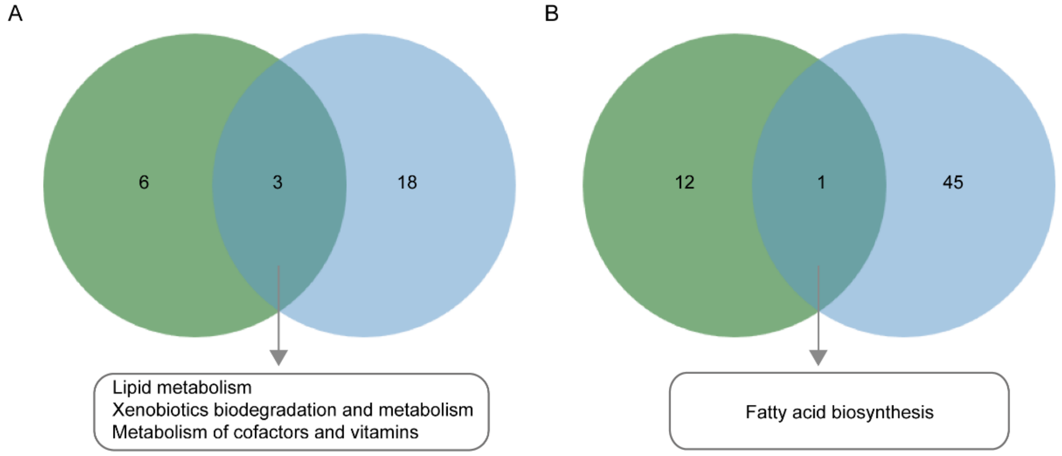

Supplementary Figure 6. (A) The venn plot revealed that Lipid metabolism, Xenobiotics biodegradation and metabolism and Metabolism of cofactors and vitamins were the overlapping categories of the second level KEGG pathways of gut microbiome and metabolome. (B) The venn plot revealed that “Fatty acid biosynthesis” was the only overlapping category in the third level KEGG pathways.

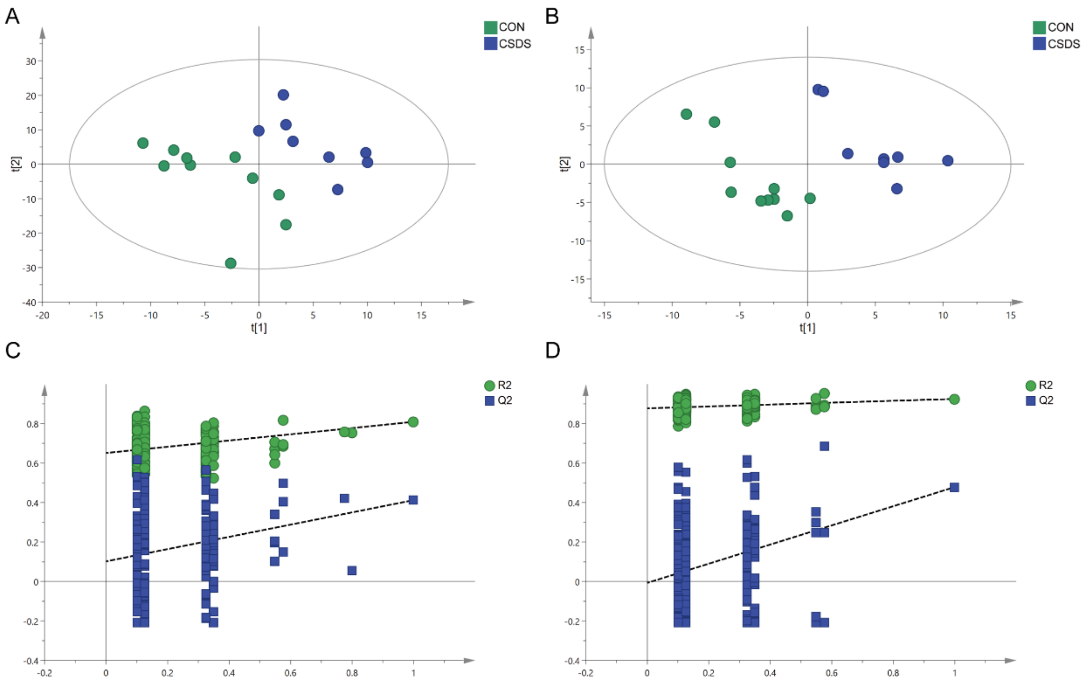

Supplementary Figure 7. (A) In the positive mode, the PLS-DA plot showed that the CSDS mice could be obviously separated from the control with no overlap (R2Y = 0.809, Q2 = 0.412) and (C) the permutation plot indicated that the original PLS-DA model was valid and not over-fitted (R2 = 0. 649, Q2 = 0.100). (B) In the negative mode, the PLS-DA plot indicated a distinct separation between the two groups (R2Y = 0.926, Q2 = 0.479) and (D) the permutation plot showed that the original model was valid and not over-fitted (R2 = 0. 877, Q2 = -0.008).

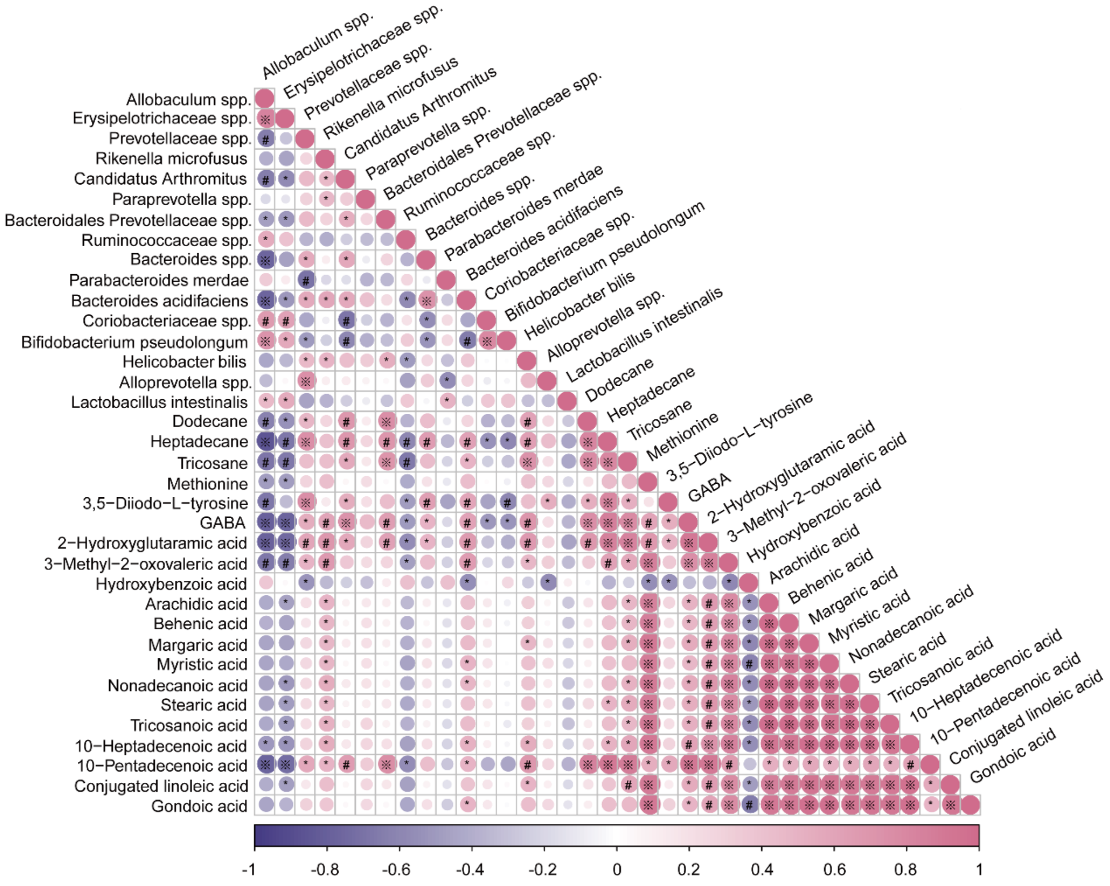

Supplementary Figure 8. Spearman’s rank correlation coefficient between the differential bacteria species and fecal metabolites. Red represents positive correlation; blue represents negative correlation. *$p < 0.05$, #$p < 0.01$, ※$p < 0.001$.

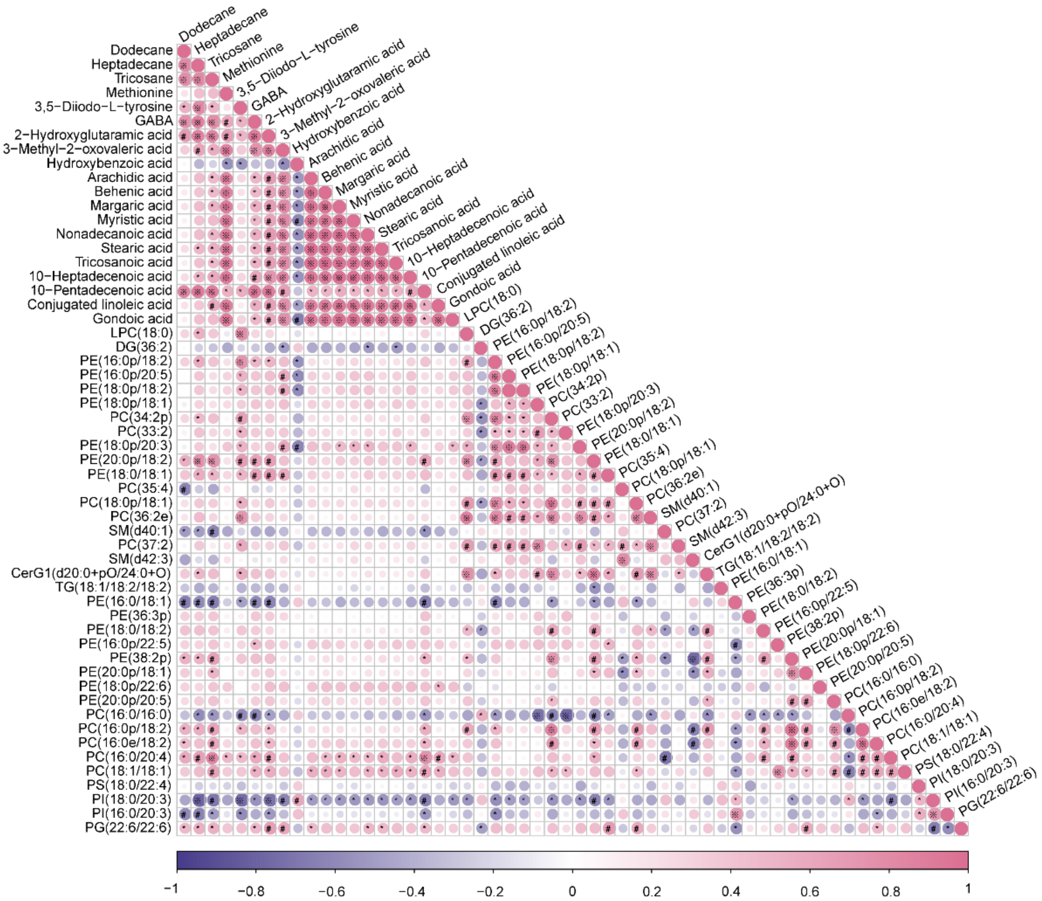

Supplementary Figure 9. Spearman's rank correlation coefficient between the differential fecal metabolites and colonic lipids. Red represents positive correlation; blue represents negative correlation. *$p < 0.05$, #$p < 0.01$, ※$p < 0.001$.

Supplementary Methods

1. CD1 screening protocol

Prior to CSDS procedure, CD1 mice were screened for aggressor candidates on three consecutive days and mice meeting the following criteria were selected(Golden et al. 2011, Wang et al. 2016): (i) a latency of attack less than one min and (ii) attacking for at least two consecutive days. Subsequently, these selected candidates were arranged to reside in the fighting cage for acclimatization. CSDS procedure was started within seven days.

2. CSDS procedure

CSDS protocol was conducted as previously described with minor modification(Ferle et al. 2020, Golden, et al. 2011, Wang, et al. 2016). Briefly, for ten consecutive days, experimental male C57BL/6J mice (intruder) were subjected to physical interaction and defeat by the aggressive CD1 (resident) in residents' home cage (also called fighting cage as described above) for 5-10 min (one defeat stress per day). After physical defeat, intruder mice remained in the aggressor's home-cage on the other side of a perforated translucent Plexiglas divider to allow visual, auditory and olfactory interaction between intruder and resident for 24 h. Intruder mice faced novel CD1 mice every day. C57BL/6J mice in control group were housed together separated by the Plexiglas divider barrier and switched each day. They were never exposed to the CD1 mice. After 10 days of CSDS, all C57BL/6J mice were singly housed and tested for social interaction behavior within 24h.

3. Behavioral tests

3.1 Social interaction test (SIT)

This test was measured using the two-phase SIT(He et al. 2018). In the first phase called target-absent session, C57BL/6J mice were placed in an open field (44 cm × 44 cm × 30 cm) with an empty Plexiglas wire mesh enclosure (10 cm × 6.5 cm × 18 cm). The time spent in the interaction zone (IZ) surrounding the wire mesh enclosure was measured over 2.5 min. Mice were then returned to home cage for 30 s. In the second phase called target-present session, an unfamiliar CD1 mouse was placed inside the wire mesh and the same metrics was measured. From these two stages, an SI ratio was calculated. SI ratio is computed as the ratio of occupancy time in the IZ during the target-present session to that during the target-absent session. Mice with ratio < 1 were defined as susceptible and those with ratio ≥ 1 were defined as resilient(Golden, et al. 2011). Resilient mice were excluded from subsequent multi-omics experiments.

3.2 Sucrose preference test (SPT)

The SPT was used to assess anhedonia, an index of depressive-like behavior(Wu et al. 2016). After CSDS, mice were deprived from food and water for 24h. Subsequently, mice were provided with two bottles of water, one containing 1% sucrose solution and another containing pure water. To eliminate side preference, bottles were randomly placed on the left or right side of the cage to eliminate side preference. The following 24-h consumptions of 1% sucrose solution and pure water were assessed.

Sucrose preference was defined as [(sucrose intake / (sucrose intake + water intake)]. Body weight was measured immediately after SPT.

3.3 Open field test (OFT)

This test was used to assess anxiety-like behavior(Mao et al. 2017). In brief, mice were individually placed in the center of a white box (44 cm × 44 cm × 30 cm) with the head towards the same direction. After a 30-s latency, locomotor activity (total distance travelled) and movements in the central zone (central distance/time/entry number) over a 5.5-min period were measured. The box was cleaned with alcohol between tests to remove olfactory cues.

3.4 Elevated plus maze (EPM)

This test was used to assess anxiety-like behavior(Wang et al. 2020). The maze was composed of two open arms (30 cm × 6 cm) and two closed arms (30 cm × 6 cm × 15 cm). A central 6 cm× 6 cm square platform had access to all arms. Mice were placed in the center of the maze, habituated for 30 s and recorded the duration in each arm in the next 5 min. The maze was cleaned with alcohol between tests to remove olfactory cues.

3.5 Tail suspended test (TST)

The TST was used to evaluate behavioral despair and reflected by the duration of immobility(Wang, et al. 2020). Mice were individually suspended by their tails using a small piece of adhesive tape, which was placed 2 cm from the tip of the tail. After a 30-s latency, the duration of immobility was analyzed in a 5.5-min period. Mice were considered to be immobile only when they remained suspended passively and were completely motionless.

3.6 Forced swimming test (FST)

The FST was used to assess behavioral despair of mice(Tang et al. 2019). In this procedure, mice were placed individually in a Plexiglas cylinder (30 cm in height × 15 cm in diameter) filled with water (24 ± 1℃) to a depth of 18cm. After a 30-s latency, total immobility time in a 5.5-min period was calculated. The water was completely replaced after each test.

4. 16S rRNA gene sequencing

4.1 PCR amplification

The parameters of PCR amplification were set as follows: 3 min of denaturation at 95 °C; 27 cycles of 30 s at 95 °C, 30s for annealing at 55 °C, and 45s for elongation at 72 °C; and a final extension at 72 °C for 10 min, 10 °C until halted.

4.2 Quality-control of the raw gene sequences

The Raw sequencing data were quality-controlled by Trimmomatic and spliced by FLASH with

the following criteria: (i) Reads were truncated at any site receiving an average quality score less than 20 over a 50 bp sliding window. (ii) Primers were exactly matched allowing 2 nucleotides mismatching and reads containing ambiguous bases were removed. (iii) Sequences overlapping longer than 10 bp were spliced according to their overlap segments and those cannot be spliced were removed.

5. Metabolome analysis based on GC-MS

This protocol was in accordance with the previous study (Yang et al. 2019), with minor modification.

5.1 MCF derivatization

Fecal extracts were derivatized based on a methyl chloroformate (MCF) approach. Dried fecal extracts of each sample were dissolved with 200 μL of sodium hydroxide (1 M). After 10 s of vortexing, 167 μL of methanol and 34 μL of pyridine were added into tubes. Subsequently, 20 μL of MCF was added, followed by 30 s of vortexing, and this process was repeated once again. After that, 400 μL of chloroform and 400 μL of sodium bicarbonate (50 mM) were added and vortexed to isolate derivatized metabolites from the reactive mixture. After centrifugation at 500 g for 10 min the bottom chloroform phase was extracted for GC-MS analysis.

5.2 GC-MS analysis

GC-MS analysis was performed using a GC7890 system coupled to a MSD5975 mass selective detector (Agilent, California, USA) with electron impact ionization (70 eV). The GC was equipped with a gas capillary column (ZB-1701, 30 m × 250 μm id × 0.15 μm with 5 m guard column, Phenomenex, California, USA). The parameters of GC oven and MS were operated according to a previous report (Smart et al. 2010). Derivatized samples were injected in a pulsed splitless mode and the temperature of the injector was programmed at 290 °C. Temperatures of the auxiliary, MS quadrupole, and MS source were set at 250 °C, 230 °C, and 150 °C, respectively. The flow rate of helium gas was 1 mL/min. A scan speed of 1.562 μ/s was applied covering a mass range from 30 to 550 μm. The solvent delay was set to 5.5 min.

5.3 Metabolite identification and normalization

Metabolite deconvolution and identification were conducted by AMDIS software referring to the in-house library in the State Key Laboratory of Maternal and Fetal Medicine of Chongqing Municipality (Chongqing Medical University). The compounds were identified based on the following criteria: (i) more than 85% match to the in-house library spectra and (ii) within a one-minute window of the respective chromatographic retention time. The relative concentration of each metabolite was extracted by the in-house MassOmics R-based package and normalized by the internal standard and the dried weight of each sample.

6. Lipidome analysis based on LC-MS

The LC-MS/MS analysis process was performed based on a UPLC system (Nexera LC-30A, SHIMADZU) equipped with a Q-Exactive Plus (Thermo Scientific). A reverse-phase CSH C18 column (Waters, ACQUITY UPLC CSH C18, 1.7 µm, 2.1 mm× 100 mm) was used. The parameters of analyses were as follows: inject volume: 2 µL; column temperature: 45 °C; mobile phase A: 10 mM aqueous solution of ammonium acetonitrile formate (acetonitrile: water = 6:4, v/v); mobile phase B: 10 mM propyl alcohol solution of ammonium acetonitrile formate (acetonitrile: isopropanol =1:9, v/v); the elution gradient procedure: 30% B for 7 min, a linear increase in B from 30 to 100% over 18 min and 30% B for 5 min; the flow rate was 300 µL/min.

The mass spectrometer was operated in the negative and positive ion mode: (i) positive: Heater Temp 300 °C, Sheath Gas Flow rate 45 arb, Aux Gas Flow Rate15 arb, Sweep Gas Flow Rate 1arb, spray voltage 3.0 KV, Capillary Temp 350 °C, S-Lens RF Level 50%. MS^1 scan ranges: 200-1800; (ii) negative: Heater Temp 300°C, Sheath Gas Flow rate 45arb, Aux Gas Flow Rate 15arb, Sweep Gas Flow Rate 1arb, spray voltage 2.5KV, Capillary Temp 350 °C, S-Lens RF Level 60%. MS^1 scan ranges: 250-1800. The resolution of MS^1 and MS^2 were set as 70,000 and 17,500, respectively.

References

Ferle V, Repouskou A, Aspiotis G, Raftogianni A, Chrousos G, Stylianopoulou F, et al. (2020). Synergistic effects of early life mild adversity and chronic social defeat on rat brain microglia and cytokines. *Physiology & behavior*. 215:112791. doi:10.1016/j.physbeh.2019.112791

Golden SA, Covington HE, 3rd, Berton O, Russo SJ. (2011). A standardized protocol for repeated social defeat stress in mice. *Nature protocols*. 6:1183-1191. doi:10.1038/nprot.2011.361

He Y, Li W, Tian Y, Chen X, Cheng K, Xu K, et al. (2018). iTRAQ-based proteomics suggests LRP6, NPY and NPY2R perturbation in the hippocampus involved in CSDS may induce resilience and susceptibility. *Life sciences*. 211:102-117. doi:10.1016/j.lfs.2018.09.016

Mao Q, Gong X, Zhou C, Tu Z, Zhao L, Wang L, et al. (2017). Up-regulation of SIRT6 in the hippocampus induced rats with depression-like behavior via the block Akt/GSK3beta signaling pathway. *Behavioural brain research*. 323:38-46. doi:10.1016/j.bbr.2017.01.035

Smart KF, Aggio RB, Van Houtte JR, Villas-Boas SG. (2010). Analytical platform for metabolome analysis of microbial cells using methyl chloroformate derivatization followed by gas chromatography-mass spectrometry. *Nature protocols*. 5:1709-1729. doi:10.1038/nprot.2010.108

Tang M, Huang H, Li S, Zhou M, Liu Z, Huang R, et al. (2019). Hippocampal proteomic changes of susceptibility and resilience to depression or anxiety in a rat model of chronic mild stress. *Transl Psychiatry*. 9:260. doi:10.1038/s41398-019-0605-4

Wang W, Guo H, Zhang SX, Li J, Cheng K, Bai SJ, et al. (2016). Targeted Metabolomic Pathway Analysis and Validation Revealed Glutamatergic Disorder in the Prefrontal Cortex among the Chronic Social Defeat Stress Mice Model of Depression. *J Proteome Res*. 15:3784-3792. doi:10.1021/acs.jproteome.6b00577

Wang W, Wang T, Bai S, Chen Z, Qi X, Xie P. (2020). Dl-3-n-butylphthalide attenuates mouse behavioral deficits to chronic social defeat stress by regulating energy metabolism via AKT/CREB signaling pathway. *Transl Psychiatry*. 10:49. doi:10.1038/s41398-020-0731-z

Wu Y, Tang J, Zhou C, Zhao L, Chen J, Zeng L, et al. (2016). Quantitative proteomics analysis of the liver reveals immune regulation and lipid metabolism dysregulation in a mouse model of depression. *Behavioural brain research*. 311:330-339. doi:10.1016/j.bbr.2016.05.057

Yang Y, Yin Y, Chen X, Chen C, Xia Y, Qi H, et al. (2019). Evaluating different extraction solvents for GC-MS based metabolomic analysis of the fecal metabolome of adult and baby giant pandas. *Sci Rep*. 9:12017. doi:10.1038/s41598-019-48453-1
